# Choline Halide-Based Deep Eutectic Solvents as Biocompatible Catalysts for the Alternating Copolymerization of Epoxides and Cyclic Anhydrides

**DOI:** 10.1021/acssuschemeng.3c06766

**Published:** 2024-04-30

**Authors:** Mary Dana Czarinah L. Cheng-Tan, Angelyn N. Nguyen, Collette T. Gordon, Zachary A. Wood, Yvonne Manjarrez, Megan E. Fieser

**Affiliations:** † Department of Chemistry, University of Southern California, Los Angeles, California 90089, United States; ‡ Wrigley Institute for Environment and Sustainability, University of Southern California, Los Angeles, California 90089, United States

**Keywords:** choline chloride, ring-opening copolymerization, polyesters, biocompatible catalysts, deep eutectic solvents

## Abstract

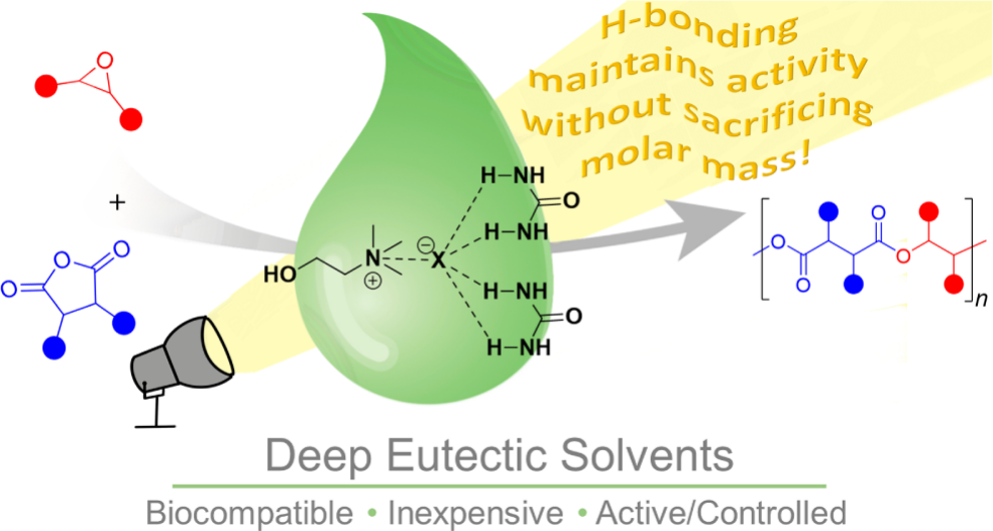

Aliphatic polyesters have received considerable attention in recent years due to their biodegradability and biocompatible, mechanical, and thermal properties that can make them a suitable alternative to today’s commercialized polymers. The ring-opening copolymerization (ROCOP) of epoxides and cyclic anhydrides is a route to synthesize a diverse array of polyesters that could be useful in many applications. However, the catalysts used rarely consider biocompatible catalysts in the case that any are left in the polymer. To the best of our knowledge, we report the first example of using deep eutectic solvents (DESs) as biocompatible catalysts for this target ROCOP with polymerization activity for at least six diverse monomer pairs. Choline halide salts are active for this polymerization, with dried salts showing polymerization slower than that of those conducted in air. Hydrogen bonding with water is hypothesized to enhance the rate-determining step of epoxide ring opening. While the presence of water improves the rate of polymerization, it also acts as a chain transfer agent, leading to smaller molar mass polymers than intended. Combining the choline halide salts with urea or ethylene glycol hydrogen bond donors in air led to DES catalysts that reacted similarly to the salts exposed to air. However, when generating these DESs in air-free conditions, they showed similar rates of polymerization without a drop in polymer molar mass. The hydrogen bonding provided by urea and ethylene glycol seems to promote the rate increase without serving as a chain transfer agent. Results reported herein display the promising potential of biocompatible catalyst systems for this ROCOP process as well as introducing the use of hydrogen bonding to enhance polymerization rates.

## Abstract 2

Deep eutectic solvents are identified as bio-friendly and inexpensive catalysts for the synthesis of polyesters via copolymerization of epoxides and cyclic anhydride.

## Introduction

Aliphatic polyesters are receiving interest for use in disposable packaging and medical applications, as they are often biofriendly and easy to degrade. These polymers are often made by the ring-opening polymerization of cyclic esters.^1−4^ For example, poly(lactic acid) (PLA) is used in food packaging and biomedical applications.^5−8^ However, the limitations in cyclic ester structures have often limited the physical properties of the polymers and therefore the potential use of these materials.^9,10^ The most common polymers that are Food and Drug Administration (FDA)-approved for use in biomedical applications are PLA, poly(glycolic acid), and poly(caprolactone), which are restricted in their applications due to their specific physical properties, crystallinity, and biodegradation rates.^6,8,11−18^ Therefore, it would be valuable to increase the diversity of biofriendly polyesters available for these applications.

**Figure 1:**
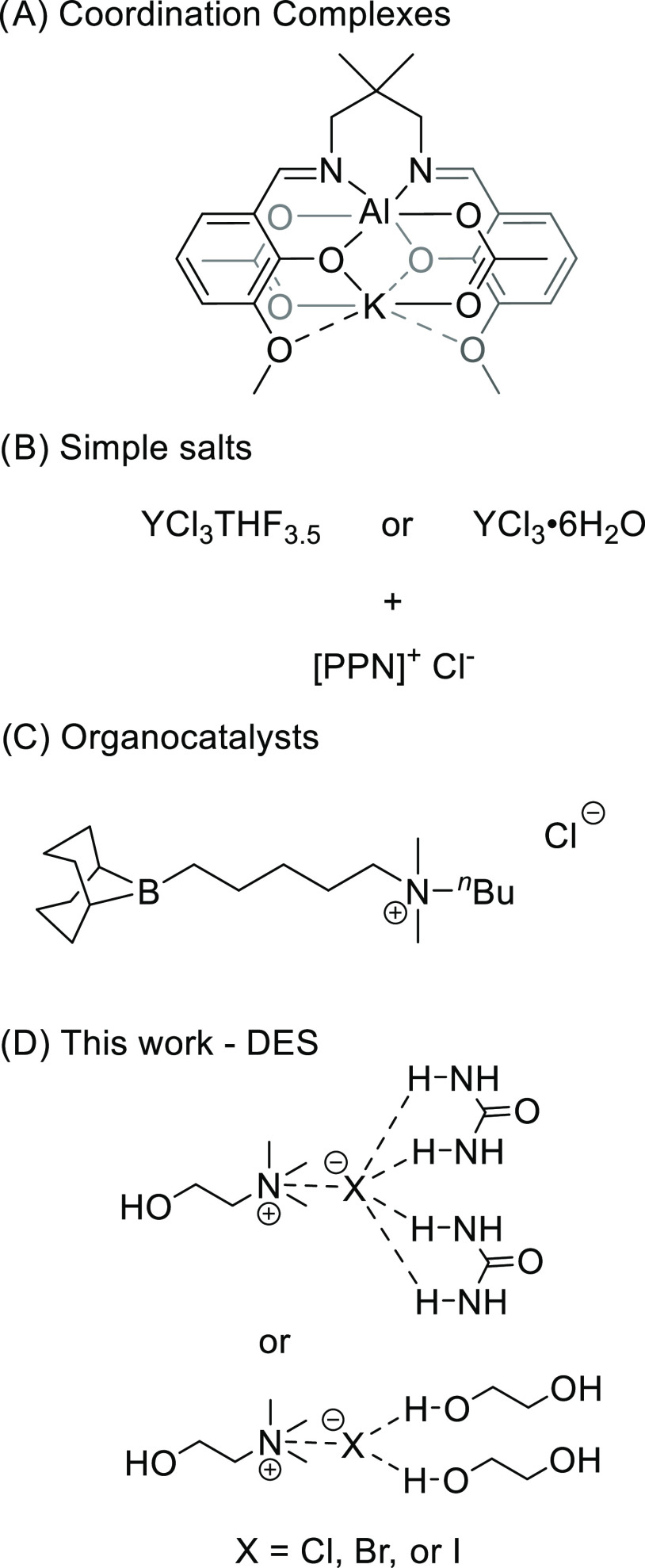
Previously used catalysts for ROCOP of epoxides and cyclic anhydrides. (A) Heterodinuclear catalyst.^24,26^ (B) Simple salt catalyst.^29,30^ (C) Organoboron catalyst.^43^ (D) This work used deep eutectic solvents as a catalyst.

The ring-opening copolymerization (ROCOP) of epoxides and cyclic anhydrides is a direction toward more diverse polyester structures. There have been over 20 epoxides and 20 anhydrides used in this polymerization, some of which can be biosourced, resulting in over 400 different possible polyester structures.^19−23^ The introduction of even just one monomer being biosourced presents itself as a better alternative than petroleum-derived sources. In addition to the likelihood of polyesters degrading into typically harmless byproducts and their high potential for biocompatibility, this can be a more sustainable alternative to current petroleum-based polymers, which can also minimize the environmental footprint of polymer production.^19^ Catalyst design has centered on the use of metal-based catalysts with designer ligands to identify the desired fast rates (often determined through single-point turnover frequencies (TOF)), dispersity control, and the ability to acquire high-molar mass polymers. These design successes are often due to tethering two cocatalysts together, allowing rates to be maintained even at low catalyst loading, as advanced by the Coates and Williams groups (Figure 1A).^24−28^ We recently identified that simple rare earth metal salts, in combination with a cocatalyst, were able to maintain fast polymerization rates and excellent polymerization control, without the need for a designer ligand (Figure 1B).^29,30^ It is evident that these catalysts could be air-stable, with the presence of water aiding in the increase of the rate of polymerization for most monomer pairs. However, the presence of water can also be detrimental, as it leads to lower polymer molar masses than desired. In addition, the catalyst system required the use of bis(triphenylphosphoranylidene)ammonium chloride ([PPN]Cl), an expensive and toxic cocatalyst, in order to reach the target ROCOP products.^31,32^ An alternative inexpensive cocatalyst, such as a phosphonium chloride ionic liquid, led to air-stable, metal-containing ionic liquid catalysts that not only were faster at polymerization but also yielded high-molar mass polymers with greater ease.^33^

Advances have been made to optimize polymerization rate, polymerization control (preventing side reactions, maintaining dispersity control, and molar mass control), and the ability to make high-molar mass polymers. However, in the case in which any catalyst remains enmeshed in the polymer, the toxicity of the catalyst is rarely considered. Satoh and co-workers made great strides in biofriendly catalysts when identifying sodium and potassium acetate, commonly used as food additives, as active for the target ROCOP, as well as the ring-opening polymerization of cyclic esters.^34−36^ Furthermore, it has been shown by Li et al. and Coulembier et al. that potassium acetate was an active catalyst for both ROCOP and ring-opening polymerization to make aliphatic polyesters.^37,38^

While this was an exciting direction for catalyst design, Satoh and co-workers have determined that increasing the size of the cation and changing the carboxylate moiety to a more electron-donating group enhance turnover frequency (TOF). Thus, cesium pivalate was identified as the most active salt in the series tested. The effect of increasing the cation size was evident when comparing the TOFs between sodium (TOF = 16.8 h^–1^), potassium (TOF = 42.5 h^–1^), and cesium (TOF = 44.6 h^–1^) acetate for the ROCOP of phthalic anhydride and ethyl glycidyl ether. Similarly, the impact of the electron-donating group can be seen when comparing the metal acetates and metal pivalates, wherein a significant increase was seen between potassium (TOF = 46.5 h^–1^) and cesium (TOF = 68.6 h^–1^) pivalate compared to metal acetates above.^34^ Therefore, while potassium acetate would be much more biofriendly compared to cesium pivalate, priority has been placed on the rate and control of polymerization.^39,40^ Even with the cesium salts, higher molar mass polymers could be achieved only with an extreme excess of monomers to the catalyst.

Other groups have explored the use of boron-, phosphorus-, and nitrogen-based main groups or organocatalysts as an emerging area, although most of the catalysts have yet to outcompete metal catalysis in terms of polymerization rate and control (Figure 1C). Many of these catalysts still use toxic [PPN]Cl as a cocatalyst with boron Lewis acids.^41−54^ An organoboron-ammonium catalyst from Wu and co-workers achieved a TOF of 258 h^–1^ with phthalic anhydride (PA) and cyclohexene oxide (CHO) at 120 °C for 40 min.^43^ With increased temperature to 180 °C and reacting for 10 min, there was a significant increase in TOF (TOF = 816 h^–1^). Tao and co-workers have found that a thiourea-boron catalyst with [PPN]_2_BDC (BDC = 1,2-benzenedicarboxylate) as the initiator achieved a TOF of 408 h^–1^ at 90 °C and 10 min for propylene oxide (PO) and PA, although when used with CHO and PA polymerization at 90 °C for 40 min, there was a noticeable decrease in TOF (TOF = 299 h^–1^).^51^ In another study with Meng and co-workers, a thiourea catalyst with [PPN]Cl attained a TOF of 456 h^–1^ at 110 °C in 10 min for CHO and PA.^54^ These advances in organocatalyst design for ROCOP have also maintained good dispersity control and minimal side reactions. While some comparisons can be made, TOFs are challenging to directly compare between catalysts in the literature due to variations in testing conditions such as the temperature, catalyst loading, monomer pairs, and polymerization time. Notably, catalysts either use [PPN] salts, include toxic components, or require synthetic steps with toxic reagents or wasteful chemical use.^41−54^

**Scheme 1:**
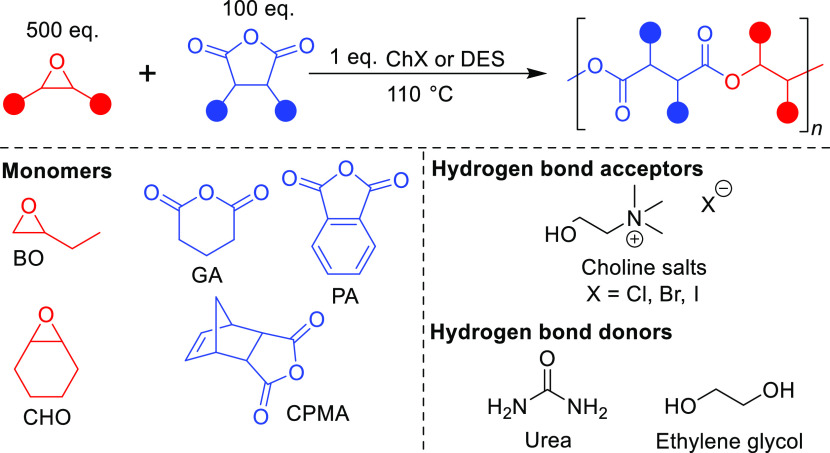
General Reaction Scheme with the Monomer and Catalyst Scope

In this work, we investigated the use of deep eutectic solvents (DESs) as an alternative biofriendly route to make aliphatic polyesters (Figure 1D and Scheme 1). DESs are emerging solvents that have applications ranging from extraction and separation of metals and organic compounds to drug delivery and catalysis and are considered to be more sustainable than a selection of standard solvents.^55−57^ These solvents have an unusually low melting point compared to their individual components, consisting of pairs of hydrogen bond donors and hydrogen bond acceptors.^58^ To the best of our knowledge, DESs have not yet been explored for the catalysis of ROCOP of epoxides and cyclic anhydrides. In addition to their inexpensiveness and ready availability of their constituents in bulk, DESs are environmentally friendly. They have a facile synthesis, wherein a 1:2 molar ratio combination of hydrogen bond acceptor to donor is used under neat and mild conditions.^55−60^ This is unlike the case for the previously mentioned organocatalysts. For instance, the catalysts developed by Tao and co-workers require the use of multiple solvents and column chromatography, resulting in an overall yield of ∼66%.^51^ Similarly, while the catalyst produced by Wu and co-workers required less extensive synthesis, it maintained the use of solvents and the need for purification.^43^ Both these catalysts also require the use of 9-borabicyclo(3.3.1)nonane, which is toxic.^43,51,61^ Additionally, it was hypothesized that the hydrogen bonding in DESs could provide the same benefits that water provides to the rate of polymerization, as discussed above, without sacrificing the molar mass of the polymer.

We took an interest in choline chloride (ChCl) as it is inexpensive and biocompatible, acknowledged by the FDA for its approval as a human nutrient and use as an animal feed nutrient.^62^ Its facile synthesis with urea to form DESs, with low expected toxicity, makes this system ideal for work that needs biocompatible polymers.^63−65^ Herein, we describe choline salts and choline-based deep eutectic solvents as biofriendly, active catalysts for the ROCOP of several epoxide and cyclic anhydride monomer pairs. The importance of the DESs, the anion, and synthetic conditions for DESs as it relates to the rate of polymerization, polymerization control, and ability to access high-molar mass polymers will be discussed. The hydrogen bonding present in the DES, even under air-free conditions, allows for catalysis that has the same rate of polymerization with and without water presence, while still allowing high-molar mass polymers to be synthesized.

## Results and Discussion

To assess the versatility of the catalytic system, a diverse set of monomers was initially used. Carbic anhydride (CPMA), phthalic anhydride (PA), and glutaric anhydride (GA) were chosen as representative mono-, bi-, and tricyclic anhydrides. 1-Butene oxide (BO) and cyclohexene oxide (CHO) were used as they are the most commonly used mono- and bisubstituted epoxides, with BO serving as a higher temperature-boiling alternative to PO. Since there is no one-size-fits-all catalyst for all monomer pairs, these five monomers represent the common variations in the monomer structure that can lead to various polymerization activities for a catalyst.

### Choline Halides

Initially, the activity of choline chloride (ChCl), choline bromide (ChBr), and choline iodide (ChI) as catalysts for the target ROCOP was screened. Past studies identified that some organocatalysts do not require cocatalysts to function.^43,46,47^ While these choline salts were often not entirely soluble in the polymerization reactions, all were active for all six monomer pairs, as identified by ^1^H nuclear magnetic resonance (NMR) spectroscopy. Single-point turnover frequencies ranged from 34 to 200 h^–1^ (Table 1) depending on the monomer pair, which are moderate TOFs for the field. Much like the trends seen with rare earth metal salts in the literature, polymerizations with the bicyclic PA anhydride were the fastest, while polymerizations with the monocyclic GA and tricyclic CPMA were slower.^29^ With this being a common trend with several catalysts, this observation is likely due to the nucleophilicity of the carboxylate, resulting from the cyclic anhydride ring opening, toward the ring opening of the epoxide monomer.

**Table 1:**
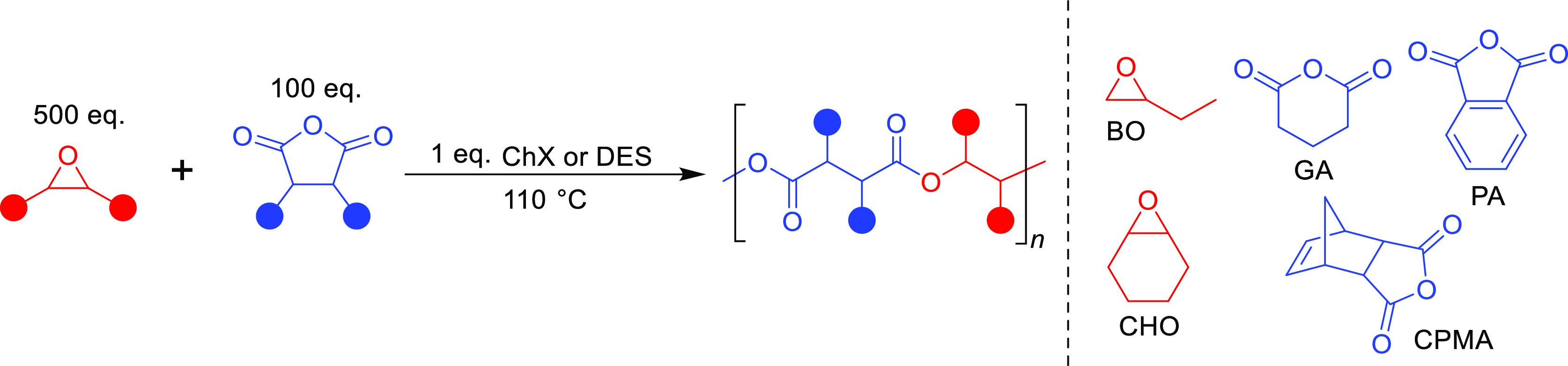
Polymerizations of Various Monomer Pairs with Choline Halide Catalysts^a^

entry	catalyst	anhydride	epoxide	conv.^b^ (%)	TOF^c^ (h^–1^)	ester selectivity^d^ (%)	epimer.^e^,^f^ (%)
**Air-exposed**							
1	ChCl	CPMA	BO	55 (14)	41 (10)	89 (3)	18
2		PA		89 (11)	179 (12)	>99	
3		GA		66 (5)	66 (5)	96 (6)	
4	ChCl	CPMA	CHO	77 (12)	58 (11)	>99	7
5		PA		94 (4)	187 (5)	89 (12)	
6		GA		64 (15)	64 (15)	88 (20)	
7	ChI	CPMA	BO	85 (10)	64 (8)	>99	7
8		PA		>99	200	>99	
9		GA		72 (3)	72 (3)	>99	
10	ChI	CPMA	CHO	77 (3)	58 (2)	96 (6)	13
11		PA		96 (2)	193 (5)	86 (11)	
12		GA		67 (4)	67 (4)	83 (14)	
13	ChBr	CPMA	BO	77 (3)	58 (2)	95 (8)	8
14		PA		97 (3)	193 (2)	>99	
15		GA		81 (13)	81 (13)	98 (1)	
16	ChBr	CPMA	CHO	66 (3)	66 (2)	95 (7)	11
17		PA		98 (2)	196 (3)	82 (6)	
18		GA		63 (3)	63 (3)	82 (15)	
**Air-free**							
19	ChCl	CPMA	BO	45 (7)	34 (5)	91 (2)	ND
20		PA		64 (24)	128 (49)	>99	
21		GA		63 (10)	63 (10)	>99	
22	ChCl	CPMA	CHO	17 (10)	13 (8)	89 (8)	ND
23		PA		68 (19)	135 (45)	88 (6)	
24		GA		54 (3)	54 (3)	>99	

a The [catalyst]/[anhydride]/[epoxide] was 1:100:500. Reactions were heated to 110 °C under neat conditions. The polymerizations with CPMA ran for 80 min, with PA for 30 min and GA for 60 min unless otherwise noted. All reactions were done in triplicate or more.

b Determined using ^1^H NMR spectra of crude reaction mixtures, comparing the conversion of anhydride monomers to polymers.

c Defined as (mol anhydride consumed)/(mol catalyst) × h.

d Ester selectivity was determined by using the ^1^H NMR spectra of in situ polymers, comparing the polyether signal to a polyester signal.

e Determined using ^1^H NMR spectra of purified polymers with % epimer. = {2 × A_2.7 ppm_/(A_6.0–6.5 ppm_)} × 100. Epimerization indicated is for a single sample.

f In some cases (indicated by “ND”), conversions were too low to isolate polymers; therefore, epimerization was not quantified.

Although the anion might be expected to impact the initiation of polymerization, no differences were observed in the polymerization conversions, as all three salts showed results mostly within error of each other for all six monomer pairs. These results indicate that initiation is likely rapid or that the halide is not involved in the initiation. Polyester content was consistently higher than 80%, and under various conditions, the BO monomer showed less sign of epoxide homopolymerization than the CHO monomer. The higher presence of homopolymerization of the disubstituted CHO monomer is consistent with the prior literature.^29^ The BO/CPMA and CHO/CPMA monomer pairs also showed small amounts of epimerization (Table 1). Percent epimerization was calculated via ^1^H NMR by comparing the epimer peaks with two protons in the polymer corresponding with the CPMA ring (Figure S5).

**Figure 2:**
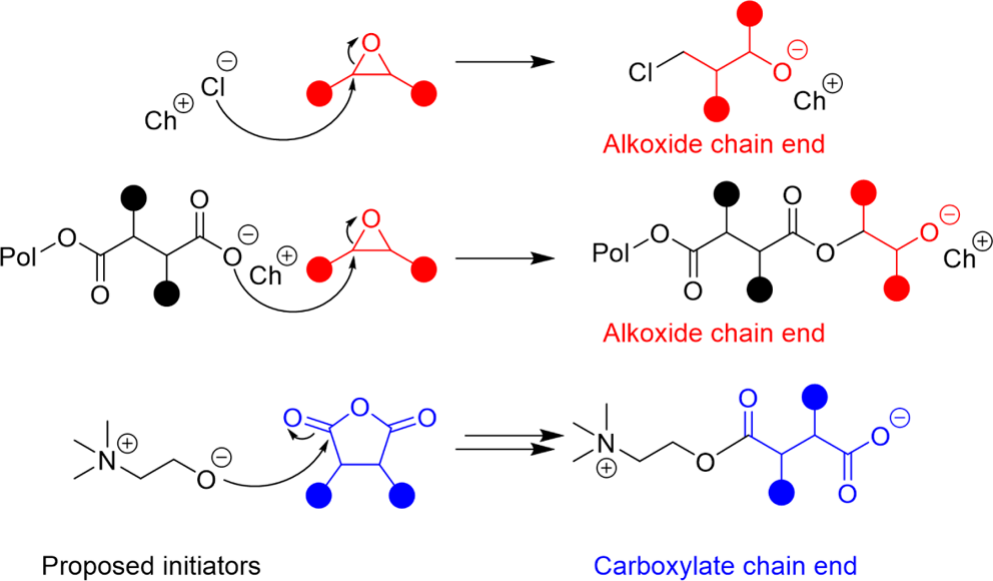
Proposed mechanism for how potential initiators enter the catalytic cycle. Note that the alkoxide generated from chloride ring opening with epoxide is hypothesized to deprotonate carboxylic acid or alcohol groups through chain transfer reactions. The choline cation and/or hydrogen bond donors (water, urea, and ethylene glycol) are expected to influence either or both the activation of epoxide and nucleophilicity of the carboxylate anion, depending on the catalyst. The full interaction is not directly shown here, as it has not yet been discerned.

Since these salts were used in air, as they are air-stable, the presence and impact of water cannot be ignored. First, water acts as a chain transfer agent by interrupting the ongoing chain, resulting in more smaller chains than expected for the halide initiators. Water can also open epoxide or cyclic anhydride monomers to diols or dicarboxylic acids, which can also act as chain transfer agents. If water, diol, or diacids are the most prominent initiators in this system, it would explain why there was no difference between the halide initiators. Using thermogravimetric analysis (TGA) to analyze the water content, as described in the SI, 0.43 equiv of water for every ChCl is observed. However, it is well known that ChCl is hygroscopic and will absorb additional water when exposed; therefore, determining the amount of water present for every reaction is difficult. Nonetheless, it is clear that water is present in this salt. Analysis of the polymer molar mass for the BO/CPMA monomer pair was pursued with ChCl since it is the most biorelevant salt. The BO/CPMA monomer pair was selected, as it is the easiest to purify from water and diacids, with BO having a lower boiling point than that of CHO and CPMA, forming well-defined crystals during recrystallization. As expected, analysis of the polymers by gel permeation chromatography (GPC) showed a much lower molar mass than that calculated for just halide initiators while maintaining a unimodal molar mass distribution (Table 2, entry 1, and Table S1, entries 1a–1d). All GPC results shown in Table 2 were characterized on all replicate polymers, and the results discussed are reproducible. Matrix-assisted laser desorption ionization time-of-flight mass spectrometry (MALDI-TOF-MS) studies indicate three primary species, which are not conclusive for specific initiating groups. However, the end group molar masses are most closely comparable to chloride, choline alcohol, and ring-opened cyclic anhydride initiating species. These could all be reasonable in this particular reaction, although NMR characterization of polymers and oligomers has not shown conclusive evidence for choline remaining with the polymer. The proposed initiation options are shown in Figure 2, wherein both the chloride and ring-opened anhydride ring-open an epoxide, likely activated by the choline cation, to produce an alkoxide chain end. For the choline-initiated polymer, the alcohol of the choline is expected to serve as the initiator to ring-open a cyclic anhydride. It is unclear if the deprotonation of the choline alcohol or dicarboxylic acid occurs before or after the ring opening of the cyclic anhydride. Since Cl is shown to initiate polymerization through epoxide ring opening, the generated alkoxide could deprotonate the choline alcohol or a carboxylic acid, essentially conducting a chain transfer. The generated carboxylate anion could then ring-open an activated epoxide, while the generated alkoxide could ring-open a cyclic anhydride. Activated monomer mechanisms with the carboxylic acid or choline alcohol could also be plausible, although the existence of two mechanisms occurring in the same pot (since chloride supports anionic ring-opening polymerization) is hypothesized to be less likely.

**Table 2:**
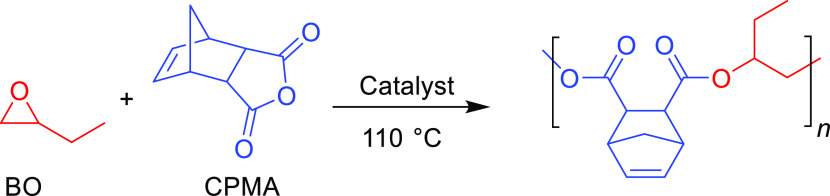
Molar Mass and Dispersity Comparison between Copolymerization with BO and CPMA with Various Catalysts^a^

entry	catalyst	time	conv. (%)^b^	ester selectivity^c^ (%)	*M*_n,theo_^d^,^e^	*M*_n,exp_^e^,^f^	*Đ*^e^,^f^
**Air-exposed**							
1	ChCl	80 min	55 (14)	89 (3)	12.9	1.3	1.1
2	ChCl/Urea	80 min	46 (18)	76 (20)	17.2	3.6	1.3
3	ChCl/EG	80 min	86 (4)	82 (22)	21.0	1.9	1.0
**Air-free**							
4	ChCl	2 h	64 (7)	96 (4)	16.0	48.6	1.6
5	ChCl/Urea	80 min	65 (9)	93 (8)	15.1	15.6	1.6
6	ChCl/EG	80 min	76 (7)	>99 (1)	16.1	11.6	1.4

a The [catalyst]/[CPMA]/[BO] was 1:100:500. Reactions were heated to 110 °C under neat conditions. Unless otherwise noted, information is listed for reactions done in at least triplicates.

b Determined using ^1^H NMR spectra of crude reaction mixtures, comparing the conversion of anhydride monomers to polymers.

c Ester selectivity was determined by using the ^1^H NMR spectra of *in situ* polymers, comparing the polyether signal to a polyester signal.

d Calculated for 1 chloride initiator.

e GPC data for a single sample.

f Identified by GPC, using a Wyatt MALS detector.

Water has also been shown to increase the rate of polymerization, particularly in the case of metal ionic liquid catalysts.^33^ This has been hypothesized to polarize the carboxylate nucleophile through hydrogen bonding to enhance ring opening of the epoxide, which has often been found to be the rate-determining step of the reaction. Some studies have shown that thiourea catalysts used for the ROCOP of epoxides with cyclic anhydrides act as hydrogen bond donors for monomer activation.^51,54^ Tao and co-workers have also investigated the Gibbs free energy calculations for the ROCOP of PO and CPMA with a thiourea-boron catalyst, determining that without thiourea, the transition-state energy barrier to form the alkoxide from ring opening of the epoxide is significantly higher than that with thiourea. This suggests that the hydrogen bonding provided by the thiourea facilitates the ring opening of epoxides for the ROCOP.^51^

To help elucidate the impact of initiators, the choline halides were dried on a Schlenk line at 100 °C for 5 days and stored in the glovebox once water was confirmed to be eliminated by ^1^H NMR spectroscopy. The same TGA analysis of air-free ChCl, as discussed above, revealed no measurable water presence, in agreement with the NMR studies. Polymerization of the six monomer pairs with these three dried choline halides all showed active polymerization (Table 1, entries 19–24, and Table S10–S12). Again, no specific trends could identify any as superior to another. Therefore, ChCl was further prioritized as it is the least expensive and most biorelevant choline salt. While not true for every salt and monomer pair, prior cases with rare earth metal salts and metal ionic liquids identified the anhydrous catalysts to be slower than those of the catalysts stored in air.^29,33^ Results with air-free ChCl show the same slower polymerization rate than ChCl stored under air. This aligns with the hypothesis that water helps accelerate polymerization rates. Without water, the selectivity for ROCOP over epoxide homopolymerization is similar to that of the air-exposed polymers. To determine if ChCl itself is active for the homopolymerization of BO or CHO, 1 equiv of ChCl with 500 equiv of epoxide was heated and stirred at 110 °C for 30 min, which resulted in <1% epoxide homopolymerization for both epoxides as the catalyst was only slightly soluble.

Characterizing the BO-*alt*-CPMA polymer with dried ChCl by GPC showed a much larger molar mass (*M*_*n*_) than that expected (Table 2, entry 4, and Table S10, entries 1d–1f). A bimodal molar mass distribution (Figure S55) indicates the likelihood of a small amount of water still present in the salt that can impact the initiation as a chain transfer agent. The larger molar mass than that expected indicates that not all initiators are initiating polymer chains, presumably due to the lower solubility of these salts when dried and stored away from air and moisture. MALDI-TOF-MS studies also show a bimodal molar mass distribution. The major low-molar mass fragment mostly aligns with the air-exposed ChCl catalyst, with end groups matching the closest to those of choline alcohol and chloride initiators. The third species shows an end group with a mass slightly higher than that of the hypothesized ring-opened cyclic anhydride initiators proposed for air-exposed ChCl. However, an alternative reasonable assignment for this initiating species has not been identified. Even though the CPMA monomer is crystallized and sublimed, residual ring-opened cyclic anhydride could be present in the monomer. The minor higher molar mass fragment shows end groups near that expected for ring-opened epoxide and ring-opened cyclic anhydride initiating species.

### Deep Eutectic Solvents (DESs)

Since DESs have hydrogen bonding from reagents other than water and are liquids at low temperatures, we hypothesized that it might be possible to achieve reasonable TOFs and controlled molar masses of polymers (meaning the ability to synthesize molar masses as expected from the number of halide initiators) with them. This would essentially enable the value that water brings to the rate of polymerization without the undesirable chain transfer reactions. The liquid nature of the DESs was also hypothesized to improve initiation in comparison with the less soluble salts.

Therefore, DESs with the three choline halide salts with 2 equiv of urea or ethylene glycol (EG) were synthesized for all three choline salts by literature methods.^60−67^ These DESs were first tested for polymerization of the six target monomer pairs when exposed to air. Again, little to no difference was observed between the different halides, within error, for all monomer pairs (Tables S4–S9); therefore, ChCl-based DESs were prioritized (Table 3). Interestingly, both hydrogen bond donor DESs generally showed rates of polymerization similar to that of ChCl salt exposed to air (Table 3). Side reactions for the ChCl-based DESs were quite similar to those for the salts, including suppressed epoxide homopolymerization and minimal epimerization of CPMA-based polymers.

**Table 3:** Polymerizations of Various Monomer Pairs with DES Catalysts^a^,^f^

entry	catalyst	anhydride	epoxide	conv.^b^ (%)	TOF^c^ (h^–1^)	ester selectivity^d^ (%)	epimer.^e^^,^^f^ (%)
**Air-exposed**							
1	ChCl/Urea	CPMA	BO	46 (18)	35 (13)	76 (20)	ND
2		PA		72 (12)	144 (3)	96 (4)	
3		GA		63 (16)	63 (17)	>99	
4	ChCl/Urea	CPMA	CHO	66 (8)	50 (6)	97 (3)	5
5		PA		>99	198	78 (3)	
6		GA		72 (10)	72 (11)	70 (4)	
7	ChCl/EG	CPMA	BO	86 (4)	65 (3)	82 (22)	7
8		PA		>99 (1)	197 (5)	>99	
9		GA		73 (7)	73 (7)	97 (3)	
10	ChCl/EG	CPMA	CHO	75 (5)	56 (3)	>99	6
11		PA		98 (2)	196 (5)	78 (4)	
12		GA		75 (4)	75 (4)	74 (4)	
**Air-free**							
13	ChCl/Urea	CPMA	BO	65 (9)	49 (4)	93 (8)	12
14		PA		74 (3)	149 (12)	>99	
15		GA		55 (6)	55 (6)	>99	
16	ChCl/Urea	CPMA	CHO	50 (1)	38 (1)	93	ND
17		PA		94 (5)	189 (11)	87 (1)	
18		GA		55 (13)	55 (13)	>99	
19	ChCl/EG	CPMA	BO	76 (7)	57 (5)	>99 (1)	9
20		PA		97 (1)	195 (2)	>99	
21		GA		47 (14)	47 (14)	>99	
22	ChCl/EG	CPMA	CHO	65 (8)	49 (6)	>99 (1)	10
23		PA		73 (16)	146 (12)	91 (1)	
24		GA		52 (5)	52 (5)	>99	

a The [catalyst]/[anhydride]/[epoxide] was 1:100:500. Reactions were heated to 110 °C under neat conditions. The polymerizations with CPMA ran for 80 min, with PA for 30 min and GA for 60 min. All reactions were done in triplicates or more.

b Determined using ^1^H NMR spectra of crude reaction mixtures, comparing the conversion of anhydride monomers to polymers.

c Defined as (mol anhydride consumed)/(mol catalyst) × h.

d Ester selectivity was determined by using the ^1^H NMR spectra of in situ polymers, comparing the polyether signal to a polyester signal.

e Determined using ^1^H NMR spectra of purified polymers with % epimer. = {2 × A_2.7 ppm_/(A_6.0–6.5 ppm_)} × 100. Epimerization indicated is for a single sample.

f In some cases (indicated by “ND”), conversions were too low to isolate polymers; therefore, epimerization was not quantified.

The molar mass and dispersity of BO-*alt*-CPMA with ChCl/urea and ChCl/EG were analyzed (Table 2, entries 2 and 3, respectively, as well as Table S4, entries 1a–1e, and Table S5, entries 1a–1c), and we found that the molar masses were smaller than the theoretical molar masses, which was expected due to the presence of water. However, good dispersity was maintained, indicating that the formation of DES does not greatly impact polymerization control. BO-*alt*-CPMA catalyzed by ChCl/urea had a slightly bimodal molar mass distribution, while when catalyzed by ChCl/EG, it has a unimodal distribution (Figures S48–S49). TGA studies for the ChCl/urea DES revealed 1.17 equiv of water for every choline, while water could not be quantified for the ChCl/EG DES due to the volatility of EG (Figure S67). MALDI-TOF-MS of the polymers generally matched the findings of air-exposed ChCl, suggesting chloride, the choline alcohol, and ring-opened cyclic anhydride as the most likely candidates for initiating species. Since the end groups were consistent between the ChCl salt and the two DESs, all exposed to air, there was no evidence of initiation from urea or EG. The best comparison to the literature is polymerization with a [PPN]Cl/thiourea pair, in which the thiourea is hypothesized to help activate the epoxide and impact the reactivity of the anionic polymer chain end.^54^ In the case of these DESs, urea and EG are expected to do the same thing, using hydrogen bonding to impact the reaction without initiating a polymer chain.

The DESs were made under inert conditions, starting with materials dried and stored in a glovebox. Without exposure to air and moisture, the DESs with urea were waxy solids when cooled to room temperature, while EG DESs remained liquid at room temperature. TGA studies of the air-free ChCl/urea DES showed 0.41 equiv of water for every choline, which is lower than that measured for the air-exposed DES. Again, the water content of the air-free ChCl/EG DES could not be obtained due to the volatility of the EG. These air-free DESs have also been shown to be active for the polymerization of all six monomer pairs, with greater consistency in turnover frequency than with air-exposed DESs (Tables S13–S18). Comparing the TOF between the air-exposed and air-free DESs showed variable results, with no clear trend of which showed faster polymerization (Table 3). This suggests that in general, lack of water was not greatly disturbing the polymerization rate, and the hydrogen bond donor (urea or EG) was likely serving a similar purpose as water. In addition, the alternating frequency of the polymer is shown to be higher when done air-free compared to the air-exposed polymerizations. The molar mass of the BO-*alt*-CPMA polymer catalyzed with ChCl/urea (Table 2, entry 5, and Table S13, entries 1a–1e) was much closer to the theoretical molar mass than any others in this study. On the other hand, when catalyzed with ChCl/EG (Table 2, entry 6, and Table S14 entries 1a–1d), the experimental molar mass is lower than the anticipated value. However, the difference in the measured and expected molar masses is not as significant as that observed in the air-exposed polymer. This supports the hypothesis that in DESs, while the hydrogen bond donor may provide hydrogen bonding similar to water, it does not exhibit as much chain transfer behavior. Similar to the dried choline chloride, polymers catalyzed with the dried DESs showed a somewhat bimodal molar mass distribution with a larger molar mass shoulder, indicating that the dried DESs do still have some initiation from either residual water or an opened monomer, such as diacids and diols. However, MALDI-TOF-MS studies continue to match those of the air-exposed DESs, with end groups most closely matching those of the choline alcohol and chlorine initiators. Similar to the air-free ChCl-based polymer, the third species identified differed slightly from the air-exposed alternatives, although the reason for this difference is currently unknown. These results suggest that even without the presence of water, urea and EG do not seem to initiate polymer chains.

### Presence of the Catalyst in the Polymer

In these studies, the choline salt or DESs were easily separated from the polymer with no evidence of the catalyst remaining in the polymer product, as identified by ^1^H NMR studies. In addition, TGA studies on the degradation of BO*-alt*-PA catalyzed by air-free ChCl (*T*_d,5%_ = 315 °C), ChCl/urea, (*T*_d,5%_ = 305 °C), and ChCl/EG (*T*_d,5%_ = 312 °C) aligned with the literature value (*T*_d,5%_ = 309 °C) reported previously.^68^ Low toxicity also suggests that even if residual catalyst remains in the polymer, it would not be problematic for use in applications that could impact human health. Attempts to leave the catalyst in the polymer were unsuccessful, as all routes to precipitate out the polymer showed no remaining choline in the ^1^H NMR spectrum. These results contrast those from MALDI-TOF-MS studies, which indicated the possible initiation from the choline alcohol. More detailed mechanistic studies, both experimental and theoretical, will be needed for the most optimal air-free DES catalysts to better understand this polymerization route.

### Comparison to Literature Organocatalysts

Organocatalysts have not been able to compete with metal-based catalysts for the ROCOP of epoxides and cyclic anhydrides, as the focus is to get the best activity and selectivity. The leading catalysts are therefore often air-sensitive with more complex synthetic methods. In Figure 1A, the Al(III)/K(I) catalyst used for the ROCOP of CHO and PA from Williams et al. reached a TOF of 1072 h^–1^ in 15 min at 50 °C with good selectivity and dispersity control.^24^ Coates et al. have also investigated the ROCOP of a range of epoxides and cyclic anhydrides using an aluminum catalyst with a salen ligand tethered to a cyclopropenium cocatalyst. They explored the polymerization of PO and PA, which had a TOF = >100 h^–1^ in 6 h at 60 °C.^25^

In our previous work using yttrium simple salts with the [PPN]Cl cocatalyst, the monomer pair CHO/PA had a TOF of 85 h^–1^ in 50 min at 110 °C (Figure 1B). However, a closer comparison with the monomers explored in this study is BO/CPMA, which had a TOF of 402 h^–1^ in 10 min at 110 °C.^29^ In addition, Satoh et al. found that potassium acetate salts are active for the ROCOP of PA and ethyl glycidyl ether (TOF = 42.5 h^–1^) but moved forward in the direction of cesium pivalate salts (TOF = 68.6 h^–1^), both in 1 h at 100 °C, as the rate and control of polymerization were prioritized.^34^

However, the leading metal catalysts are often air-sensitive with more complex synthetic methods, and the toxicity of coordination complexes is often more complex than organic catalysts. In this context, if the rate of polymerization is the goal, an organocatalyst is not currently the first choice. DES catalysts prioritize metal-free simple catalyst synthesis and low toxicity in which organocatalysts are more likely to offer similar attributes. The monomers selected in this study represent the broad range of available epoxides and cyclic anhydrides, with consideration of the epoxide boiling point (which required the use of 1-butene oxide over propylene oxide). With different catalysts, monomers, loadings, and temperatures in the literature, it is difficult to make direct comparisons. However, one of the most common monomer pairs in the literature is CHO and PA, with organocatalysts able to polymerize this monomer pair at TOFs up to 456 h^–1^ in similar conditions.^43,51−54^ While the reported DES catalysts can only reach TOFs of up to 198 h^–1^, these catalysts have low toxicity and require only a facile synthetic preparation in neat conditions.^55,58−60^ Most organocatalysts in the literature include toxic reagents and/or require multistep synthesis of the catalyst. Additionally, these catalysts were able to reach a moderate TOF for six different monomer pairs, with many organocatalysts only being reported for a specific monomer scope.^32,43,51−54,61^ Therefore, these DES catalysts, which are biocompatible,^55−58^ controlled from side reactions, and moderate in polymerization rate and only require facile synthetic steps in neat conditions, represent a sustainable advance.

## Conclusions

The ROCOP of epoxides and cyclic anhydrides is a direction toward more diverse polymer structures that can offer a more biofriendly and biodegradable alternative to current commercial plastics. The ideal catalyst for this process has low toxicity, is cost-effective, and is environmentally friendly, which expands its range of applications. Choline halide salts and DESs have been identified as active catalysts for the ROCOP of BO or CHO and CPMA, PA, or GA, although the halides did not significantly affect the initiation of polymerization.

With a focus on BO/CPMA monomers, due to their ease of purification from water and diacids, and ChCl/urea, as it is the most commonly studied DES, it was found that the air-exposed polymerizations maintained low dispersity but produced lower molar masses than expected. This can be attributed to water likely acting as a chain transfer agent. On the other hand, air-free polymerizations had consistently closer molar masses to their theoretical molar mass without sacrificing TOF but with the consequence of the dispersity increasing. This suggests that while the DESs do not act as a chain transfer agent, they likely provide hydrogen bonding similar to that of water, increasing the polymerization rate.

The use of choline halide salts and DESs decreases the worry of the catalyst remaining in the polymer, which also lessens its concern for biomedical use. We hope that these results encourage the exploration of more biocompatible catalysts for the synthesis of polyesters through the ring-opening copolymerization of epoxides and cyclic anhydrides and other methods.

## Supporting Information Available

General considerations for instruments, chemicals, monomers, and solvents, DES synthetic methods and polymerization methods, conditions, additional tabulated polymerization replicate results, ^1^H NMR of selected polymers, GPC curves of selected polymers, and TGA curves of selected polymers and air-free and air-exposed ChCl, ChCl/urea, and ChCl/EG (PDF)
