# Supplementary material for: Choline Halide-Based Deep Eutectic Solvents as Biocompatible Catalysts for the Alternating Copolymerization of Epoxides and Cyclic Anhydrides
Source: ACS Sustain Chem Eng. 2024 Apr 30;12(19):7246–55. doi: 10.1021/acssuschemeng.3c06766 (PMC11094800; doi:10.1021/acssuschemeng.3c06766)
Supplement: Supplementary file 1 — sc3c06766_si_001.pdf [file sc3c06766_si_001.pdf]

Supporting Information for

Choline Halide-Based Deep Eutectic Solvents as Biocompatible Catalysts for the Alternating Copolymerization of Epoxides and Cyclic Anhydrides

Mary Dana Czarinah L. Cheng-Tan,[‡a] Angelyn N. Nguyen,[‡a] Collette T. Gordon,[a] Zachary A. Wood,[a] Yvonne Manjarrez[a] and Megan E. Fieser[a,b]*

[‡]Authors contributed equally

*Corresponding author; Email: fieser@usc.edu

[a]*Department of Chemistry, University of Southern California, Los Angeles, CA 90089*

[b]*Wrigley Institute for Environment and Sustainability, University of Southern California, Los Angeles, CA 90089*

Number of Pages: 95

Number of Figures: 78

Number of Tables: 20

**1.**	**General considerations**	**S4**
**2.**	**General synthesis and polymerization**	**S6**
**3.**	**Tabulated polymerization data**	**S9**
	**3.1 Air-exposed polymerization data**	**S9**
	**Table S1.** Replicate catalytic reactions with ChCl as the catalyst	S9
	**Table S2.** Replicate catalytic reactions with ChBr as the catalyst	S10
	**Table S3.** Replicate catalytic reactions with ChI as the catalyst	S11
	**Table S4.** Replicate catalytic reactions with ChCl/urea DES as the catalyst	S12
	**Table S5.** Replicate catalytic reactions with ChCl/EG DES as the catalyst	S13
	**Table S6.** Replicate catalytic reactions with ChI/urea DES as the catalyst	S14
	**Table S7.** Replicate catalytic reactions with ChI/EG DES as the catalyst	S15
	**Table S8.** Replicate catalytic reactions with ChBr/urea DES as the catalyst	S16
	**Table S9.** Replicate catalytic reactions with ChBr/EG DES as the catalyst	S17
	**3.2 Air-free polymerization data**	**S18**
	**Table S10.** Replicate catalytic reactions with ChCl as the catalyst	S18
	**Table S11.** Replicate catalytic reactions with ChBr as the catalyst	S19

Table S12. Replicate catalytic reactions with ChI as the catalyst S20
Table S13. Replicate catalytic reactions with ChCl/urea DES as the catalyst S21
Table S14. Replicate catalytic reactions with ChCl/EG DES as the catalyst S22
Table S15. Replicate catalytic reactions with ChI/urea DES as the catalyst S23
Table S16. Replicate catalytic reactions with ChI/EG DES as the catalyst S24
Table S17. Replicate catalytic reactions with ChBr/urea DES as the catalyst S25
Table S18. Replicate catalytic reactions with ChBr/EG DES as the catalyst S26
4. ^{1}H NMR spectra S27
Figure S1. ^{1}H NMR spectrum of *in-situ* BO-*alt*-CPMA in $CDCl_3$ (**Table S1**, entry 1b) S27
Figure S2. ^{1}H NMR spectrum of isolated BO-*alt*-GA in $CDCl_3$ (**Table S1**, entry 3a) S28
Figure S3. ^{1}H NMR spectrum of *in-situ* CHO-*alt*-PA in $CDCl_3$ (**Table S1**, entry 5a) S29
Figure S4. ^{1}H NMR spectrum of *in-situ* CHO-*alt*-PA in $CDCl_3$ (**Table S1**, entry 5b) S30
Figure S5. ^{1}H NMR spectrum of isolated BO-*alt*-CPMA in $CDCl_3$ (**Table S2**, entry 1a) S31
Figure S6. ^{1}H NMR spectrum of isolated BO-*alt*-GA in $CDCl_3$ (**Table S2**, entry 3a) S32
Figure S7. ^{1}H NMR spectrum of *in-situ* CHO-*alt*-PA in $CDCl_3$ (**Table S2**, entry 5b) S33
Figure S8. ^{1}H NMR spectrum of isolated BO-*alt*-CPMA in $CDCl_3$ (**Table S3**, entry 1a) S34
Figure S9. ^{1}H NMR spectrum of *in-situ* CHO-*alt*-CPMA in $CDCl_3$ (**Table S3**, entry 4a) S35
Figure S10. ^{1}H NMR spectrum of isolated CHO-*alt*-GA in $CDCl_3$ (**Table S3**, entry 6a) S36
Figure S11. ^{1}H NMR spectrum of *in-situ* CHO-*alt*-GA in $CDCl_3$ (**Table S3**, entry 6b) S37
Figure S12. ^{1}H NMR spectrum of *in-situ* BO-*alt*-CPMA in $CDCl_3$ (**Table S4**, entry 1a) S38
Figure S13. ^{1}H NMR spectrum of *in-situ* BO-*alt*-PA in $CDCl_3$ (**Table S4**, entry 2c) S39
Figure S14. ^{1}H NMR spectrum of isolated CHO-*alt*-CPMA in $CDCl_3$ (**Table S4**, entry 4b) S40
Figure S15. ^{1}H NMR spectrum of *in-situ* BO-*alt*-CPMA in $CDCl_3$ (**Table S5**, entry 1a) S41
Figure S16. ^{1}H NMR spectrum of *in-situ* BO-*alt*-PA in $CDCl_3$ (**Table S5**, entry 2a) S42
Figure S17. ^{1}H NMR spectrum of *in-situ* CHO-*alt*-PA in $CDCl_3$ (**Table S5**, entry 5a) S43
Figure S18. ^{1}H NMR spectrum of *in-situ* BO-*alt*-CPMA in $CDCl_3$ (**Table S6**, entry 1b) S44
Figure S19. ^{1}H NMR spectrum of *in-situ* CHO-*alt*-CPMA in $CDCl_3$ (**Table S6**, entry 4a) S45
Figure S20. ^{1}H NMR spectrum of -*in-situ* BO-*alt*-CPMA in $CDCl_3$ (**Table S7**, entry 1a) S46
Figure S21. ^{1}H NMR spectrum of *in-situ* BO-*alt*-GA in $CDCl_3$ (**Table S7**, entry 3a) S47
Figure S22. ^{1}H NMR spectrum of *in-situ* BO-*alt*-GA in $CDCl_3$ (**Table S7**, entry 3c) S48
Figure S23. ^{1}H NMR spectrum of *in-situ* BO-*alt*-PA in $CDCl_3$ (**Table S8**, entry 2b) S49
Figure S24. ^{1}H NMR spectrum of isolated CHO-*alt*-PA in $CDCl_3$ (**Table S8**, entry 5a) S50
Figure S25. ^{1}H NMR spectrum of *in-situ* CHO-*alt*-CPMA in $CDCl_3$ (**Table S9**, entry 4a) S51
Figure S26. ^{1}H NMR spectrum of *in-situ* BO-*alt*-CPMA in $CDCl_3$ (**Table S10**, entry 1a) S52
Figure S27. ^{1}H NMR spectrum of isolated BO-*alt*-CPMA in $CDCl_3$ (**Table S10**, entry 2b) S53
Figure S28. ^{1}H NMR spectrum of isolated BO-*alt*-GA in $CDCl_3$ (**Table S10**, entry 3b) S54
Figure S29. ^{1}H NMR spectrum of *in-situ* CHO-*alt*-PA in $CDCl_3$ (**Table S10**, entry 5b) S55
Figure S30. ^{1}H NMR spectrum of isolated BO-*alt*-CPMA in $CDCl_3$ (**Table S11**, entry 1a) S56
Figure S31. ^{1}H NMR spectrum of isolated CHO-*alt*-GA in $CDCl_3$ (**Table S11**, entry 6c) S57
Figure S32. ^{1}H NMR spectrum of isolated BO-*alt*-CPMA in $CDCl_3$ (**Table S12**, entry 1a) S58
Figure S33. ^{1}H NMR spectrum of isolated CHO-*alt*-GA in $CDCl_3$ (**Table S12**, entry 6a) S59
Figure S34. ^{1}H NMR spectrum of isolated BO-*alt*-CPMA in $CDCl_3$ (**Table S13**, entry 1b) S60
Figure S35. ^{1}H NMR spectrum of isolated BO-*alt*-CPMA in $CDCl_3$ (**Table S13**, entry 1e) S61
Figure S36. ^{1}H NMR spectrum of *in-situ* BO-*alt*-PA in $CDCl_3$ (**Table S13**, entry 2b) S62

Figure S37. ^{1}H NMR spectrum of *in-situ* CHO-*alt*-CPMA in $CDCl_3$ (**Table S13**, entry 4a) S63
Figure S38. ^{1}H NMR spectrum of *in-situ* CHO-*alt*-PA in $CDCl_3$ (**Table S13**, entry 5b) S64
Figure S39. ^{1}H NMR spectrum of isolated CHO-*alt*-CPMA in $CDCl_3$ (**Table S14**, entry 4a) S65
Figure S40. ^{1}H NMR spectrum of isolated CHO-*alt*-PA in $CDCl_3$ (**Table S14**, entry 5c) S66
Figure S41. ^{1}H NMR spectrum of isolated BO-*alt*-PA in $CDCl_3$ (**Table S16**, entry 2c) S67
5. GPC spectra S68
Figure S42. GPC trace of **Table S1**, entry 1b and **Table 2**, entry 1 (LS on the left, RI on the right). BO-*alt*-CPMA from ChCl exposed to air S68
Figure S43. GPC trace of **Table S1**, entry 2a (LS on the left, RI on the right). BO-*alt*-PA from ChCl exposed to air S68
Figure S44. GPC trace of **Table S2**, entry 1a (LS on the left, RI on the right). BO-*alt*-CPMA from ChBr exposed to air S69
Figure S45. GPC trace of **Table S2**, entry 3a (LS on the left, RI on the right). BO-*alt*-GA from ChBr exposed to air S69
Figure S46. GPC trace of **Table S3**, entry 1a (LS on the left, RI on the right). BO-*alt*-CPMA from ChI exposed to air S70
Figure S47. GPC trace of **Table S3**, entry 4a (LS on the left, RI on the right). CHO-*alt*-CPMA from ChI exposed to air S70
Figure S48. GPC trace of **Table S4**, entry 1c and **Table 2**, entry 2 (LS on the left, RI on the right). BO-*alt*-CPMA from ChCl:urea exposed to air S71
Figure S49. GPC trace of **Table S5**, entry 1a and **Table 2**, entry 3 (LS on the left, RI on the right). BO-*alt*-CPMA from ChCl:EG exposed to air S71
Figure S50. GPC trace of **Table S5**, entry 2a (LS on the left, RI on the right). BO-*alt*-PA from ChCl/EG exposed to air S72
Figure S51. GPC trace of **Table S7**, entry 1a (LS on the left, RI on the right). BO-*alt*-CPMA from ChBr/EG exposed to air S72
Figure S52. GPC trace of **Table S8**, entry 5a (LS on the left, RI on the right). CHO-*alt*-PA from ChI/urea exposed to air S73
Figure S53. GPC trace of **Table S9**, entry 1a (LS on the left, RI on the right). BO-*alt*-CPMA from ChI/EG exposed to air S73
Figure S54. GPC trace of **Table S9**, entry 4a (LS on the left, RI on the right). CHO-*alt*-CPMA from ChI/EG exposed to air S74
Figure S55. GPC trace of **Table S10**, entry 1d and **Table 2**, entry 4 (LS on the left, RI on the right). BO-*alt*-CPMA from ChCl air-free S74
Figure S56. GPC trace of **Table S13**, entry 1b and **Table 2**, entry 5 (LS on the left, RI on the right). BO-*alt*-CPMA from ChCl/urea air-free S75
Figure S57. GPC trace of **Table S14**, entry 1b and **Table 2**, entry 6 (LS on the left, RI on the right). BO-*alt*-CPMA from ChCl/EG air-free S75
6. TGA data S76
6.1 TGA spectra for the degradation of polymers S76
Figure S58. TGA spectrum of BO-*alt*-PA (**Table S10**, entry 2f) using air-free ChCl heated from 25 °C – 550 °C. S76
Figure S59. TGA spectrum of BO-*alt*-PA (**Table S13**, entry 2d) using air-free ChCl/urea heated from 25 °C – 550 °C. S76
Figure S60. TGA spectrum of BO-alt-PA (**Table S14**, entry 2d) using air-free ChCl/EG heated from 25 °C – 550 °C. S77
6.2 TGA spectra of catalysts to determine its water content S78
Figure S61. TGA spectrum of air-exposed ChCl heated from 25 °C – 500 °C S78

Figure S62. TGA spectrum of air-exposed ChCl/urea heated from 25 °C – 500 °C S79
Figure S63. TGA spectrum of air-exposed ChCl/EG heated from 25 °C – 500 °C S79
Figure S64. TGA spectrum of air-free ChCl heated from 25 °C – 500 °C S80
Figure S65. TGA spectrum of air-free ChCl/urea heated from 25 °C – 500 °C S80
Figure S66. TGA spectrum of air-free ChCl/EG heated from 25 °C – 500 °C S81
6.3 TGA spectra of EG S81
Figure S67. TGA spectrum of air-exposed EG heated from 25 °C – 500 °C S81
7. MALDI-TOF-MS data S82
Table S19. Repeat Unit MW and End-Group MW Average via MALDI-TOF-MS Analysis using DCTB as the Matrix S82
Table S20. Repeat Unit MW and End-Group MW Average via MALDI-TOF-MS Analysis using DHB as the Matrix S83
7.1 DCTB matrix S84
Figure S68. MALDI-TOF spectrum of **Table S1**, entry 1c and **Table 2,** entry 1. BO-*alt*-CPMA from ChCl exposed to air S84
Figure S69. MALDI-TOF spectrum of **Table S4**, entry 1c and **Table 2** entry 2. BO-*alt*-CPMA from ChCl/urea exposed to air S85
Figure S70. MALDI-TOF spectrum of **Table S5**, entry 1a and **Table 2** entry 3. BO-*alt*-CPMA from ChCl/EG exposed to air S86
Figure S71. MALDI-TOF spectrum of **Table S10**, entry 1d and **Table 2,** entry 4. BO-*alt*-CPMA from ChCl air-free S87
Figure S72. MALDI-TOF spectrum of **Table S13**, entry 1b and **Table 2** entry 5. BO-*alt*-CPMA from ChCl/urea air-free S88
Figure S73. MALDI-TOF spectrum of **Table S14**, entry 1b and **Table 2** entry 6. BO-*alt*-CPMA from ChCl/EG air-free S89
7.2 DHB matrix S90
Figure S74. MALDI-TOF spectrum of **Table S1** entry 1c and **Table 2** entry 1. BO-al*t*-CPMA from ChCl exposed to air S90
Figure S75. MALDI-TOF spectrum of **Table S4** entry 1c and **Table 2** entry 2. BO-al*t*-CPMA from ChCl/urea exposed to air S91
Figure S76. MALDI-TOF spectrum of **Table S5** entry 1a and **Table 2** entry 3. BO-*alt*-CPMA from ChCl/EG exposed to air S92
Figure S77. MALDI-TOF spectrum of **Table S10** entry 1d and **Table 2** entry 4. BO-*alt*-CPMA from ChCl air-free S93
Figure S78. MALDI-TOF spectrum of **Table S10** entry 1d and **Table 2** entry 4 in reflector mode. BO-*alt*-CPMA from ChCl air-free S94
8. References S95

1. General Considerations

Syntheses and manipulations that were carried out under a nitrogen atmosphere are noted and conducted in an OMNI-LAB – Vacuum Atmospheres Company glovebox or by using standard Schlenk line techniques. ^{1}H spectra were recorded on a Varian Mercury 400 2-Channel NMR Spectrometer. Chemical shifts (δ) for the ^{1}H NMR spectra were referenced to the residual chloroform (7.26 ppm), signal. Polymer molar masses and dispersities were determined by Gel Permeation Chromatography (GPC) instrument, equipped with an Agilent 1260 Infinity II HPLC System and autosampler, two Agilent PolyPore columns (5 micron, 4.6 mm ID) in series unless otherwise noted, a Wyatt DAWN HELEOS-II light scattering detector, and a Wyatt Optilab T-rEX refractive index detector. The columns were eluted with HPLC grade THF at 30 °C at a flow rate of 0.3 mL/min, and polymer samples were dissolved in this solvent and filtered through a 0.2 micron PTFE membrane before SEC- MALS (size exclusion chromatography-multi-angle light scattering) analyses. dn/dc values were calculated from the RI signal by using the 100% mass recovery method in the Astra software and a known sample concentration.[1] Thermal gravimetric analysis (TGA) data were collected using a Mettler-Toledo STARe System TGA/DSC3+ equipped with STARe software, a TA SDTA Sensor LF, XP1 Balance, and a sample robot. Sample weight of the samples were between 6 – 10 mg and sealed in a 40 μL aluminum crucible fitted with a pierceable lid. To determine the degradation of polymers, the samples were heated from 25 °C – 550 °C at a scan rate of 10 °C/min under a constant N_2 flow of 15 mL/min. To determine the catalysts' water content involved heating the samples from 25 °C – 100 °C, holding it at 100 °C for 8 h, then heating to 500 °C at a scan rate of 10 °C/min under a constant N_2 flow of 15 mL/min. Matrix assisted laser desorption ionization time-of-flight mass spectrometry (MALDI-TOF-MS) analyses were performed by Mass Spectrometry Lab of the School of Chemical Sciences at the University of Illinois, Urbana Champaign on a Bruker Autoflex Speed LRF instrument.

Phthalic anhydride (PA) 99% from ACROS Organics was sublimed at 100 °C under static vacuum, dried for 36 h at 10^{-3} Torr, and stored in the glovebox. Glutaric anhydride (GA) 98% from Beantown Chemical was stored under inert atmosphere and sublimed at 58 °C under static vacuum before use. Carbic anhydride (CPMA) >99% from ACROS Organics was recrystallized with 1:4 ethyl acetate:hexanes, the crystals were dried for 36 h at 100 °C at 10^{-3} Torr and stored in the glovebox. 1-butene oxide (BO) and cyclohexene oxide (CHO) were dried over CaH_2 for 5 d under inert atmosphere, degassed by the freeze-pump-thaw method three times, vacuum transferred, and stored in the glovebox. Choline (2-hydroxy-N,N,N,-triethylethanaminium) chloride (ChCl) 98% from Beantown Chemical was was used as received or dried at 100 °C at 10^{-3} Torr for 5 d, and stored in the glovebox. Choline iodide (ChI) >98% from TCI, choline bromide (ChBr) >98% from TCI, and urea >99.5% from Sigma Aldrich were used as received or dried at 100 °C at 10^{-3} Torr for 36 h and stored in the glovebox. Ethylene glycol (EG) anhydrous 99.8% from Sigma Aldrich was stored in the glovebox.

2. General Synthesis and Polymerization

2.1 General synthesis of air-exposed deep eutectic solvents

In a round bottom flask equipped with a Teflon coated stir bar, 1 eq. of choline-halogen (ChX, X = Cl, I, Br) was added to 2 eq. of EG or urea, heated and stirred overnight or until homogeneous and colorless. ChI/urea, ChBr/urea, ChI/EG, and ChBr/EG mixtures were heated to 110 °C. ChCl/urea and ChCl/EG were heated to 50°C. ChI/urea and ChI/EG solidified to a white solid upon cooling to rt. The remaining 4 DESs remained liquid. These were stored in atmospheric conditions.

ChCl/Urea: ChCl (0.9000 g, 6.4 mmol, 1.0 equiv.); Urea (0.0774 g, 12.8 mmol, 2.0 equiv.)

ChCl/EG: ChCl (0.9152 g, 6.6 mmol, 1.0 equiv.); EG (1.016 g, 16.4 mmol, 2.48 equiv.)

ChBr/Urea: ChBr (0.1006 g, 0.55 mmol, 1.0 equiv.); Urea (0.065 g, 1.09 mmol, 1.98 equiv.)

ChBr/EG: ChBr (0.1013 g, 0.55 mmol, 1.0 equiv.); EG (0.0671 g, 1.08 mmol, 2.0 equiv.)

ChI/Urea: ChI (0.1005 g, 0.43 mmol, 1.0 equiv.); Urea (0.0515 g, 0.87 mmol, 2.0 equiv.)

ChI/EG: ChI (0.1024 g, 0.44 mmol, 1.0 equiv.); EG (0.0539 g, 0.87 mmol, 2.0 equiv.)

2.2. General synthesis of air-free deep eutectic solvents

Using analogous methods to the benchtop synthesis, DESs were synthesized using anhydrous ChX (X= Cl, I, Br), urea, and EG in a glovebox atmosphere. The mixtures of ChI and ChBr with urea solidified to a white powder upon cooling to rt, while the mixture of ChCl/urea solidified to a white waxy solid. The mixtures of ChX/EG remained a colorless liquid upon cooling to rt. These were stored in the glovebox.

ChCl/Urea: ChCl (0.4025 g, 2.8 mmol, 1.0 equiv.); Urea (0.3485 g, 5.8 mmol, 2.1 equiv.)

ChCl/EG: ChCl (0.4113 g, 2.9 mmol, 1.0 equiv.); EG (0.3556 g, 5.7 mmol, 2.0 equiv.)

ChBr/Urea: ChBr (0.2048 g, 1.1 mmol, 1.0 equiv.); Urea (0.1346 g, 2.2 mmol, 2.0 equiv.)

ChBr/EG: ChBr (0.2165 g, 1.2 mmol, 1.0 equiv.); EG (0.1449 g, 2.3 mmol, 1.9 equiv.)

ChI/Urea: ChI (0.2140 g, 0.93 mmol, 1.0 equiv.); Urea (0.1102 g, 1.8 mmol, 1.9 equiv.)

ChI/EG: ChI (0.2097 g, 0.91 mmol, 1.0 equiv.); EG (0.1124 g, 1.8 mmol, 2.0 equiv.)

2.3. General polymerization

Catalyst, anhydride, epoxide and a stir bar were charged into a vial equipped with a Teflon-lined cap and stir bar. [1]:[100]:[500] molar ratio of [DES]:[anhydride]:[epoxide] was used. The vial was sealed with electrical tape and put on a metal screening block that was preheated to 110 °C for at least 1 h. Reactions with CPMA ran for 80 min, PA for 30 min, and GA for 60 min unless otherwise noted. The vial was cooled to room temperature and the resulting mixture was quenched with 1 mL of chloroform. Hexanes were added in excess until the polymer started to precipitate. The precipitate was allowed to settle, and the supernatant was decanted. The isolated polymer was dried under reduced pressure at 50–65 °C overnight. Anhydride conversions and percent esters were calculated via analysis of the *in-situ* ^{1}H NMR spectra in $CDCl_3$. Percent epimerizations were calculated via analysis of the isolated ^{1}H NMR spectra in $CDCl_3$ molar masses and dispersities were calculated via GPC, as described above.

2.31 Air-exposed polymerization

The same procedure as the general polymerization using air-exposed DESs as the catalyst with purified and dried monomers (CPMA, PA, GA, BO, CHO) that were stored in atmospheric conditions.

2.32 Air-free polymerization

The same procedure as the general polymerization but used air-free DESs as the catalyst with monomers (CPMA, PA, GA, BO, CHO) that were purified, dried, and stored in the glovebox. The reaction mixture was made in a glovebox, and the mixture was not exposed to air until the polymerization was quenched.

3. Tabulated Polymerization Data

3.1 Air-exposed polymerizations

Table S1. Replicate catalytic reactions for the copolymerization of epoxides and cyclic anhydrides with ChCl as the catalyst.[a]

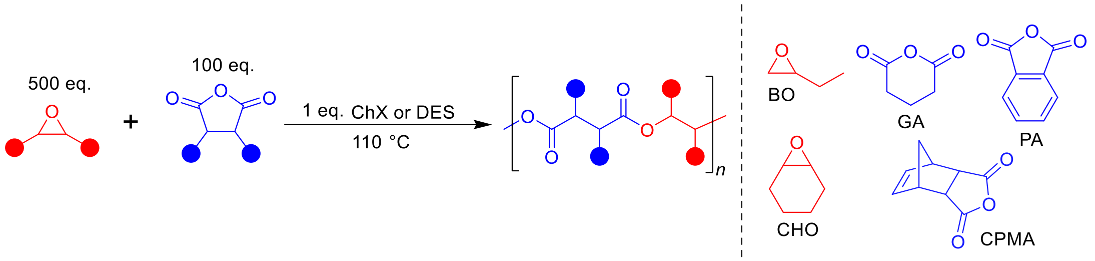

entry	anhydride	epoxide	conv.[b] (%)	TOF[c] (h^{-1})	ester sel.[d] (%)	epimer.[e] (%)	$M_{nt,heo}$[f] (kDa)	$M_{n,exp}$[g] (kDa)	$Đ$[g]
1a	CPMA	BO	58	44	88	7	14.3	3.6	1.2
1b	CPMA		39	29	91	-	9.5	2.9	1.1
1c	CPMA		52	39	85	18	12.9	1.3	1.1
1d	CPMA		72	54	91	8	17.5	9.6	1.8
2a	PA		>99	171	>99	-	22.1	3.4	1.0
2b	PA		90	180	>99	-	-	-	-
2c	PA		78	156	>99	-	-	-	-
3a	GA		63	63	>99	-	-	-	-
3b	GA		63	63	89	-	-	-	-
3c	GA		71	71	>99	-	-	-	-
4a	CPMA	CHO	80	60	>99	21	-	-	-
4b	CPMA		87	65	>99	-	-	-	-
4c	CPMA		64	48	>99	7	18.6	1.4	1.4
5a	PA		91	182	75	-	25.4	1.9	1.0
5b	PA		95	190	96	-	-	-	-
5c	PA		95	190	83	-	-	-	-
6a	GA		76	76	>99	-	-	-	-
6b	GA		70	70	65	-	-	-	-
6c	GA		47	47	>99	-	-	-	-

[a][catalyst]:[anhydride]:[epoxide] was 1:100:500. Reactions were heated to 110 °C under neat conditions. The polymerizations with CPMA ran for 80 min, PA for 30 min, and GA for 60 min. [b]Determined using ^1H NMR spectra of crude reaction mixtures, comparing the conversion of anhydride monomers to polymers. [c]Defined as mol anhydride consumed/(mol catalyst) x h. [d]Ester selectivity was determined by using the ^1H NMR spectra of *in-situ* polymers, comparing the polyether signal to a polyester signal. [e]Determined using ^1H NMR spectra of purified polymers with % epimer. = $\{2 \times A_{2.7\ ppm}/(A_{6.0\text{-}6.5\ ppm})\} \times 100$. [f]Calculated for 1 chloride initiator. [g]Identified by GPC, using a Wyatt MALS detector.

Table S2. Replicate catalytic reactions for the copolymerization of epoxides and cyclic anhydrides with ChBr as the catalyst.[a]

entry	anhydride	epoxide	conv.[b] (%)	TOF[c] (h^{-1})	ester sel.[d] (%)	epimer.[e] (%)	$M_{n,theo}$[f] (kDa)	$M_{n,exp}$[g] (kDa)	$Đ$[g]
1a	CPMA	BO	77	58	86	8	19.1	4.4	1.4
1b	CPMA		79	59	>99	-	-	-	-
1c	CPMA		74	56	>99	-	-	-	-
2a	PA		98	49	>99	-	21.6	4.8	1.2
2b	PA		93	47	>99	-	-	-	-
2c	PA		>99	50	>99	-	-	-	-
3a	GA		86	86	>99	-	15.9	1.8	1.3
3b	GA		66	66	98	-	-	-	-
3c	GA		90	90	90	-	-	-	-
4a	CPMA	CHO	69	52	87	11	19.1	3.2	1.5
4b	CPMA		64	48	>99	-	-	-	-
4c	CPMA		64	48	>99	-	-	-	-
5a	PA		96	192	88	-	24.9	2.6	1.3
5b	PA		>99	198	88	-	-	-	-
5c	PA		>99	198	79	-	-	-	-
6a	GA		64	64	>99	-	13.6	1.4	1.2
6b	GA		60	60	75	-	-	-	-
6c	GA		65	65	72	-	-	-	-

[a][catalyst]:[anhydride]:[epoxide] was 1:100:500. Reactions were heated to 110 °C under neat conditions. The polymerizations with CPMA ran for 80 min, PA for 30 min, and GA for 60 min. [b]Determined using ^{1}H NMR spectra of crude reaction mixtures, comparing the conversion of anhydride monomers to polymers. [c]Defined as mol anhydride consumed/(mol catalyst) x h. [d]Ester selectivity was determined by using the ^{1}H NMR spectra of *in-situ* polymers, comparing the polyether signal to a polyester signal. [e]Determined using ^{1}H NMR spectra of purified polymers with % epimer. = $\{2 \times A_{2.7\ ppm}/(A_{6.0\text{-}6.5\ ppm})\} \times 100$. [f]Calculated for 1 chloride initiator. [g]Identified by GPC, using a Wyatt MALS detector.

Table S3. Replicate catalytic reactions for the copolymerization of epoxides and cyclic anhydrides with ChI as the catalyst.[a]

entry	anhydride	epoxide	conv.[b] (%)	TOF[c] (h^{-1})	ester sel.[d] (%)	epimer.[e] (%)	$M_{n,theo}$[f] (kDa)	$M_{n,exp}$[g] (kDa)	$Đ$[g]
1a	CPMA	BO	97	73	>99	32	23.0	4.9	1.8
1b	CPMA		81	61	>99	-	-	-	-
1c	CPMA		78	59	>99	-	-	-	-
2a	PA		>99	50	>99	-	21.8	4.8	1.3
2b	PA		>99	50	>99	-	-	-	-
2c	PA		>99	50	>99	-	-	-	-
3a	GA		75	75	>99	-	14.0	1.4	1.4
3b	GA		70	70	>99	-	-	-	-
3c	GA		71	71	>99	-	-	-	-
4a	CPMA	CHO	73	55	89	13	20.0	3.6	1.4
4b	CPMA		79	59	>99	-	-	-	-
4c	CPMA		79	59	>99	-	-	-	-
5a	PA		95	190	>99	-	23.4	2.1	1.4
5b	PA		95	190	80	-	-	-	-
5c	PA		>99	200	79	-	-	-	-
6a	GA		62	62	>99	-	13.1	1.6	3.0
6b	GA		68	68	75	-	-	-	-
6c	GA		70	70	76	-	-	-	-

[a][catalyst]:[anhydride]:[epoxide] was 1:100:500. Reactions were heated to 110 °C under neat conditions. The polymerizations with CPMA ran for 80 min, PA for 30 min, and GA for 60 min. [b]Determined using ^{1}H NMR spectra of crude reaction mixtures, comparing the conversion of anhydride monomers to polymers. [c]Defined as mol anhydride consumed/(mol catalyst) x h. [d]Ester selectivity was determined by using the ^{1}H NMR spectra of *in-situ* polymers, comparing the polyether signal to a polyester signal. [e]Determined using ^{1}H NMR spectra of purified polymers with % epimer. = $\{2 \times A_{2.7\ ppm}/(A_{6.0\text{-}6.5\ ppm})\} \times 100$. [f]Calculated for 1 chloride initiator. [g]Identified by GPC, using a Wyatt MALS detector.

Table S4. Replicate catalytic reactions for the copolymerization of epoxides and cyclic anhydrides with ChCl/urea DES as the catalyst.[a]

entry	anhydride	epoxide	conv.[b] (%)	TOF[c] (h^{-1})	ester sel.[d] (%)	epimer.[e] (%)	$M_{n,theo}$[f] (kDa)	$M_{n,exp}$[g] (kDa)	$Đ$[g]
1a	CPMA	BO	31	23	77	10	8.0	6.1	1.1
1b	CPMA		32	24	56	-	9.4	1.7	1.9
1c	CPMA		72	54	96	11	17.2	3.6	1.3
1d	CPMA		69	52	91	-	16.8	13.7	1.7
1e	CPMA		51	38	93	-	12.3	4.7	1.4
2a	PA		85	171	91	-	19.4	3.0	1.0
2b	PA		66	132	>99	-	-	-	-
2c	PA		96	192	>99	-	-	-	-
3a	GA		46	46	>99	-	8.5	1.5	1.7
3b	GA		79	79	>99	-	-	-	-
3c	GA		64	64	>99	-	-	-	-
4a	CPMA	CHO	57	43	>99	-	-	-	-
4b	CPMA		73	55	93	6	-	-	-
4c	CPMA		69	52	>99	-	-	-	-
5a	PA		>99	198	81	-	-	-	-
5b	PA		>99	198	76	-	-	-	-
5c	PA		>99	198	78	-	-	-	-
6a	GA		60	60	70	-	-	-	-
6b	GA		76	76	66	-	-	-	-
6c	GA		80	80	74	-	-	-	-

[a][catalyst]:[anhydride]:[epoxide] was 1:100:500. Reactions were heated to 110 °C under neat conditions. The polymerizations with CPMA ran for 80 min, PA for 30 min, and GA for 60 min. [b]Determined using ^1H NMR spectra of crude reaction mixtures, comparing the conversion of anhydride monomers to polymers. [c]Defined as mol anhydride consumed/(mol catalyst) x h. [d]Ester selectivity was determined by using the ^1H NMR spectra of *in-situ* polymers, comparing the polyether signal to a polyester signal. [e]Determined using ^1H NMR spectra of purified polymers with % epimer. = $\{2 \times A_{2.7\ ppm}/(A_{6.0\text{-}6.5\ ppm})\} \times 100$. [f]Calculated for 1 chloride initiator. [g]Identified by GPC, using a Wyatt MALS detector.

Table S5. Replicate catalytic reactions for the copolymerization of epoxides and cyclic anhydrides with ChCl/EG DES as the catalyst.[a]

entry	anhydride	epoxide	conv.[b] (%)	TOF[c] (h^{-1})	ester sel.[d] (%)	epimer.[e] (%)	$M_{n,theo}$[f] (kDa)	$M_{n,exp}$[g] (kDa)	$Đ$[g]
1a	CPMA	BO	89	67	>99	5	21.0	1.9	1.0
1b	CPMA		88	66	57	-	25.6	3.3	1.1
1c	CPMA		81	61	91	7	19.7	4.0	1.3
2a	PA		93	187	87	-	21.4	2.1	1.0
2b	PA		98	196	>99	-	21.7	3.4	1.2
2c	PA		>99	198	>99	-	-	-	-
2d	PA		>99	198	>99	-	-	-	-
3a	GA		76	76	>99	-	14.2	1.4	1.2
3b	GA		70	70	>99	-	-	-	-
3c	GA		62	62	92	-	-	-	-
3d	GA		81	81	97	-	-	-	-
3e	GA		76	76	>99	-	-	-	-
4a	CPMA	CHO	79	59	>99	-	-	-	-
4b	CPMA		70	53	>99	-	-	-	-
4c	CPMA		76	57	>99	8	-	-	-
5a	PA		>99	198	84	-	-	-	-
5b	PA		>99	198	73	-	-	-	-
5c	PA		>99	198	80	-	-	-	-
6a	GA		61	61	72	-	-	-	-
6b	GA		68	68	71	-	-	-	-
6c	GA		66	66	72	-	-	-	-
6d	GA		69	69	79	-	-	-	-

[a][catalyst]:[anhydride]:[epoxide] was 1:100:500. Reactions were heated to 110 °C under neat conditions. The polymerizations with CPMA ran for 80 min, PA for 30 min, and GA for 60 min. [b]Determined using ^{1}H NMR spectra of crude reaction mixtures, comparing the conversion of anhydride monomers to polymers. [c]Defined as mol anhydride consumed/(mol catalyst) x h. [d]Ester selectivity was determined by using the ^{1}H NMR spectra of *in-situ* polymers, comparing the polyether signal to a polyester signal. [e]Determined using ^{1}H NMR spectra of purified polymers with % epimer. = {2 x $A_{2.7\ ppm}$/($A_{6.0\text{-}6.5\ ppm}$)} x 100. [f]Calculated for 1 chloride initiator. [g]Identified by GPC, using a Wyatt MALS detector.

Table S6. Replicate catalytic reactions for the copolymerization of epoxides and cyclic anhydrides with ChBr/urea DES as the catalyst.[a]

entry	anhydride	epoxide	conv.[b] (%)	TOF[c] (h^{-1})	ester sel.[d] (%)	epimer.[e] (%)	$M_{n,theo}$[f] (kDa)	$M_{n,exp}$[g] (kDa)	$Đ$[g]
1a	CPMA	BO	74	56	98	-	-	-	-
1b	CPMA		79	59	93	-	-	-	-
1c	CPMA		84	63	97	14	-	-	-
2a	PA		>99	200	93	-	22.6	4.3	1.2
2b	PA		95	190	>99	-	-	-	-
2c	PA		98	196	93	-	-	-	-
2d	PA		98	196	93	-	-	-	-
3a	GA		75	75	>99	-	-	-	-
3b	GA		70	70	>99	-	-	-	-
3c	GA		84	84	>99	-	-	-	-
4a	CPMA	CHO	64	48	79	6	18.5	2.7	1.1
4b	CPMA		63	47	87	-	-	-	-
4c	CPMA		66	50	86	-	-	-	-
5a	PA		93	186	79	-	25.3	2.3	1.2
5b	PA		95	190	85	-	-	-	-
5c	PA		98	196	76	-	-	-	-
6a	GA		61	61	55	-	17.7	1.1	1.2
6b	GA		70	70	>99	-	-	-	-
6c	GA		72	72	71	-	-	-	-

[a][catalyst]:[anhydride]:[epoxide] was 1:100:500. Reactions were heated to 110 °C under neat conditions. The polymerizations with CPMA ran for 80 min, PA for 30 min, and GA for 60 min. [b]Determined using ^{1}H NMR spectra of crude reaction mixtures, comparing the conversion of anhydride monomers to polymers. [c]Defined as mol anhydride consumed/(mol catalyst) x h. [d]Ester selectivity was determined by using the ^{1}H NMR spectra of *in-situ* polymers, comparing the polyether signal to a polyester signal. [e]Determined using ^{1}H NMR spectra of purified polymers with % epimer. = {2 x $A_{2.7\ ppm}$/($A_{6.0\text{-}6.5\ ppm}$)} x 100. [f]Calculated for 1 chloride initiator. [g]Identified by GPC, using a Wyatt MALS detector.

Table S7. Replicate catalytic reactions for the copolymerization of epoxides and cyclic anhydrides with ChBr/EG DES as the catalyst.[a]

entry	anhydride	epoxide	conv.[b] (%)	TOF[c] (h^{-1})	ester sel.[d] (%)	epimer.[e] (%)	$M_{n,theo}$[f] (kDa)	$M_{n,exp}$[g] (kDa)	*Đ*[g]
1a	CPMA		82	62	95	16	19.4	2.1	1.7
1b	CPMA		75	56	>99	-	-	-	-
1c	CPMA		94	71	>99	-	-	-	-
2a	PA		>99	198	93	-			
2b	PA	BO	96	192	>99	-	-	-	-
2c	PA		64	128	>99	-	-	-	-
3a	GA		74	74	88	-	-	-	-
3b	GA		62	62	98	-	-	-	-
3c	GA		67	67	>99	-	-	-	-
4a	CPMA		80	60	88	4	22.1	3.8	1.1
4b	CPMA		49	37	75	-	-	-	-
4c	CPMA		53	40	96	-	-	-	-
4d	CPMA		>99	74	>99	-	-	-	-
4e	CPMA		73	54	>99	-	-	-	-
5a	PA	CHO	95	190	81	-	25.6	3.1	1.2
5b	PA		>99	198	78	-	-	-	-
5c	PA		>99	198	83	-	-	-	-
6a	GA		67	67	57	-	16.5	4.8	1.4
6b	GA		63	63	71	-	-	-	-
6c	GA		52	52	83	-	-	-	-

[a][catalyst]:[anhydride]:[epoxide] was 1:100:500. Reactions were heated to 110 °C under neat conditions. The polymerizations with CPMA ran for 80 min, PA for 30 min, and GA for 60 min. [b]Determined using ^1H NMR spectra of crude reaction mixtures, comparing the conversion of anhydride monomers to polymers. [c]Defined as mol anhydride consumed/(mol catalyst) x h. [d]Ester selectivity was determined by using the ^1H NMR spectra of *in-situ* polymers, comparing the polyether signal to a polyester signal. [e]Determined using ^1H NMR spectra of purified polymers with % epimer. = {2 x $A_{2.7\ ppm}$/($A_{6.0\text{-}6.5\ ppm}$)} x 100. [f]Calculated for 1 chloride initiator. [g]Identified by GPC, using a Wyatt MALS detector.

Table S8. Replicate catalytic reactions for the copolymerization of epoxides and cyclic anhydrides with ChI/urea DES as the catalyst.[a]

entry	anhydride	epoxide	conv.[b] (%)	TOF[c] (h^{-1})	ester sel.[d] (%)	epimer.[e] (%)	$M_{n,theo}$[f] (kDa)	$M_{n,exp}$[g] (kDa)	Đ[g]
1a	CPMA	BO	80	60	>99	22	-	-	-
1b	CPMA		98	74	>99	-	-	-	-
1c	CPMA		84	63	>99	-	-	-	-
2a	PA		63	126	>99	-			
2b	PA		82	164	>99	-	-	-	-
2c	PA		98	196	93	-	-	-	-
3a	GA		62	62	>99	-			
3b	GA		62	62	98	-	-	-	-
3c	GA		62	62	>99	-	-	-	-
4a	CPMA	CHO	65	49	>99	11	17.0	2.3	1.2
4b	CPMA		55	41	83	-	-	-	-
4c	CPMA		44	33	79	-	-	-	-
5a	PA		95	190	89	-	24.5	2.3	1.1
5b	PA		>99	198	78	-	-	-	-
5c	PA		>99	198	76	-	-	-	-
6a	GA		57	57	99	-	12.1	2.9	1.1
6b	GA		61	61	69	-	-	-	-
6c	GA		69	69	71	-	-	-	-

[a][catalyst]:[anhydride]:[epoxide] was 1:100:500. Reactions were heated to 110 °C under neat conditions. The polymerizations with CPMA ran for 80 min, PA for 30 min, and GA for 60 min. [b]Determined using ^1H NMR spectra of crude reaction mixtures, comparing the conversion of anhydride monomers to polymers. [c]Defined as mol anhydride consumed/(mol catalyst) x h. [d]Ester selectivity was determined by using the ^1H NMR spectra of *in-situ* polymers, comparing the polyether signal to a polyester signal. [e]Determined using ^1H NMR spectra of purified polymers with % epimer. = $\{2 \times A_{2.7\ ppm}/(A_{6.0\text{-}6.5\ ppm})\} \times 100$. [f]Calculated for 1 chloride initiator. [g]Identified by GPC, using a Wyatt MALS detector.

Table S9. Replicate catalytic reactions for the copolymerization of epoxides and cyclic anhydrides with ChI/EG DES as the catalyst.[a]

entry	anhydride	epoxide	conv.[b] (%)	TOF[c] (h^{-1})	ester sel.[d] (%)	epimer.[e] (%)	$M_{n,theo}$[f] (kDa)	$M_{n,exp}$[g] (kDa)	$Đ$[g]
1a	CPMA	BO	85	64	>99	16	20.1	2.5	1.1
1b	CPMA		>99	74	>99	-	-	-	-
1c	CPMA		>99	74	>99	-	-	-	-
2a	PA		>99	198	>99	-	21.8	4.7	3.0
2b	PA		>99	198	98	-	-	-	-
2c	PA		98	196	96	-	-	-	-
3a	GA		76	76	>99	-	14.2	8.5	1.7
3b	GA		65	65	>99	-	-	-	-
3c	GA		65	65	>99	-	-	-	-
4a	CPMA	CHO	69	52	85	10	19.3	4.6	1.4
4b	CPMA		77	58	>99	-	-	-	-
4c	CPMA		73	55	95	-	-	-	-
5a	PA		67	134	80	-	18.1	1.8	1.1
5b	PA		>99	198	79	-	-	-	-
5c	PA		65	130	76	-	-	-	-
5d	PA		>99	198	75	-	-	-	-
5e	PA		86	172	83	-	-	-	-
6a	GA		70	70	>99	-	14.9	2.7	1.0
6b	GA		78	78	78	-	-	-	-
6c	GA		74	74	74	-	-	-	-

[a][catalyst]:[anhydride]:[epoxide] was 1:100:500. Reactions were heated to 110 °C under neat conditions. The polymerizations with CPMA ran for 80 min, PA for 30 min, and GA for 60 min. [b]Determined using ^{1}H NMR spectra of crude reaction mixtures, comparing the conversion of anhydride monomers to polymers. [c]Defined as mol anhydride consumed/(mol catalyst) x h. [d]Ester selectivity was determined by using the ^{1}H NMR spectra of *in-situ* polymers, comparing the polyether signal to a polyester signal. [e]Determined using ^{1}H NMR spectra of purified polymers with % epimer. = $\{2 \times A_{2.7\ ppm}/(A_{6.0\text{-}6.5\ ppm})\} \times 100$. [f]Calculated for 1 chloride initiator. [g]Identified by GPC, using a Wyatt MALS detector.

3.2 Air-free polymerizations

Table S10. Replicate catalytic reactions for the copolymerization of epoxides and cyclic anhydrides with dried ChCl as the catalyst.[a]

entry	anhydride	epoxide	conv.[b] (%)	TOF[c] (h^{-1})	ester sel.[d] (%)	epimer.[e] (%)	$M_{n,theo}$[f] (kDa)	$M_{n,exp}$[g] (kDa)	$Đ$[g]
1a	CPMA	BO	39	29	89	-	-	-	-
1b	CPMA	BO	52	39	92	-	-	-	-
1c	CPMA	BO	44	33	92	-	-	-	-
1d[h]	CPMA	BO	66	33	92	8	16.0	48.6	1.6
1e[h]	CPMA	BO	56	28	96	-	13.4	101.7	1.5
1f[h]	CPMA	BO	70	35	>99	-	16.5	143.9	1.3
2a	PA	BO	40	80	>99	-	-	-	-
2b	PA	BO	88	176	>99	-	-	-	-
2c	PA	BO	57	114	>99	-	-	-	-
2d	PA	BO	91	182	>99	-	-	-	-
2e	PA	BO	43	86	>99	-	-	-	-
2f[i]	PA	BO	>99	67	>99	-	-	-	-
3a	GA	BO	64	64	>99	-	-	-	-
3b	GA	BO	75	75	>99	-	-	-	-
3c	GA	BO	51	51	>99	-	-	-	-
3d	GA	BO	63	63	>99	-	-	-	-
4a	CPMA	CHO	8	6	80	-	-	-	-
4b	CPMA	CHO	15	11	92	-	-	-	-
4c	CPMA	CHO	28	21	84	-	-	-	-
4d	CPMA	CHO	27	20	>99	-	-	-	-
5a	PA	CHO	57	114	82	-	-	-	-
5b	PA	CHO	78	156	95	-	-	-	-
5c	PA	CHO	40	80	80	-	-	-	-
5d	PA	CHO	79	158	91	-	-	-	-
5e	PA	CHO	84	168	98	-	-	-	-
6a	GA	CHO	57	57	>99	-	-	-	-
6b	GA	CHO	71	51	>99	-	-	-	-
6c	GA	CHO	53	53	>99	-	-	-	-

[a][catalyst]:[anhydride]:[epoxide] was 1:100:500. Reactions were heated to 110 °C under neat conditions. The polymerizations with CPMA ran for 80 min, PA for 30 min, and GA for 60 min. [b]Determined using ^{1}H NMR spectra of crude reaction mixtures, comparing the conversion of anhydride monomers to polymers. [c]Defined as mol anhydride consumed/(mol catalyst) x h. [d]Ester selectivity was determined by using the ^{1}H NMR spectra of *in-situ* polymers, comparing the polyether signal to a polyester signal. [e]Determined using ^{1}H NMR spectra of purified polymers with % epimer. = {2 x $A_{2.7\ ppm}$/($A_{6.0\text{-}6.5\ ppm}$)} x 100. [f]Calculated for 1 chloride initiator. [g]Identified by GPC, using a Wyatt MALS detector. [h]Ran for 2 h in the same scale in order to obtain a pure isolated polymer for GPC analysis. [i]Ran for 1.5 h in the same scale in order to reach full conversion.

Table S11. Replicate catalytic reactions for the copolymerization of epoxides and cyclic anhydrides with dried ChBr as the catalyst.[a]

entry	anhydride	epoxide	conv.[b] (%)	TOF[c] (h^{-1})	ester sel.[d] (%)	epimer.[e] (%)	$M_{n,theo}$[f] (kDa)	$M_{n,exp}$[g] (kDa)	$Đ$[g]
1a	CPMA	BO	83	62	85	12	-	-	-
1b	CPMA		72	54	86	11	-	-	-
1c	CPMA		70	53	85	11	-	-	-
2a	PA		77	39	>99	-	-	-	-
2b	PA		85	43	>99	-	-	-	-
2c	PA		90	45	>99	-	-	-	-
3a	GA		61	61	>99	-	-	-	-
3b	GA		31	31	>99	-	-	-	-
3c	GA		37	37	>99	-	-	-	-
3d	GA		63	63	>99	-	-	-	-
4a	CPMA	CHO	39	29	>99	-	-	-	-
4b	CPMA		45	34	71	-	-	-	-
4c	CPMA		48	36	>99	-	-	-	-
5a	PA		78	156	96	-	-	-	-
5b	PA		76	152	98	-	-	-	-
5c	PA		81	162	94	-	-	-	-
6a	GA		53	53	>99	-	-	-	-
6b	GA		49	49	>99	-	-	-	-
6c	GA		59	59	>99	-	-	-	-

[a][catalyst]:[anhydride]:[epoxide] was 1:100:500. Reactions were heated to 110 °C under neat conditions. The polymerizations with CPMA ran for 80 min, PA for 30 min, and GA for 60 min. [b]Determined using ^{1}H NMR spectra of crude reaction mixtures, comparing the conversion of anhydride monomers to polymers. [c]Defined as mol anhydride consumed/(mol catalyst) x h. [d]Ester selectivity was determined by using the ^{1}H NMR spectra of *in-situ* polymers, comparing the polyether signal to a polyester signal. [e]Determined using ^{1}H NMR spectra of purified polymers with % epimer. = $\{2 \times A_{2.7\ ppm}/(A_{6.0\text{-}6.5\ ppm})\} \times 100$. [f]Calculated for 1 chloride initiator. [g]Identified by GPC, using a Wyatt MALS detector.

Table S12. Replicate catalytic reactions for the copolymerization of epoxides and cyclic anhydrides with dried ChI as the catalyst.[a]

entry	anhydride	epoxide	conv.[b] (%)	TOF[c] (h^{-1})	ester sel.[d] (%)	epimer.[e] (%)	$M_{n,theo}$[f] (kDa)	$M_{n,exp}$[g] (kDa)	$Đ$[g]
1a	CPMA	BO	97	73	>99	10	-	-	-
1b	CPMA		95	71	>99	16	-	-	-
1c	CPMA		95	71	>99	12	-	-	-
2a	PA		78	156	>99	-	-	-	-
2b	PA		69	138	>99	-	-	-	-
2c	PA		75	150	>99	-	-	-	-
3a	GA		43	43	>99	-	-	-	-
3b	GA		41	41	>99	-	-	-	-
3c	GA		49	49	>99	-	-	-	-
4a	CPMA	CHO	50	38	>99	-	-	-	-
4b	CPMA		51	38	96	-	-	-	-
4c	CPMA		48	36	>99	-	-	-	-
5a	PA		70	140	91	-	-	-	-
5b	PA		85	170	92	-	-	-	-
5c	PA		81	162	95	-	-	-	-
6a	GA		60	60	75	-	-	-	-
6b	GA		63	63	75	-	-	-	-
6c	GA		49	49	73	-	-	-	-

[a][catalyst]:[anhydride]:[epoxide] was 1:100:500. Reactions were heated to 110 °C under neat conditions. The polymerizations with CPMA ran for 80 min, PA for 30 min, and GA for 60 min. [b]Determined using ^1H NMR spectra of crude reaction mixtures, comparing the conversion of anhydride monomers to polymers. [c]Defined as mol anhydride consumed/(mol catalyst) x h. [d]Ester selectivity was determined by using the ^1H NMR spectra of *in-situ* polymers, comparing the polyether signal to a polyester signal. [e]Determined using ^1H NMR spectra of purified polymers with % epimer. = {2 x $A_{2.7\ ppm}$/($A_{6.0\text{-}6.5\ ppm}$)} x 100. [f]Calculated for 1 chloride initiator. [g]Identified by GPC, using a Wyatt MALS detector.

Table S13. Replicate catalytic reactions for the copolymerization of epoxides and cyclic anhydrides with dried ChCl/urea DES as the catalyst.[a]

entry	anhydride	epoxide	conv.[b] (%)	TOF[c] (h^{-1})	ester sel.[d] (%)	epimer.[e] (%)	$M_{n,theo}$[f] (kDa)	$M_{n,exp}$[g] (kDa)	$Đ$[g]
1a	CPMA	BO	71	53	97	11	16.9	14.2	1.6
1b	CPMA		64	48	>99	9	15.1	15.6	1.6
1c	CPMA		59	44	91	-	14.4	20.6	2.1
1d	CPMA		66	50	82	12	16.6	48	1.7
1e[h]	CPMA		86	43	88	9	21.2	15.5	1.4
2a	PA		77	154	>99	-	-	-	-
2b	PA		72	144	>99	-	-	-	-
2c	PA		84	168	>99	-	-	-	-
2d[i]	PA		>99	67	>99	-	-	-	-
3a	GA		55	55	>99	-	-	-	-
3b	GA		61	61	>99	-	-	-	-
3c	GA		50	50	>99	-	-	-	-
4a	CPMA	CHO	50	38	93	-	-	-	-
4b	CPMA		51	38	93	-	-	-	-
4c	CPMA		49	37	93	-	-	-	-
5a	PA		95	190	88	-	-	-	-
5b	PA		>99	200	86	-	-	-	-
5c	PA		89	178	88	-	-	-	-
6a	GA		72	72	>99	-	-	-	-
6b	GA		54	54	>99	-	-	-	-
6c	GA		52	52	>99	-	-	-	-
6d	GA		40	40	>99	-	-	-	-

[a][catalyst]:[anhydride]:[epoxide] was 1:100:500. Reactions were heated to 110 °C under neat conditions. The polymerizations with CPMA ran for 80 min, PA for 30 min, and GA for 60 min. [b]Determined using ^1H NMR spectra of crude reaction mixtures, comparing the conversion of anhydride monomers to polymers. [c]Defined as mol anhydride consumed/(mol catalyst) x h. [d]Ester selectivity was determined by using the ^1H NMR spectra of *in-situ* polymers, comparing the polyether signal to a polyester signal. [e]Determined using ^1H NMR spectra of purified polymers with % epimer. = {2 x $A_{2.7\ ppm}$/($A_{6.0\text{-}6.5\ ppm}$)} x 100. [f]Calculated for 1 chloride initiator. [g]Identified by GPC, using a Wyatt MALS detector. [h]Ran for 2 h in the same scale in order to obtain a pure isolated polymer for GPC analysis. [i]Ran for 1.5 h in the same scale in order to reach full conversion.

Table S14. Replicate catalytic reactions for the copolymerization of epoxides and cyclic anhydrides with dried ChCl/EG DES as the catalyst.[a]

entry	anhydride	epoxide	conv.[b] (%)	TOF[c] (h^{-1})	ester sel.[d] (%)	epimer.[e] (%)	$M_{n,theo}$[f] (kDa)	$M_{n,exp}$[g] (kDa)	$Đ$[g]
1a	CPMA	BO	78	59	>99	9	18.4	11.3	1.5
1b	CPMA		68	51	>99	8	16.1	11.6	1.4
1c	CPMA		81	61	>99	-	19.1	7.9	1.2
1d	CPMA		77	58	>99	-	18.2	8.4	1.3
2a	PA		98	196	>99	-	-	-	-
2b	PA		96	192	>99	-	-	-	-
2c	PA		98	196	>99	-	-	-	-
2d[h]	PA		>99	67	>99	-	-	-	-
3a	GA		41	41	>99	-	-	-	-
3b	GA		67	67	>99	-	-	-	-
3c	GA		44	44	>99	-	-	-	-
3d	GA		34	34	>99				
4a	CPMA	CHO	59	44	>99	<1	-	-	-
4b	CPMA		63	47	>99	-	-	-	-
4c	CPMA		74	56	>99	-	-	-	-
4d	CPMA		95	71	89	10	-	-	-
5a	PA		>99	200	90	-	-	-	-
5b	PA		97	194	91	-	-	-	-
5c	PA		98	196	92	-	-	-	-
6a	GA		57	57	>99	-	-	-	-
6b	GA		52	52	>99	-	-	-	-
6c	GA		48	48	>99	-	-	-	-

[a][catalyst]:[anhydride]:[epoxide] was 1:100:500. Reactions were heated to 110 °C under neat conditions. The polymerizations with CPMA ran for 80 min, PA for 30 min, and GA for 60 min. [b]Determined using ^{1}H NMR spectra of crude reaction mixtures, comparing the conversion of anhydride monomers to polymers. [c]Defined as mol anhydride consumed/(mol catalyst) x h. [d]Ester selectivity was determined by using the ^{1}H NMR spectra of *in-situ* polymers, comparing the polyether signal to a polyester signal. [e]Determined using ^{1}H NMR spectra of purified polymers with % epimer. = {2 x $A_{2.7\ ppm}$/($A_{6.0\text{-}6.5\ ppm}$)} x 100. [f]Calculated for 1 chloride initiator. [g]Identified by GPC, using a Wyatt MALS detector. [h]Ran for 1.5 h in the same scale in order to reach full conversion.

Table S15. Replicate catalytic reactions for the copolymerization of epoxides and cyclic anhydrides with dried ChBr/urea DES as the catalyst.[a]

entry	anhydride	epoxide	conv.[b] (%)	TOF[c] (h^{-1})	ester sel.[d] (%)	epimer.[e] (%)	$M_{n,theo}$[f] (kDa)	$M_{n,exp}$[g] (kDa)	$Đ$[g]
1a	CPMA	BO	71	53	>99	11	-	-	-
1b	CPMA		64	48	>99	9	-	-	-
1c	CPMA		71	53	>99	9	-	-	-
2a	PA		83	166	>99	-	-	-	-
2b	PA		76	152	>99	-	-	-	-
2c	PA		81	162	>99	-	-	-	-
3a	GA		50	50	>99	-	-	-	-
3b	GA		56	56	>99	-	-	-	-
3c	GA		45	45	>99	-	-	-	-
4a	CPMA	CHO	58	44	76	<1	-	-	-
4b	CPMA		40	90	82	-	-	-	-
4c	CPMA		41	31	78	-	-	-	-
5a	PA		70	140	86	-	-	-	-
5b	PA		91	182	88	-	-	-	-
5c	PA		86	172	91	-	-	-	-
6a	GA		45	45	>99	-	-	-	-
6b	GA		41	41	>99	-	-	-	-
6c	GA		46	46	>99	-	-	-	-

[a][catalyst]:[anhydride]:[epoxide] was 1:100:500. Reactions were heated to 110 °C under neat conditions. The polymerizations with CPMA ran for 80 min, PA for 30 min, and GA for 60 min. [b]Determined using ^{1}H NMR spectra of crude reaction mixtures, comparing the conversion of anhydride monomers to polymers. [c]Defined as mol anhydride consumed/(mol catalyst) x h. [d]Ester selectivity was determined by using the ^{1}H NMR spectra of *in-situ* polymers, comparing the polyether signal to a polyester signal. [e]Determined using ^{1}H NMR spectra of purified polymers with % epimer. = $\{2 \times A_{2.7\ ppm}/(A_{6.0\text{-}6.5\ ppm})\} \times 100$. [f]Calculated for 1 chloride initiator. [g]Identified by GPC, using a Wyatt MALS detector.

Table S16. Replicate catalytic reactions for the copolymerization of epoxides and cyclic anhydrides with dried ChBr/EG DES as the catalyst.[a]

entry	anhydride	epoxide	conv.[b] (%)	TOF[c] (h^{-1})	ester sel.[d] (%)	epimer.[e] (%)	$M_{n,theo}$[f] (kDa)	$M_{n,exp}$[g] (kDa)	$Đ$[g]
1a	CPMA	BO	61	46	>99	8	-	-	-
1b	CPMA		>99	75	>99	9	-	-	-
1c	CPMA		76	57	>99	8	-	-	-
1d	CPMA		>99	75	>99	10	-	-	-
2a	PA		84	168	>99	-	-	-	-
2b	PA		>99	200	>99	-	-	-	-
2c	PA		98	196	>99	-	-	-	-
3a	GA		75	75	>99	-	-	-	-
3b	GA		57	57	>99	-	-	-	-
3c	GA		52	52	>99	-	-	-	-
4a	CPMA	CHO	53	40	>99	-	-	-	-
4b	CPMA		52	39	88	-	-	-	-
4c	CPMA		52	39	88	-	-	-	-
5a	PA		>99	200	93	-	-	-	-
5b	PA		95	190	96	-	-	-	-
5c	PA		>99	200	93	-	-	-	-
6a	GA		41	41	>99	-	-	-	-
6b	GA		35	35	>99	-	-	-	-
6c	GA		34	34	>99	-	-	-	-

[a][catalyst]:[anhydride]:[epoxide] was 1:100:500. Reactions were heated to 110 °C under neat conditions. The polymerizations with CPMA ran for 80 min, PA for 30 min, and GA for 60 min. [b]Determined using ^{1}H NMR spectra of crude reaction mixtures, comparing the conversion of anhydride monomers to polymers. [c]Defined as mol anhydride consumed/(mol catalyst) x h. [d]Ester selectivity was determined by using the ^{1}H NMR spectra of *in-situ* polymers, comparing the polyether signal to a polyester signal. [e]Determined using ^{1}H NMR spectra of purified polymers with % epimer. = {2 x $A_{2.7\ ppm}$/($A_{6.0\text{-}6.5\ ppm}$)} x 100. [f]Calculated for 1 chloride initiator. [g]Identified by GPC, using a Wyatt MALS detector.

Table S17. Replicate catalytic reactions for the copolymerization of epoxides and cyclic anhydrides with dried ChI/urea DES as the catalyst.[a]

entry	anhydride	epoxide	conv.[b] (%)	TOF[c] (h^{-1})	ester sel.[d] (%)	epimer.[e] (%)	$M_{n,theo}$[f] (kDa)	$M_{n,exp}$[g] (kDa)	$Đ$[g]
1a	CPMA	BO	42	32	96	<1	-	-	-
1b	CPMA		71	53	91	18	-	-	-
1c	CPMA		64	48	91	-	-	-	-
2a	PA		61	46	>99	-	-	-	-
2b	PA		64	48	>99	-	-	-	-
2c	PA		74	56	>99	-	-	-	-
3a	GA		59	59	>99	-	-	-	-
3b	GA		52	52	>99	-	-	-	-
3c	GA		49	49	>99	-	-	-	-
4a	CPMA	CHO	43	32	84	-	-	-	-
4b	CPMA		59	44	94	-	-	-	-
4c	CPMA		49	37	89	-	-	-	-
5a	PA		98	196	87	-	-	-	-
5b	PA		82	164	88	-	-	-	-
5c	PA		83	166	89	-	-	-	-
6a	GA		38	38	>99	-	-	-	-
6b	GA		34	34	>99	-	-	-	-
6c	GA		33	33	>99	-	-	-	-

[a][catalyst]:[anhydride]:[epoxide] was 1:100:500. Reactions were heated to 110 °C under neat conditions. The polymerizations with CPMA ran for 80 min, PA for 30 min, and GA for 60 min. [b]Determined using ^{1}H NMR spectra of crude reaction mixtures, comparing the conversion of anhydride monomers to polymers. [c]Defined as mol anhydride consumed/(mol catalyst) x h. [d]Ester selectivity was determined by using the ^{1}H NMR spectra of *in-situ* polymers, comparing the polyether signal to a polyester signal. [e]Determined using ^{1}H NMR spectra of purified polymers with % epimer. = {2 x $A_{2.7\ ppm}$/($A_{6.0\text{-}6.5\ ppm}$)} x 100. [f]Calculated for 1 chloride initiator. [g]Identified by GPC, using a Wyatt MALS detector.

Table S18. Replicate catalytic reactions for the copolymerization of epoxides and cyclic anhydrides with dried ChI/EG DES as the catalyst.[a]

entry	anhydride	epoxide	conv.[b] (%)	TOF[c] (h^{-1})	ester sel.[d] (%)	epimer.[e] (%)	$M_{n,theo}$[f] (kDa)	$M_{n,exp}$[g] (kDa)	$Đ$[g]
1a	CPMA	BO	90	68	>99	8	-	-	-
1b	CPMA		84	63	>99	-	-	-	-
1c	CPMA		97	73	>99	-	-	-	-
2a	PA		83	166	>99	-	-	-	-
2b	PA		79	158	>99	-	-	-	-
2c	PA		73	146	>99	-	-	-	-
3a	GA		48	48	>99	-	-	-	-
3b	GA		55	55	>99	-	-	-	-
3c	GA		69	69	>99	-	-	-	-
4a	CPMA	CHO	50	38	95	-	-	-	-
4b	CPMA		63	47	98	-	-	-	-
4c	CPMA		60	45	>99	-	-	-	-
4d	CPMA		53	40	93	-	-	-	-
5a	PA		93	186	92	-	-	-	-
5b	PA		92	184	88	-	-	-	-
5c	PA		96	192	93	-	-	-	-
6a	GA		47	47	>99	-	-	-	-
6b	GA		43	43	>99	-	-	-	-
6c	GA		41	41	>99	-	-	-	-

[a][catalyst]:[anhydride]:[epoxide] was 1:100:500. Reactions were heated to 110 °C under neat conditions. The polymerizations with CPMA ran for 80 min, PA for 30 min, and GA for 60 min. [b]Determined using ^1H NMR spectra of crude reaction mixtures, comparing the conversion of anhydride monomers to polymers. [c]Defined as mol anhydride consumed/(mol catalyst) x h. [d]Ester selectivity was determined by using the ^1H NMR spectra of *in-situ* polymers, comparing the polyether signal to a polyester signal. [e]Determined using ^1H NMR spectra of purified polymers with % epimer. = $\{2 \times A_{2.7\ ppm}/(A_{6.0\text{-}6.5\ ppm})\} \times 100$. [f]Calculated for 1 chloride initiator. [g]Identified by GPC, using a Wyatt MALS detector.

4. ^{1}H NMR spectra

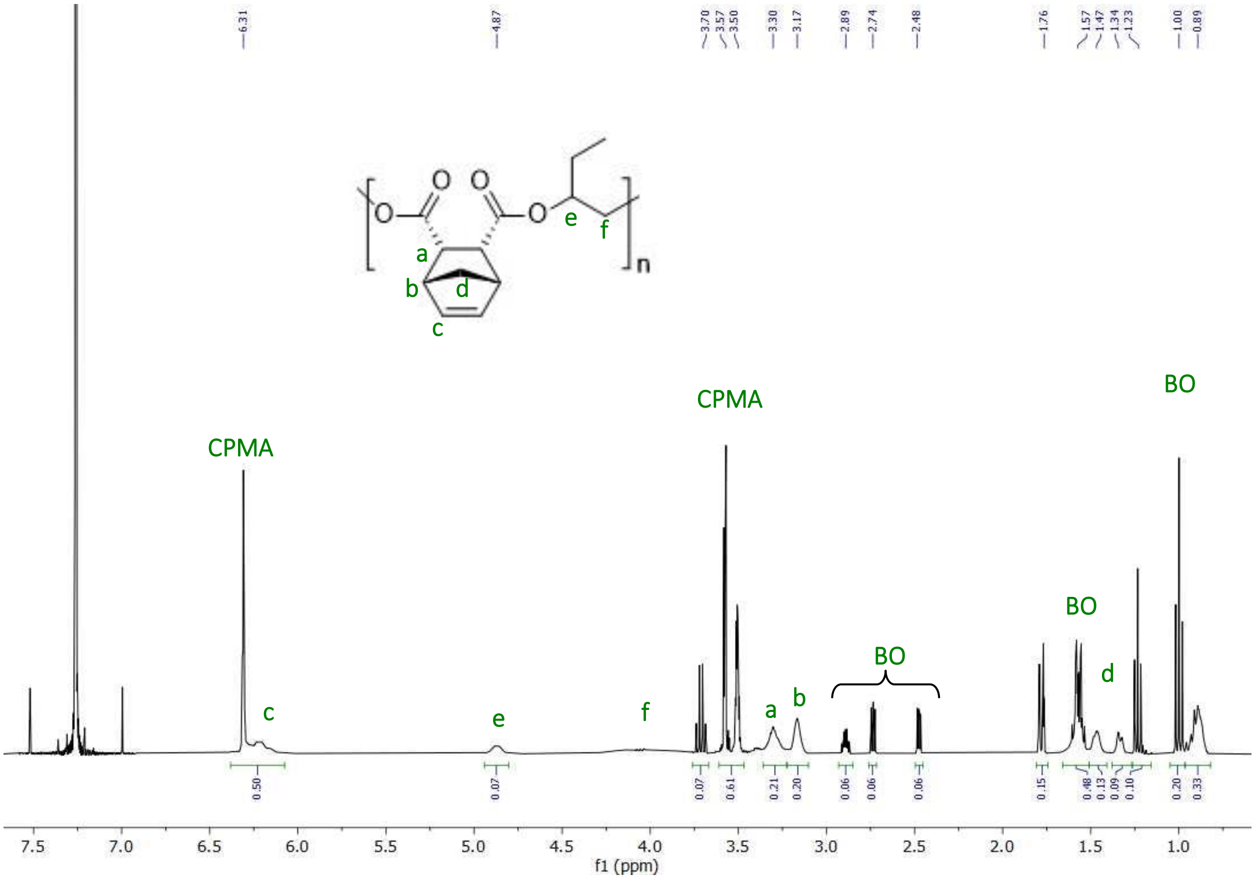

Figure S1. ^{1}H NMR spectrum of *in-situ* BO-*alt*-CPMA in $CDCl_3$ (**Table S1**, entry 1b).

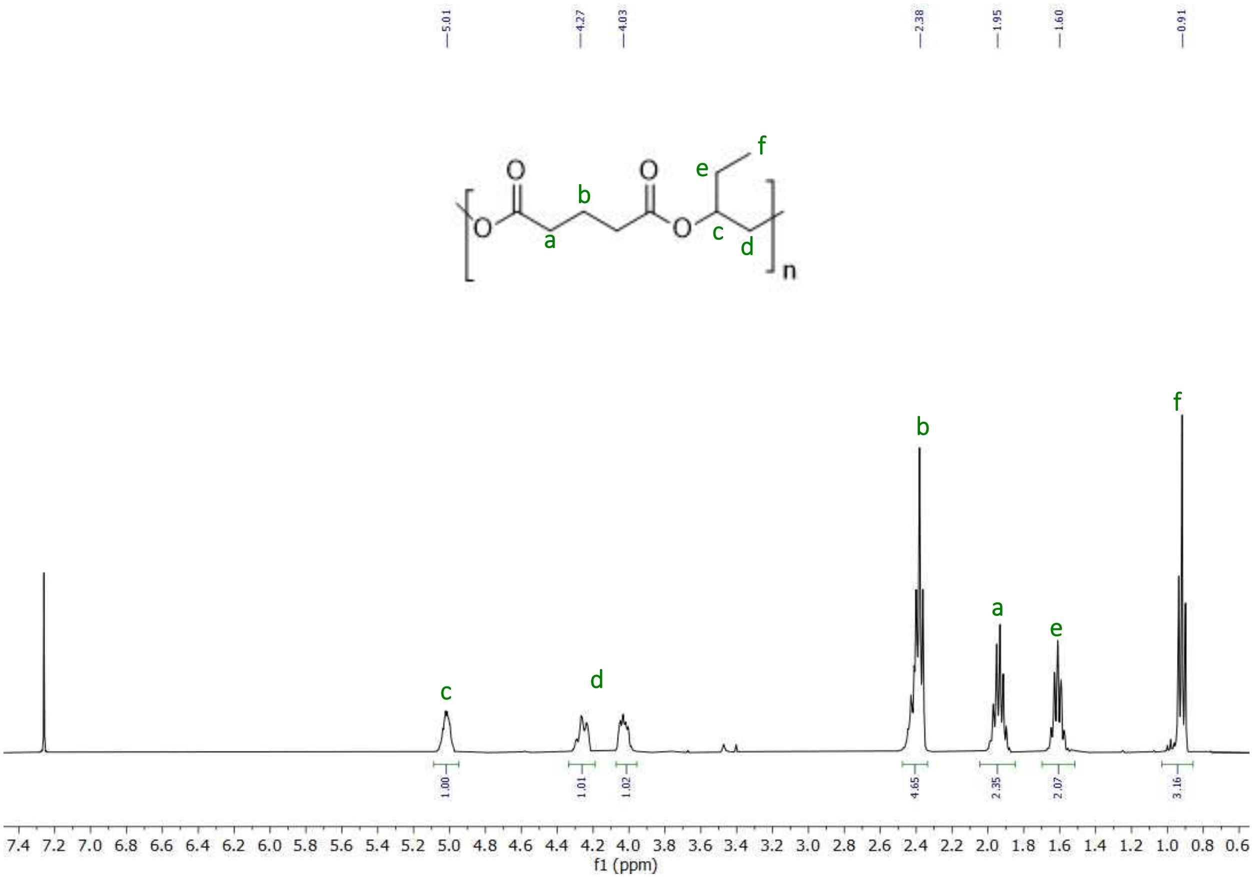

Figure S2. ^{1}H NMR spectrum of isolated BO-*alt*-GA in $CDCl_3$ (**Table S1**, entry 3a).

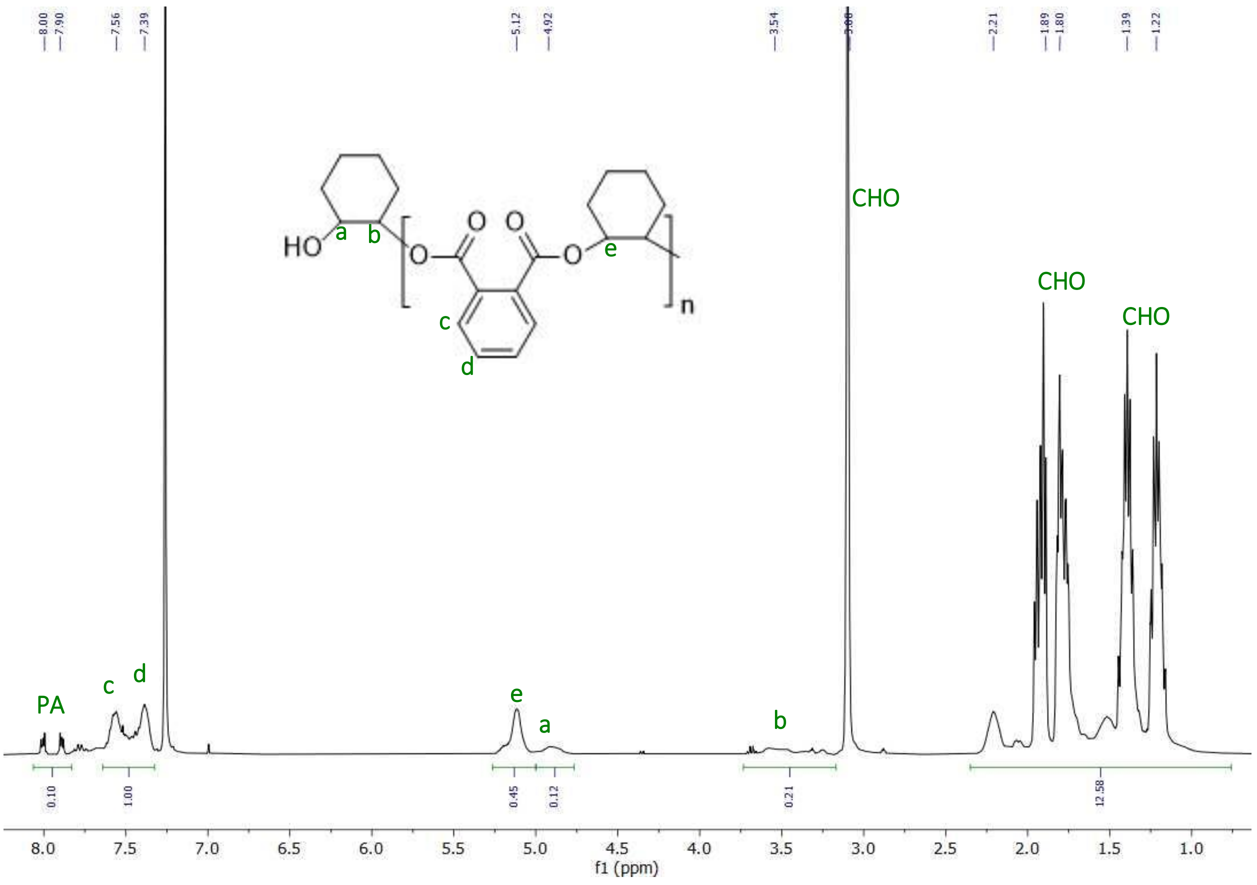

Figure S3. ^{1}H NMR spectrum of *in-situ* CHO-*alt*-PA in $CDCl_3$ (**Table S1**, entry 5a).

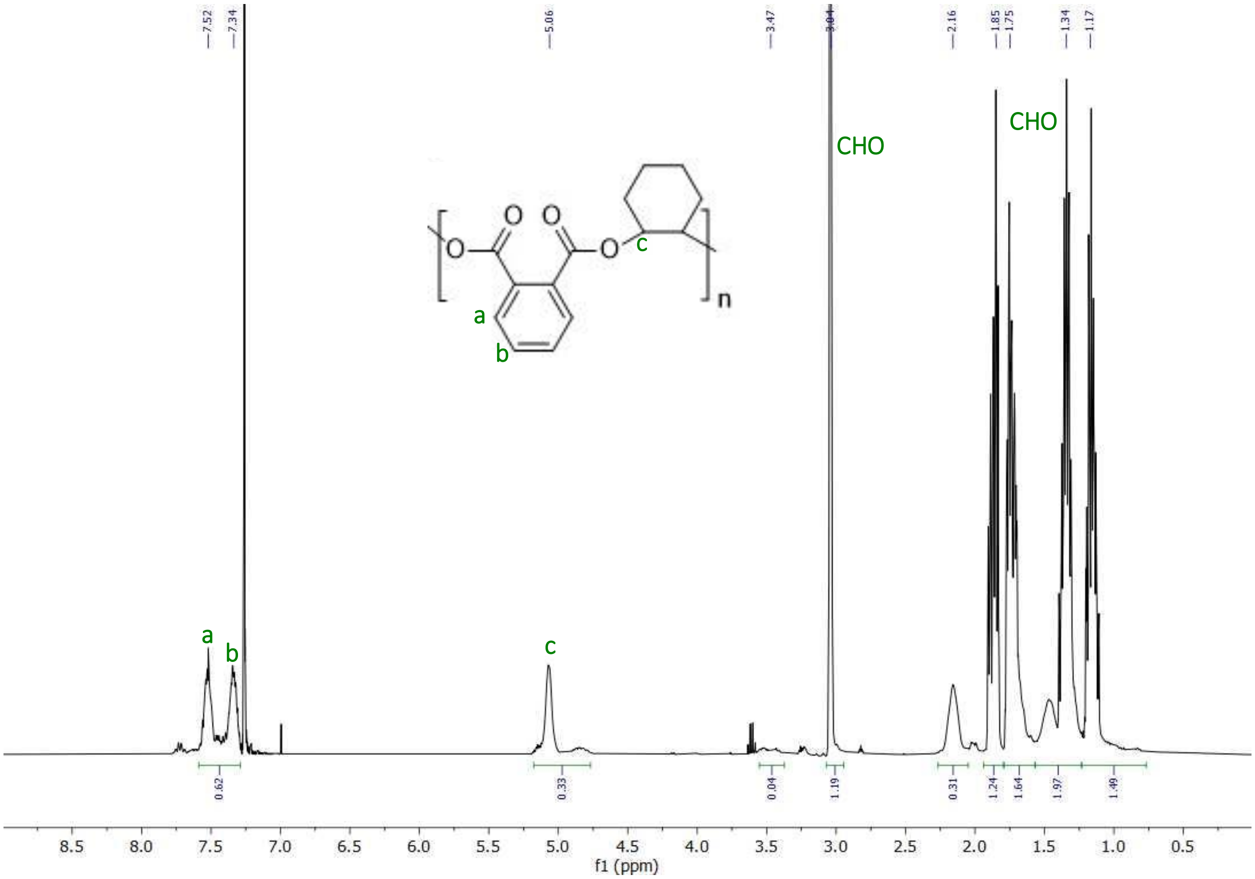

Figure S4. ^{1}H NMR spectrum of *in-situ* CHO-*alt*-PA in $CDCl_3$ (**Table S1**, entry 5b).

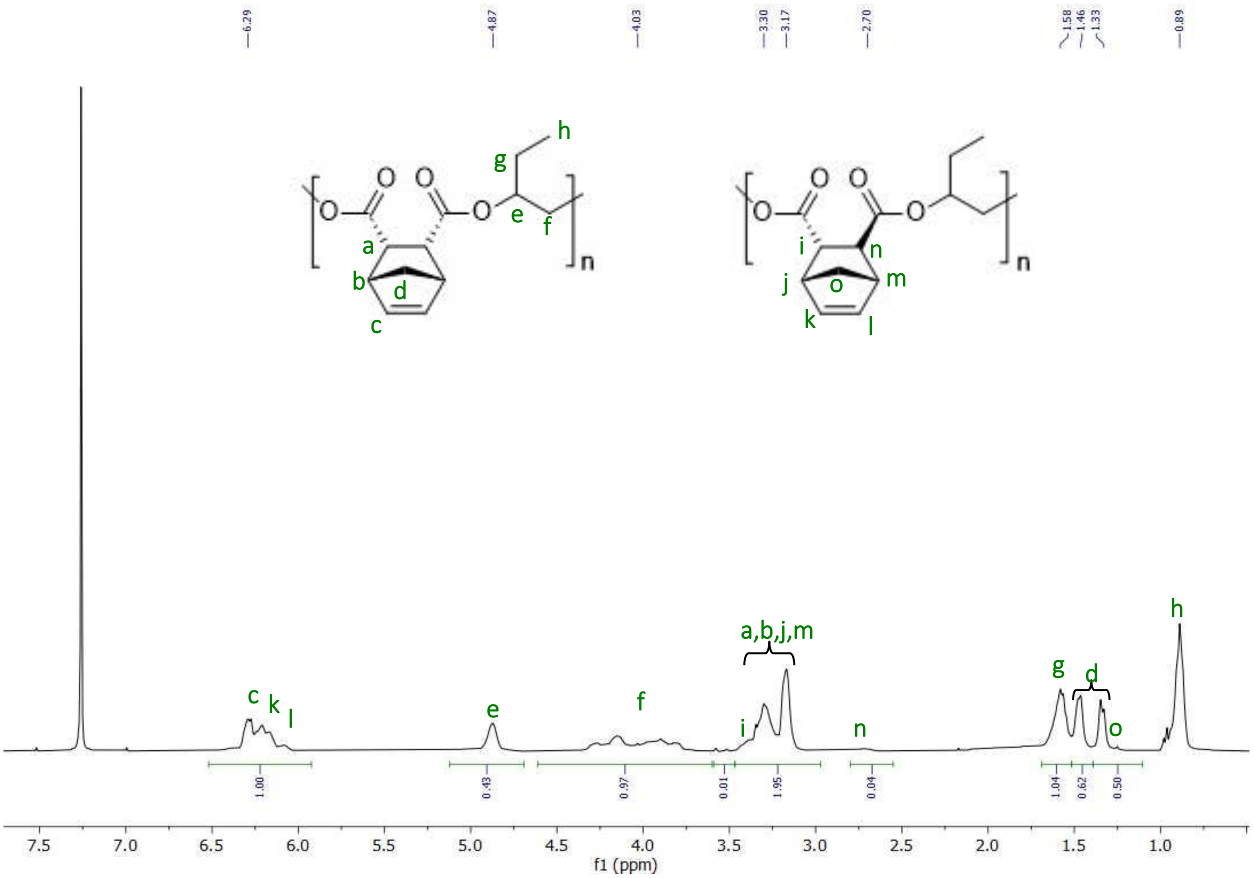

Figure S5. ^{1}H NMR spectrum of isolated BO-*alt*-CPMA in $CDCl_3$ (**Table S2**, entry 1a). Epimerization can be calculated by % epimer. = $\{2 \times A_{2.7\ ppm}/(A_{6.0\text{-}6.5\ ppm})\} \times 100$.

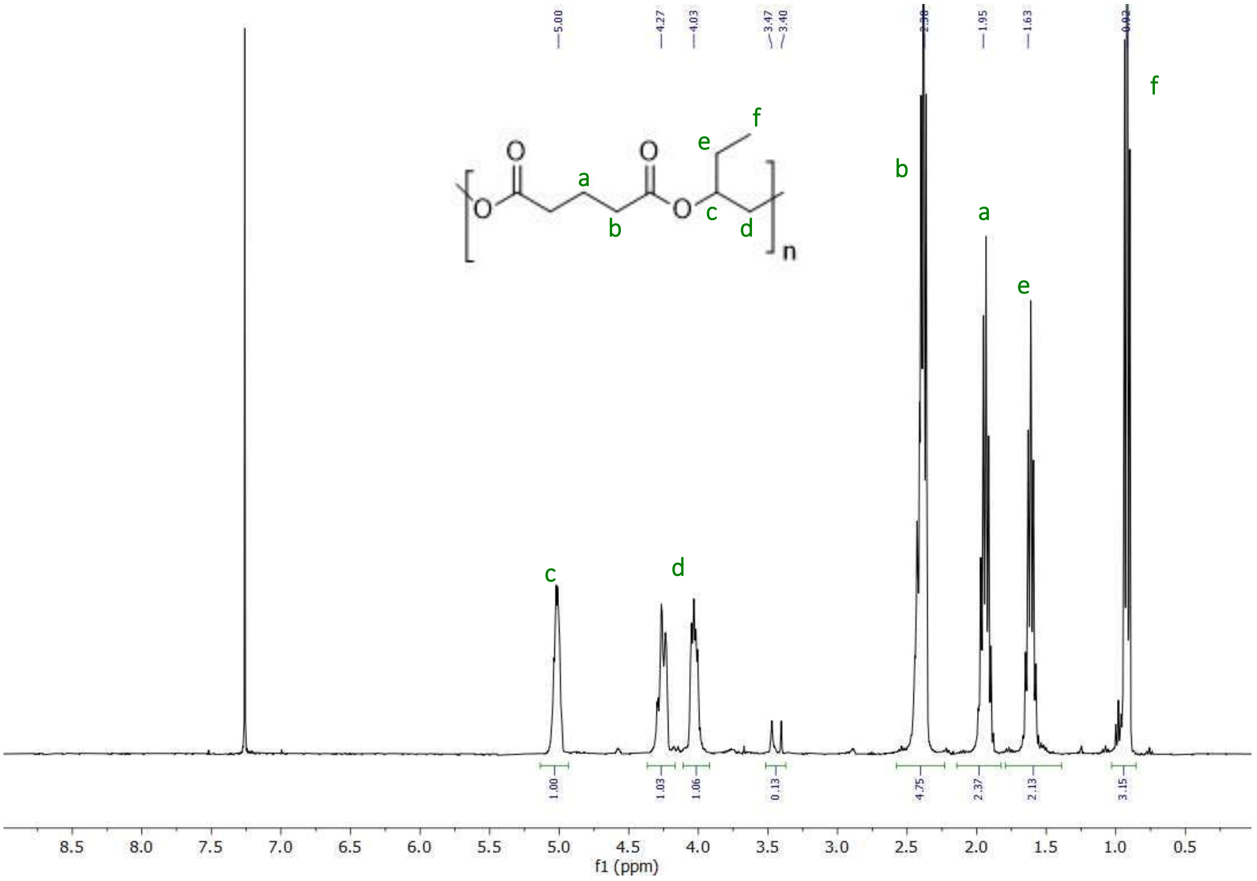

Figure S6. ^{1}H NMR spectrum of isolated BO-*alt*-GA in $CDCl_3$ (**Table S2**, entry 3a).

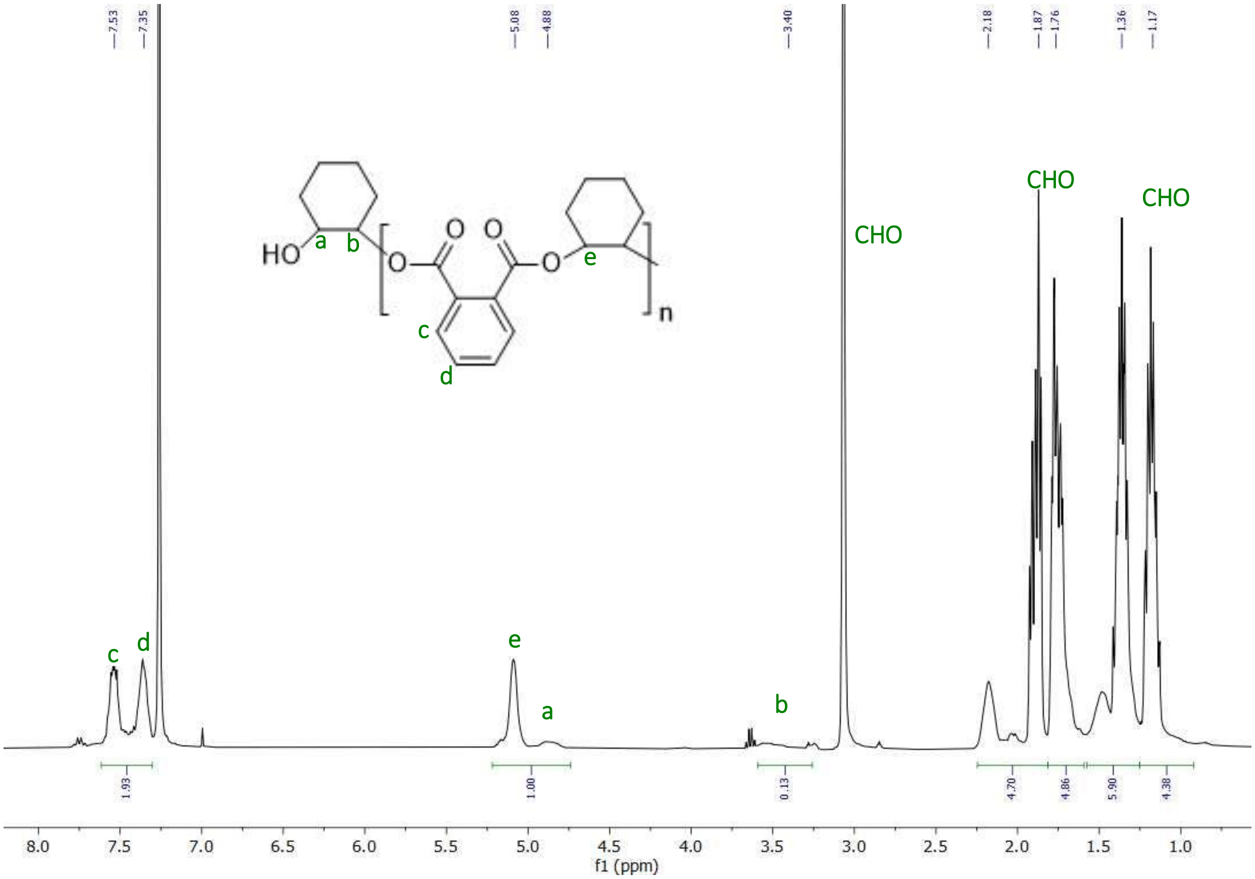

Figure S7. ^{1}H NMR spectrum of *in-situ* CHO-*alt*-PA in $CDCl_3$ (**Table S2**, entry 5b).

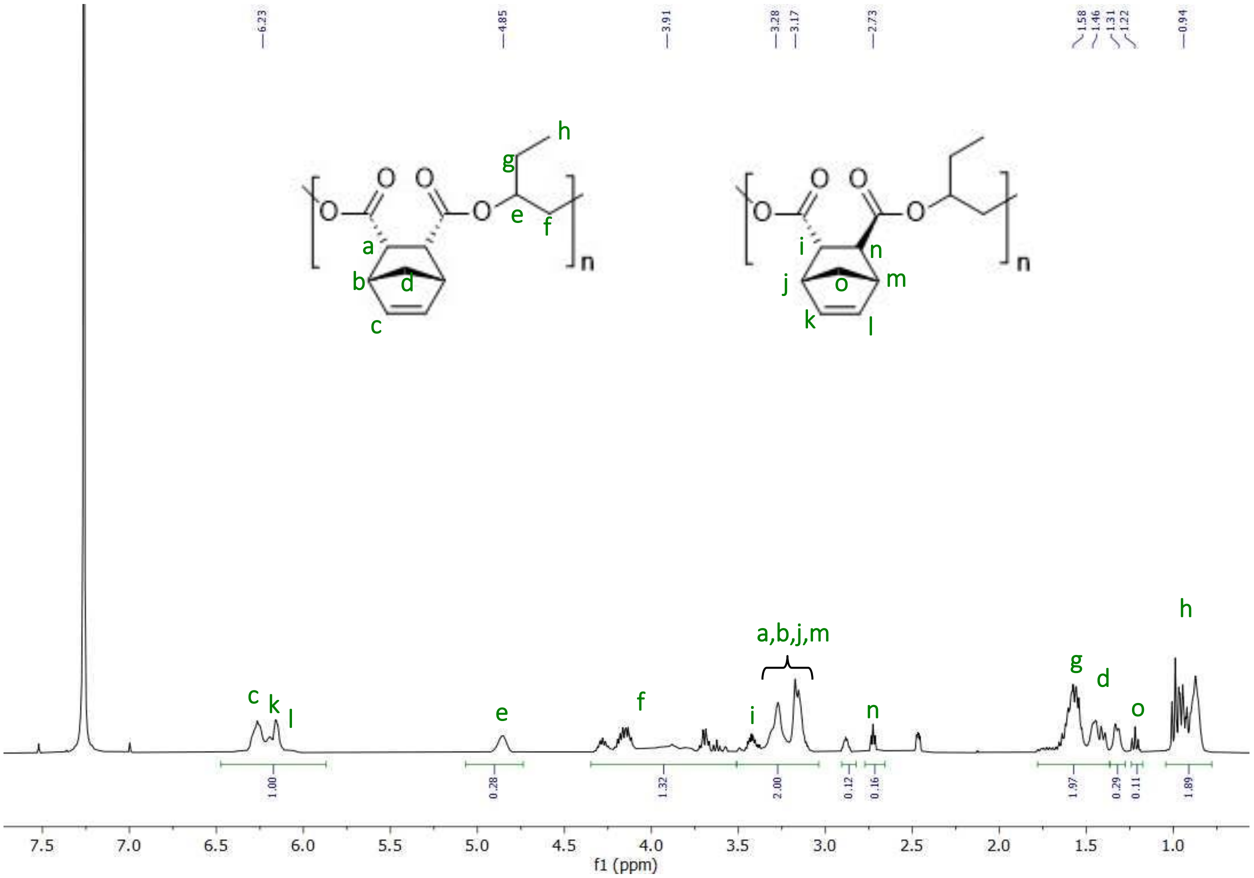

Figure S8. ^{1}H NMR spectrum of isolated BO-*alt*-CPMA in $CDCl_3$ (**Table S3**, entry 1a).

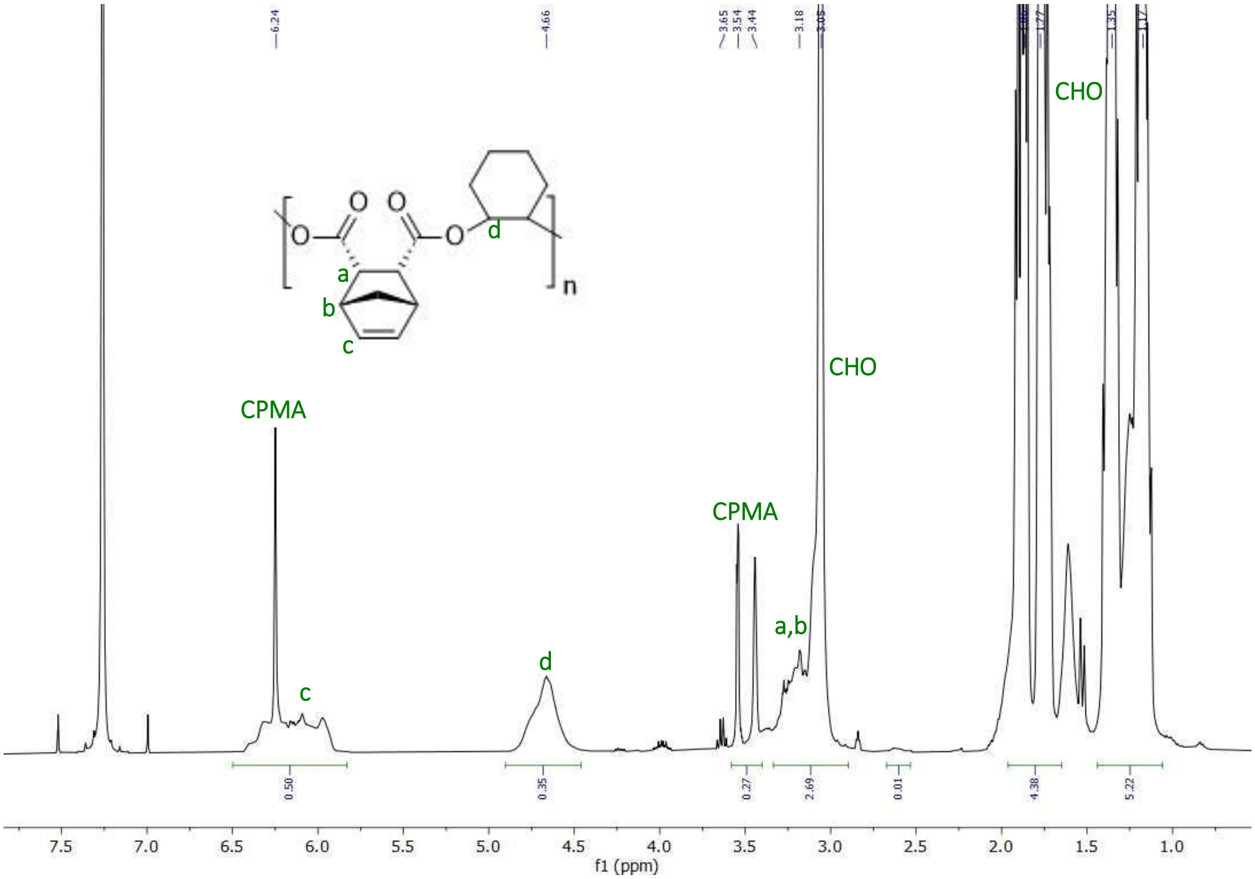

Figure S9. ^{1}H NMR spectrum of *in-situ* CHO-*alt*-CPMA in $CDCl_3$ (**Table S3**, entry 4a).

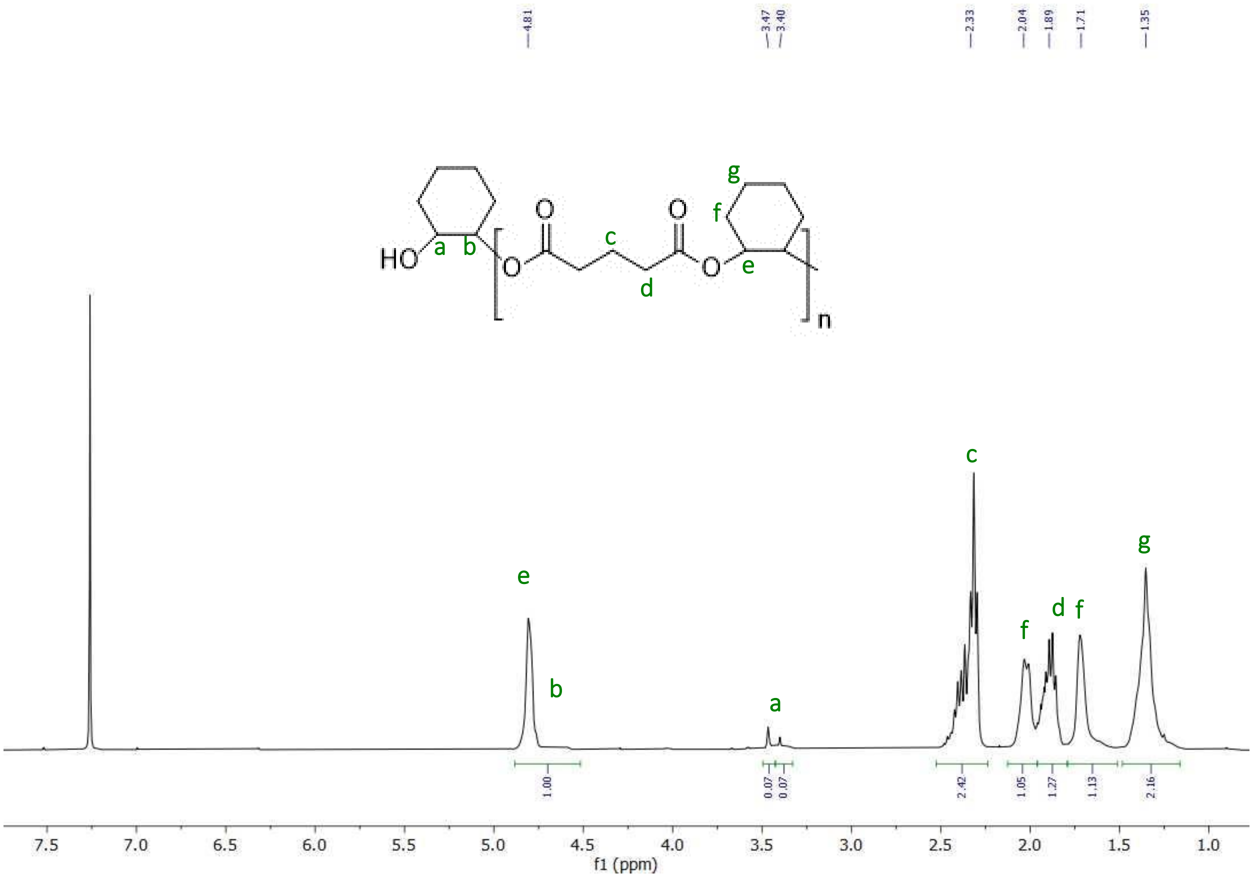

Figure S10. ^{1}H NMR spectrum of isolated CHO-*alt*-GA in $CDCl_3$ (**Table S3**, entry 6a).

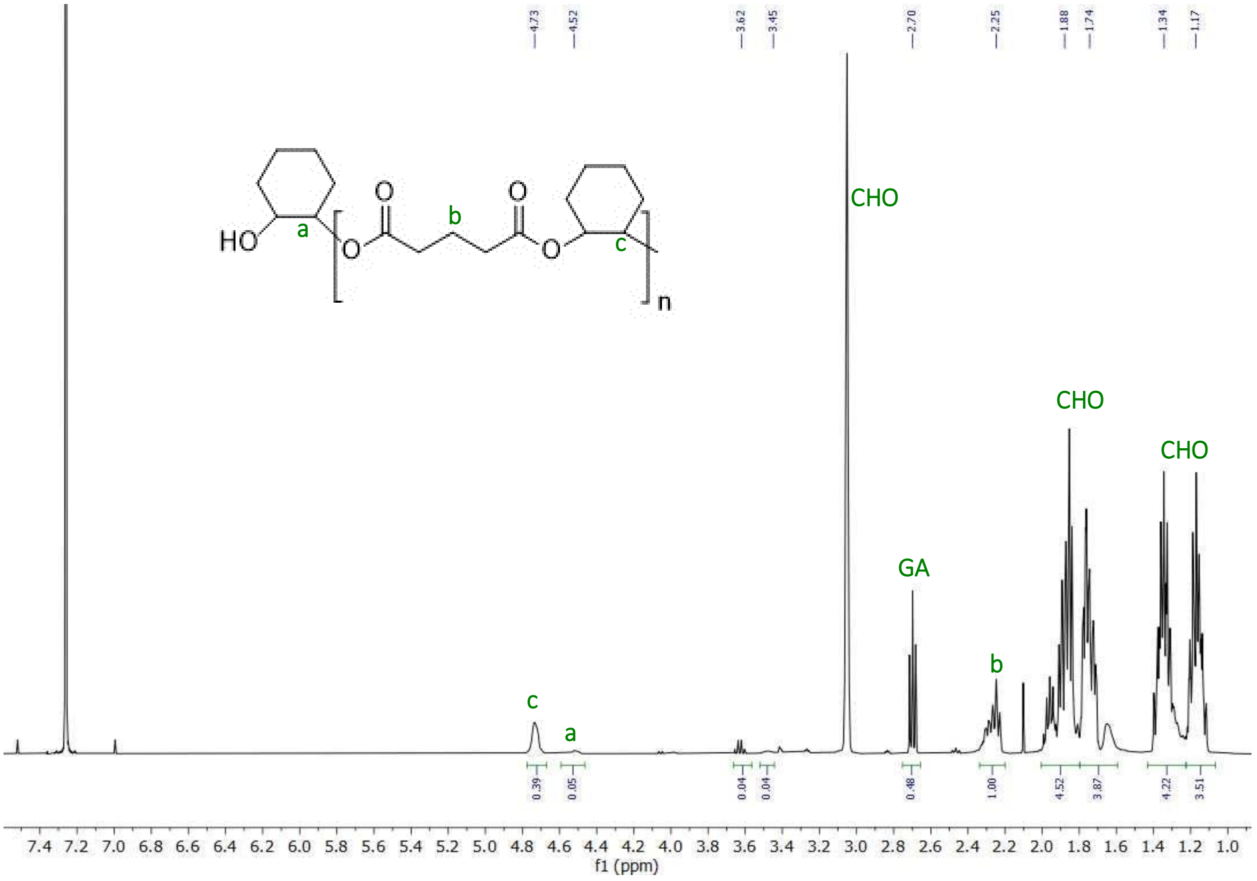

Figure S11. ^{1}H NMR spectrum of *in-situ* CHO-*alt*-GA in $CDCl_3$ (**Table S3**, entry 6b).

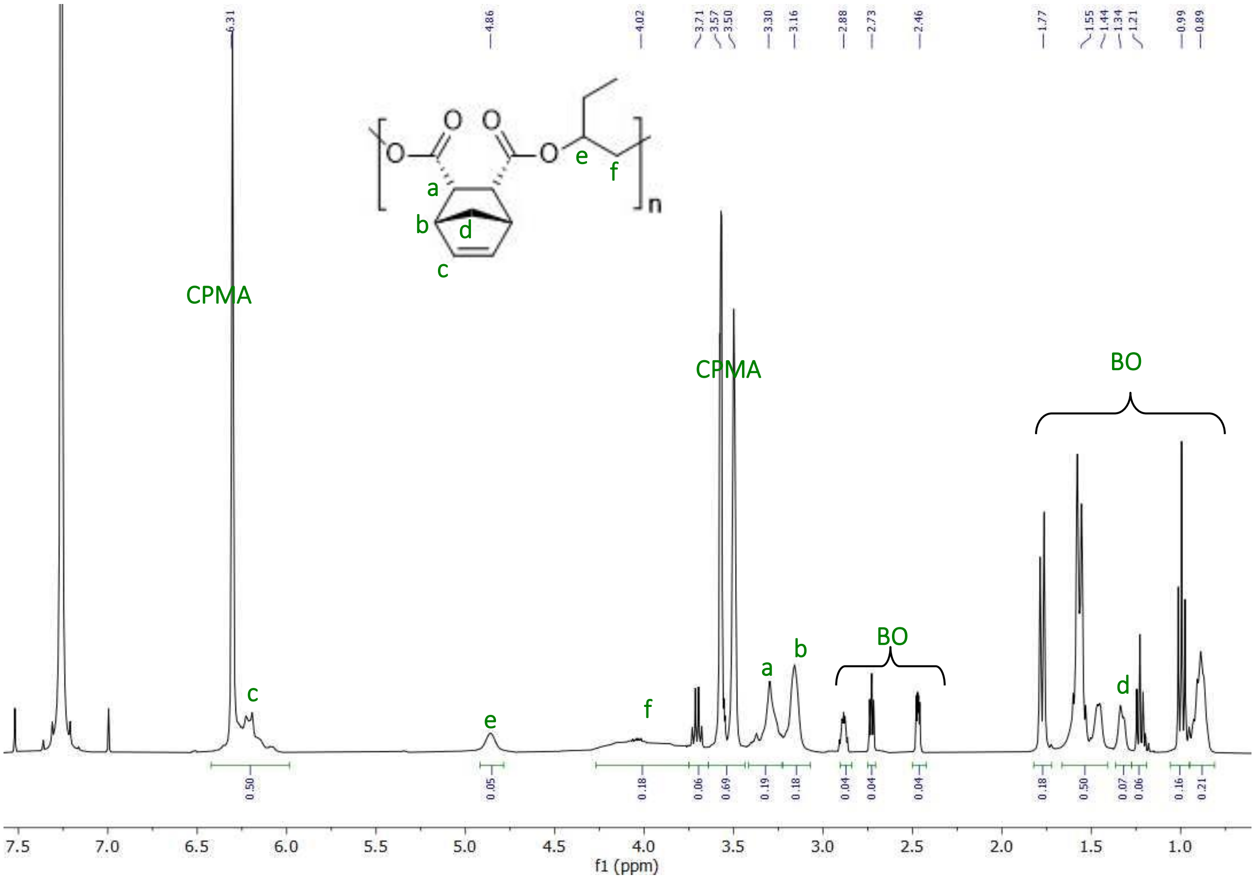

Figure S12. ^{1}H NMR spectrum of *in-situ* BO-*alt*-CPMA in $CDCl_3$ (**Table S4**, entry 1a).

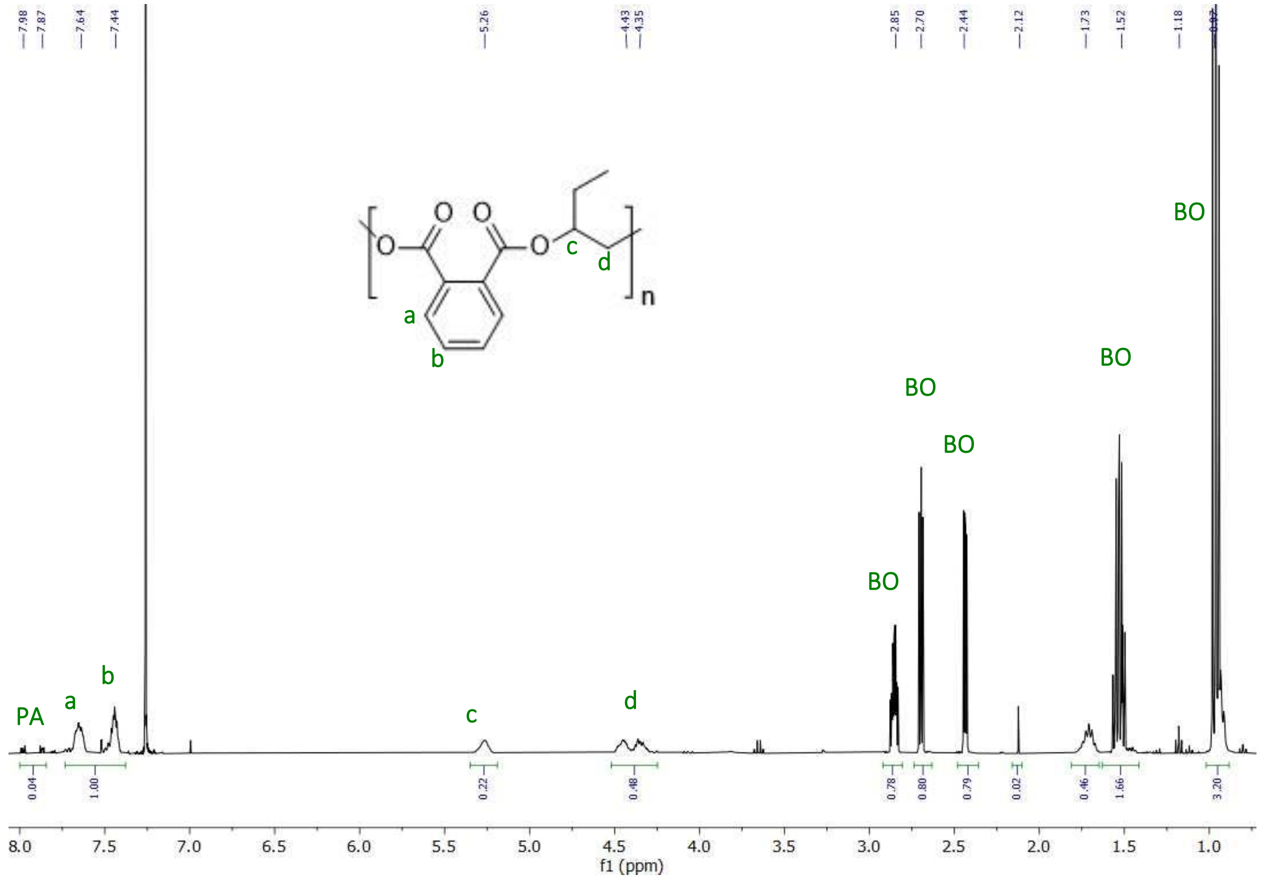

Figure S13. ^{1}H NMR spectrum of *in-situ* BO-*alt*-PA in $CDCl_3$ (**Table S4**, entry 2c).

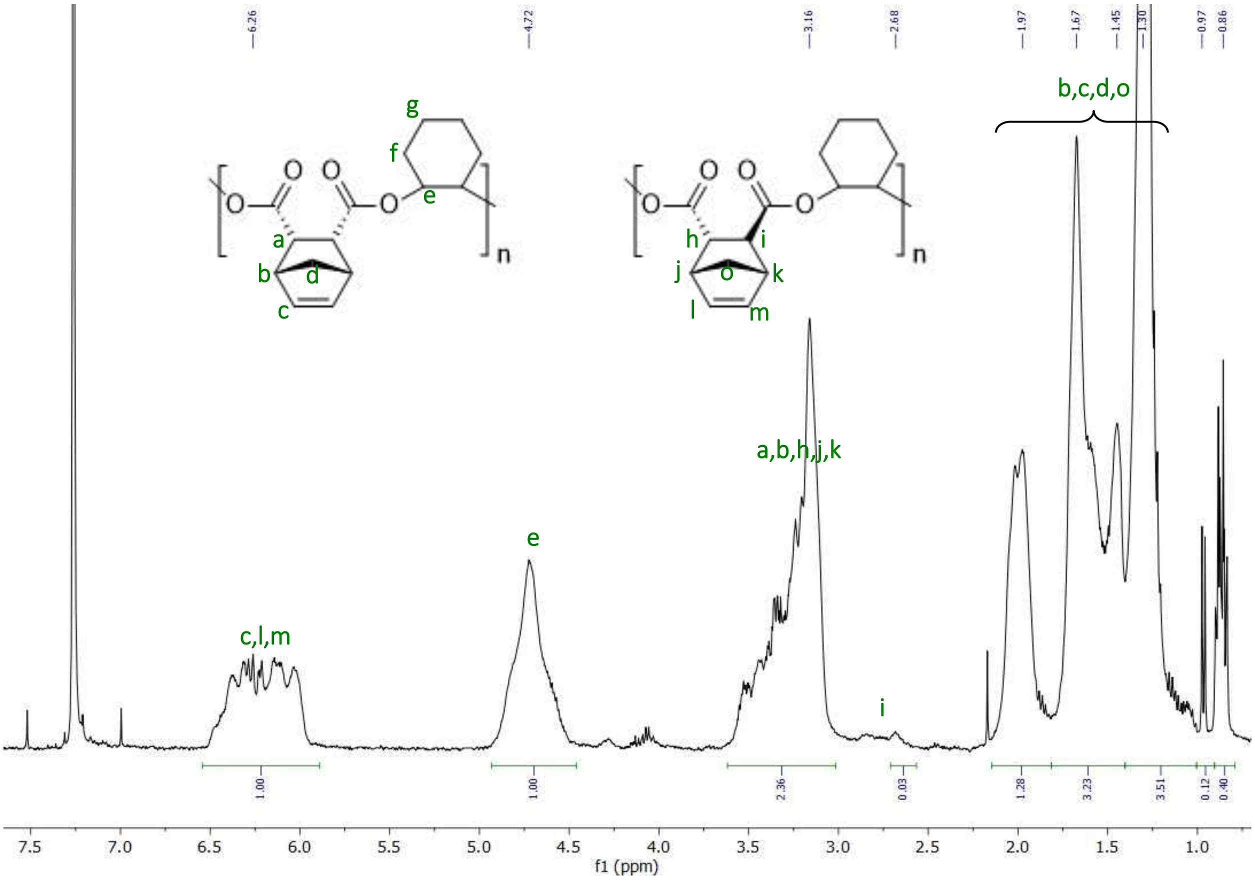

Figure S14. ^{1}H NMR spectrum of isolated CHO-*alt*-CPMA in $CDCl_3$ (**Table S4**, entry 4b).

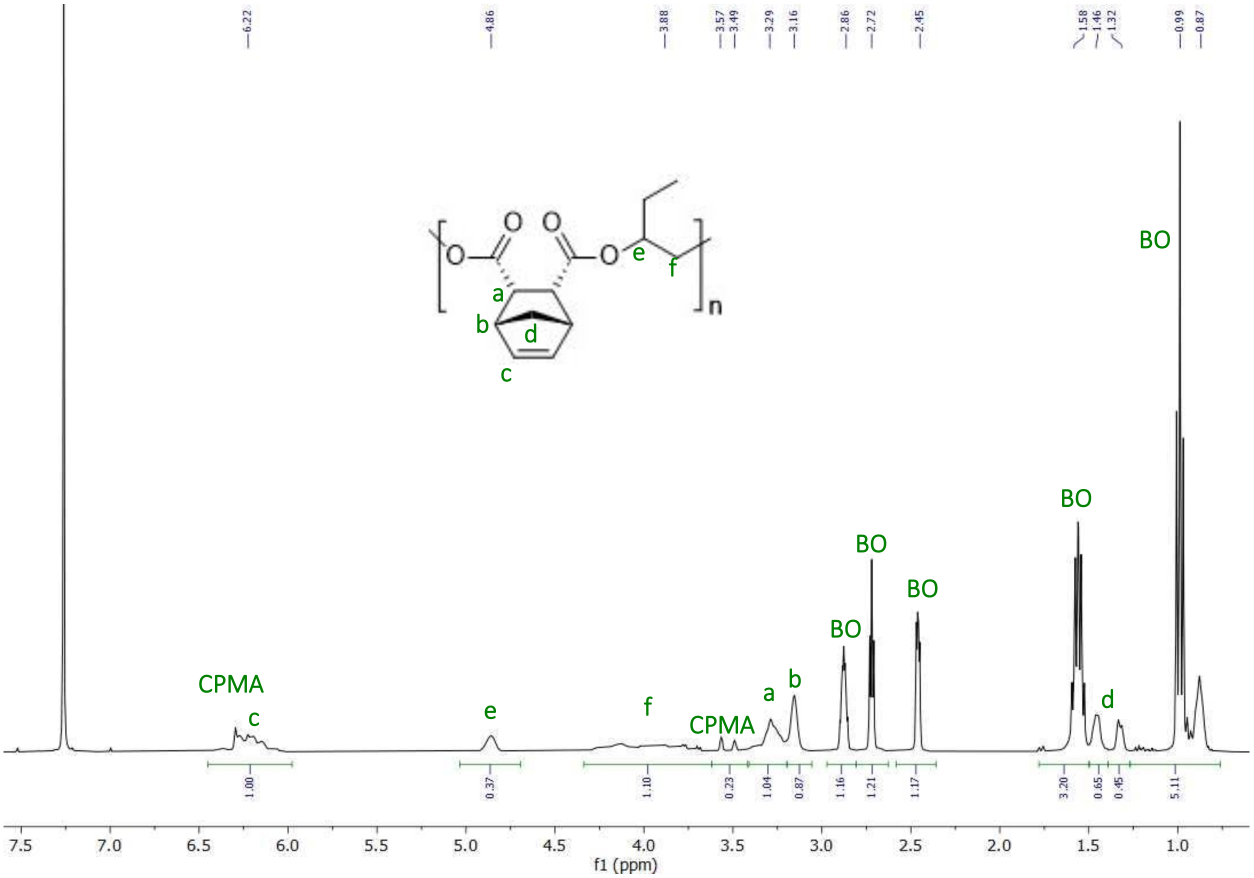

Figure S15. ^{1}H NMR spectrum of *in-situ* BO-*alt*-CPMA in $CDCl_3$ (**Table S5**, entry 1a).

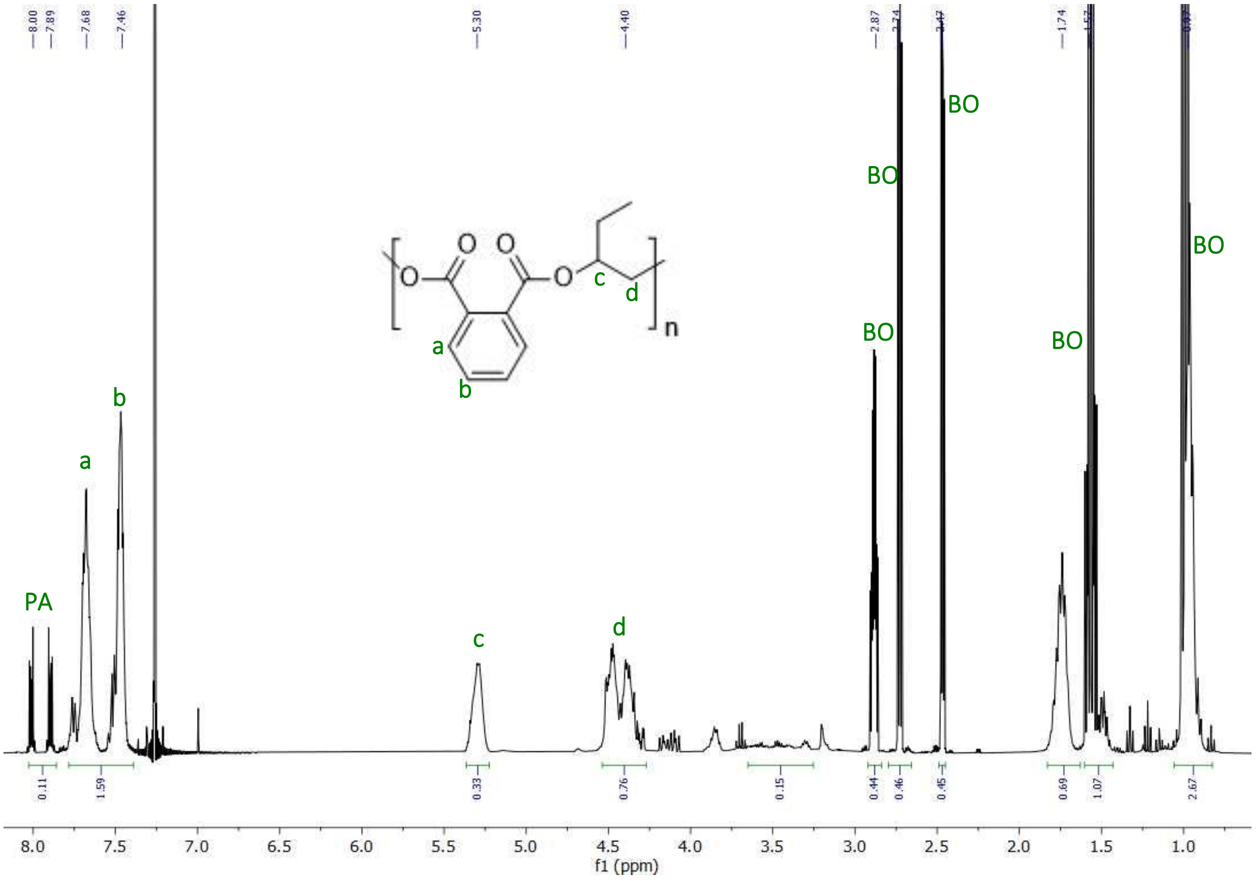

Figure S16 ^{1}H NMR spectrum of *in-situ* BO-*alt*-PA in $CDCl_3$ (**Table S5**, entry 2a).

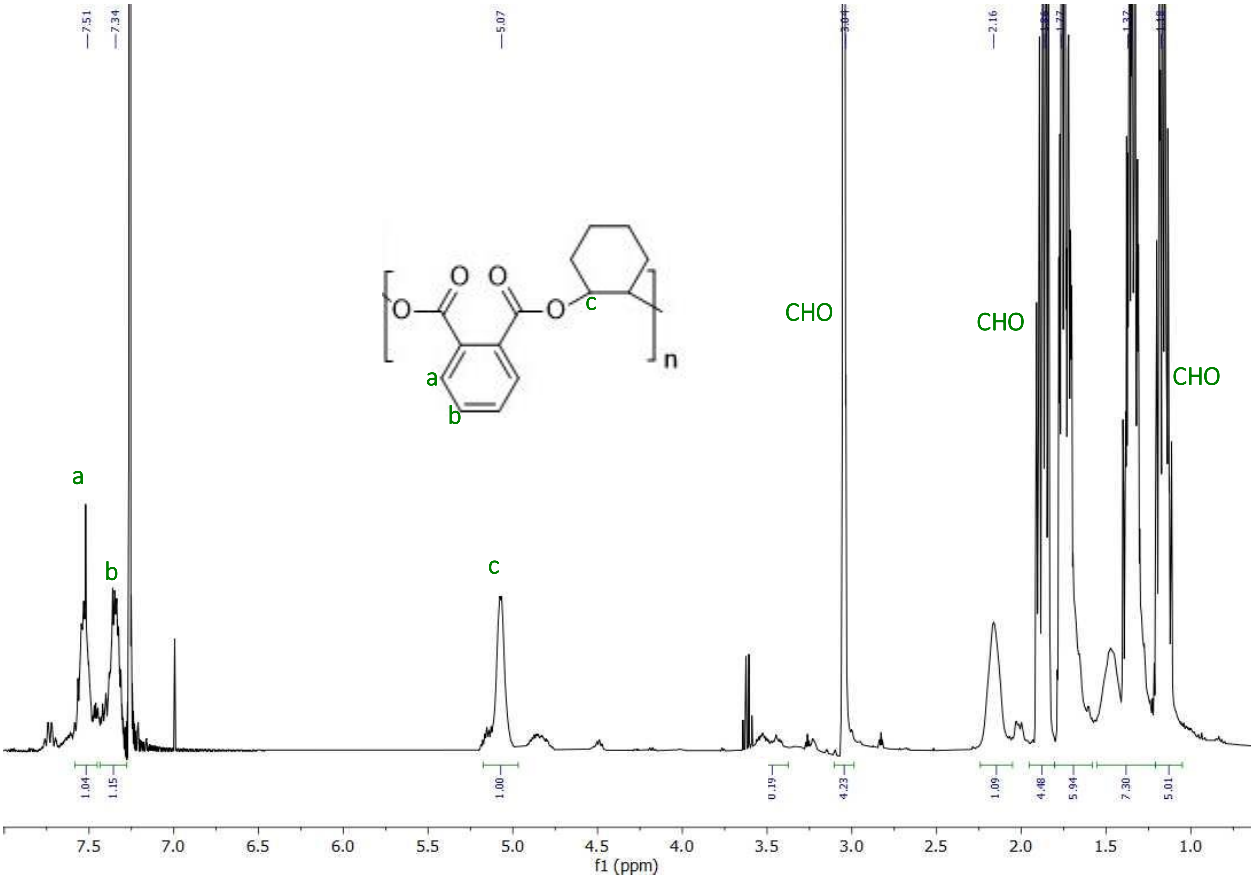

Figure S17. ^{1}H NMR spectrum of *in-situ* CHO-*alt*-PA in $CDCl_3$ (**Table S5**, entry 5a).

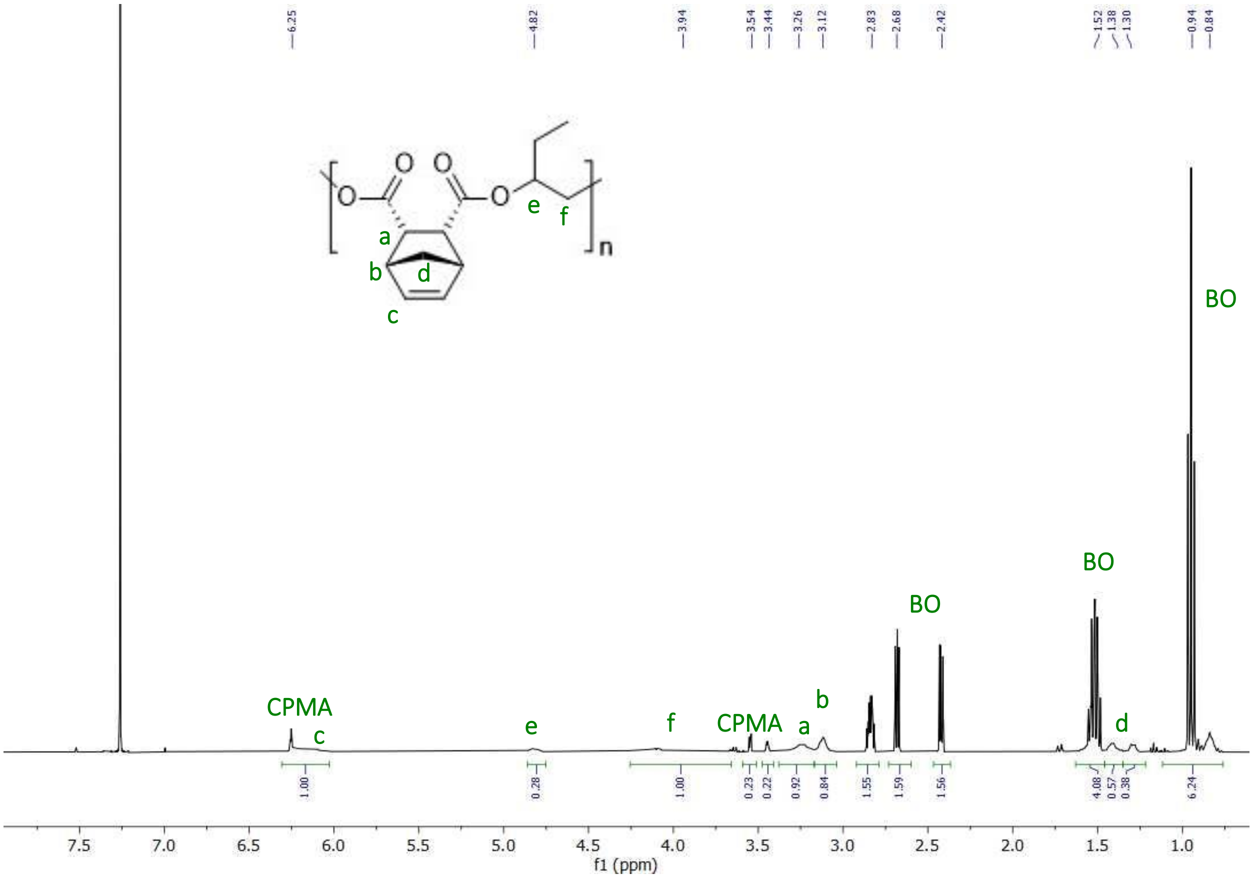

Figure S18. ^{1}H NMR spectrum of *in-situ* BO-*alt*-CPMA in $CDCl_3$ (**Table S6**, entry 1b).

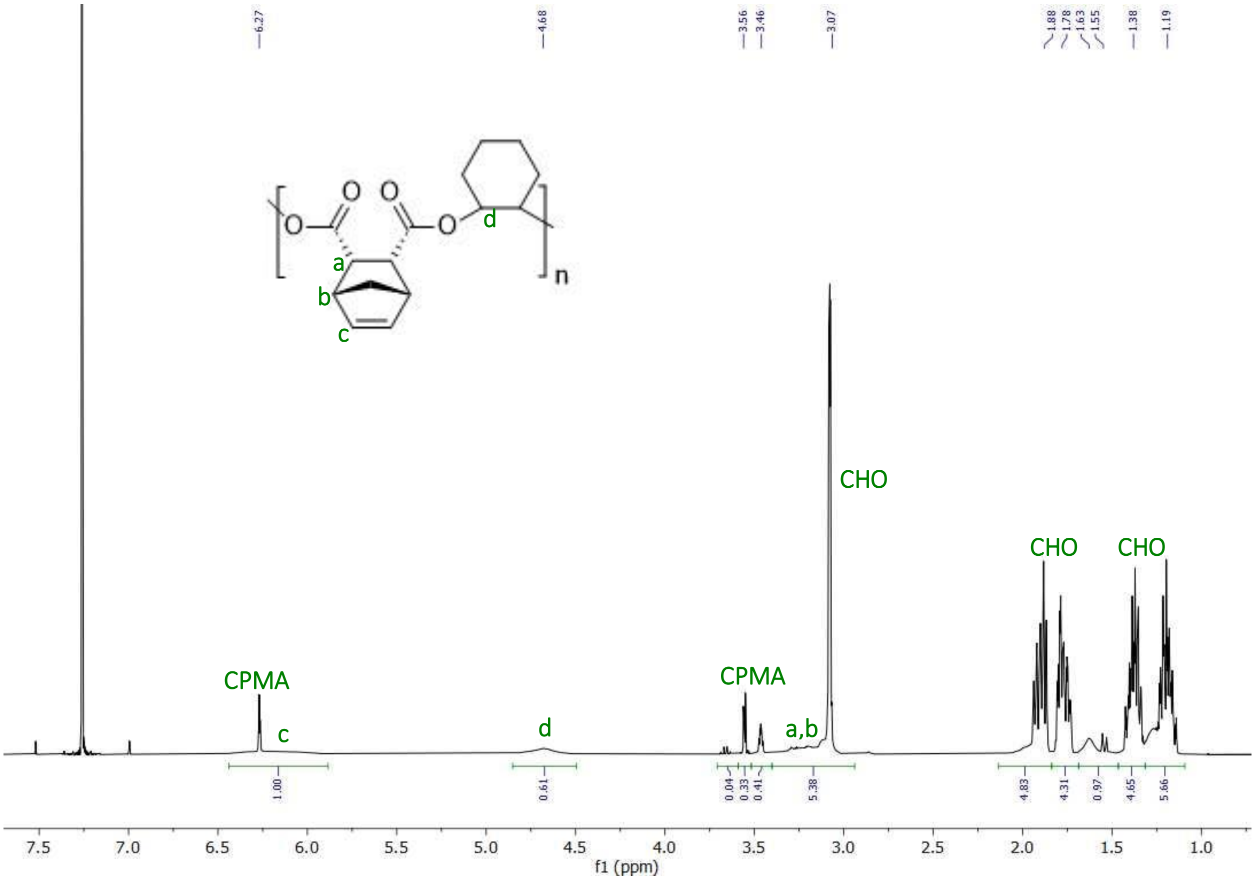

Figure S19. ^{1}H NMR spectrum of *in-situ* CHO-*alt*-CPMA in $CDCl_3$ (**Table S6**, entry 4a).

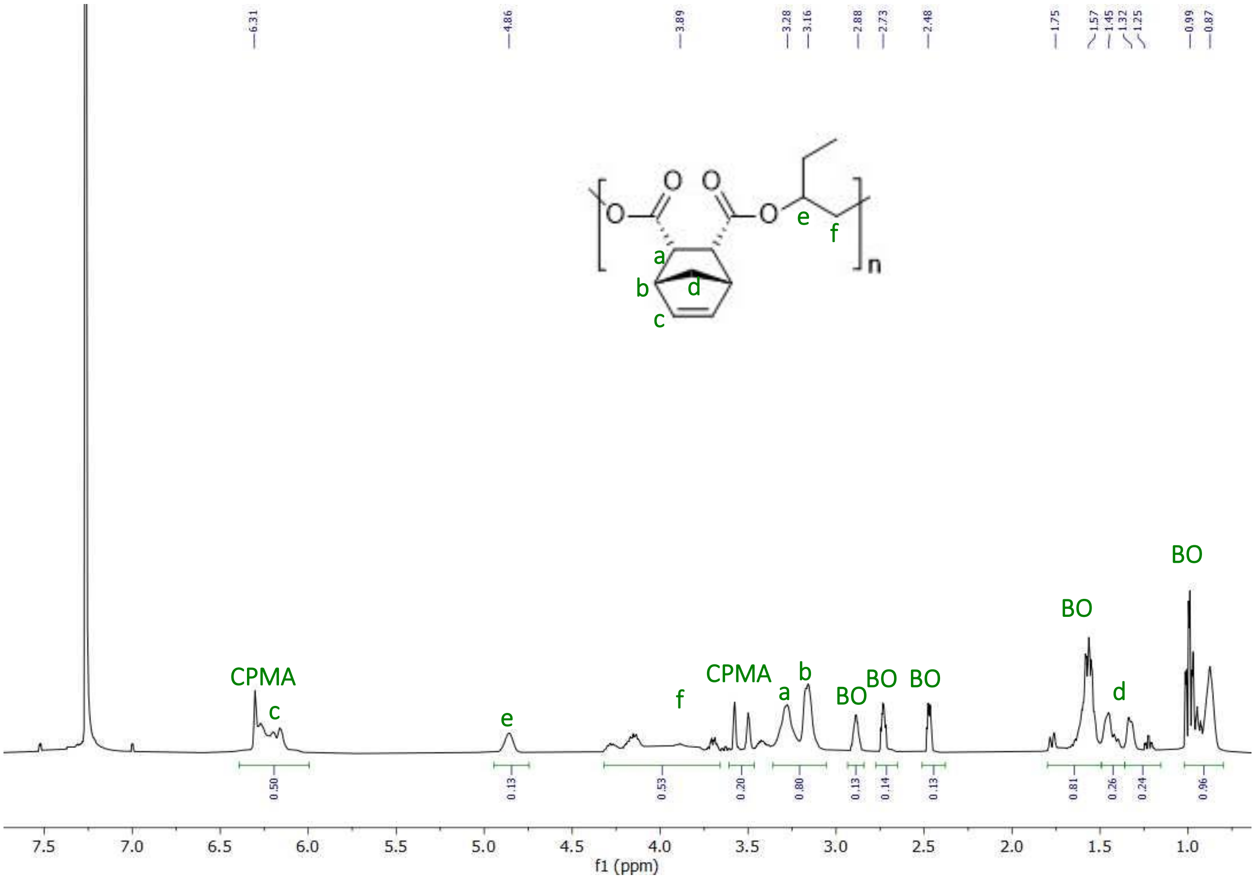

Figure S20. ^{1}H NMR spectrum of *in-situ* BO-*alt*-CPMA in $CDCl_3$ (**Table S7**, entry 1a).

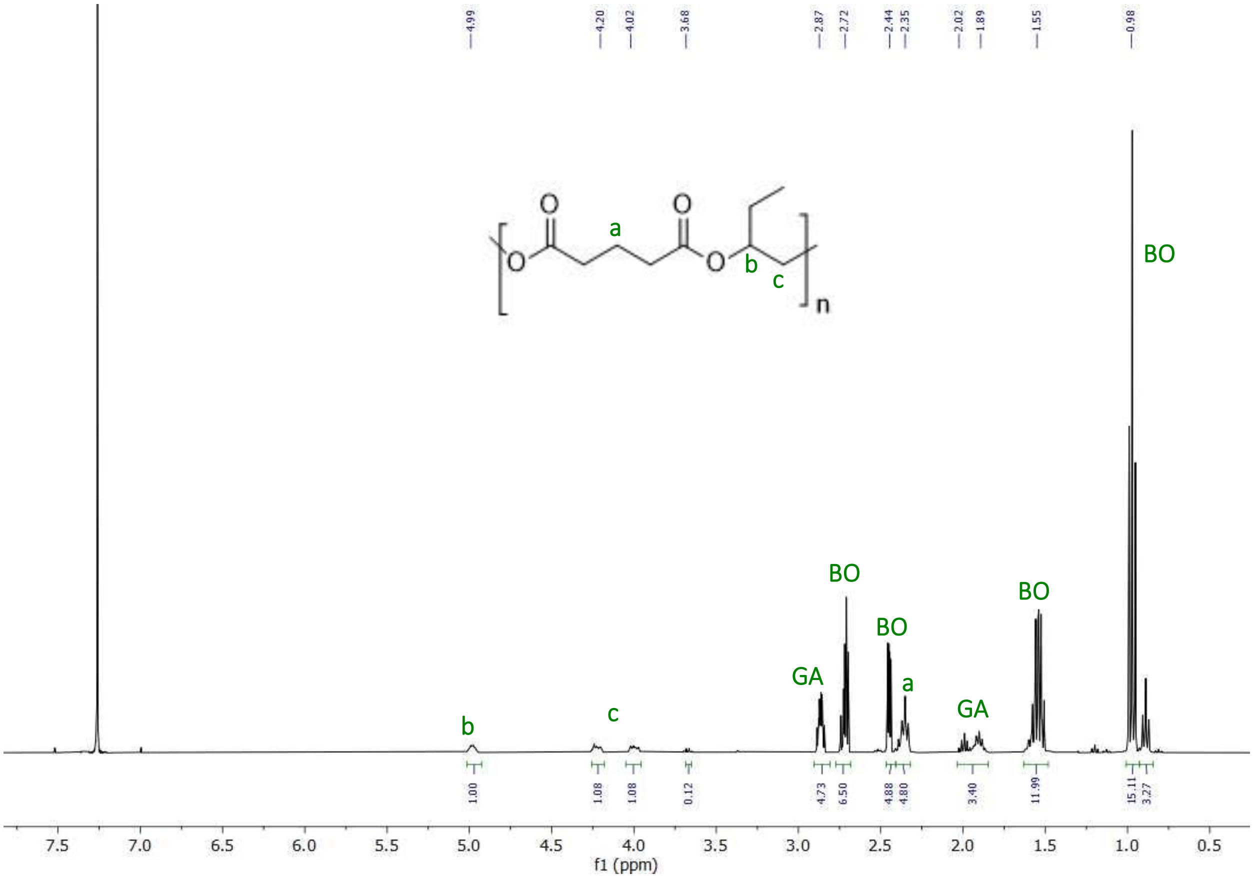

Figure S21. ^{1}H NMR spectrum of *in-situ* BO-*alt*-GA in $CDCl_3$ (**Table S7**, entry 3a).

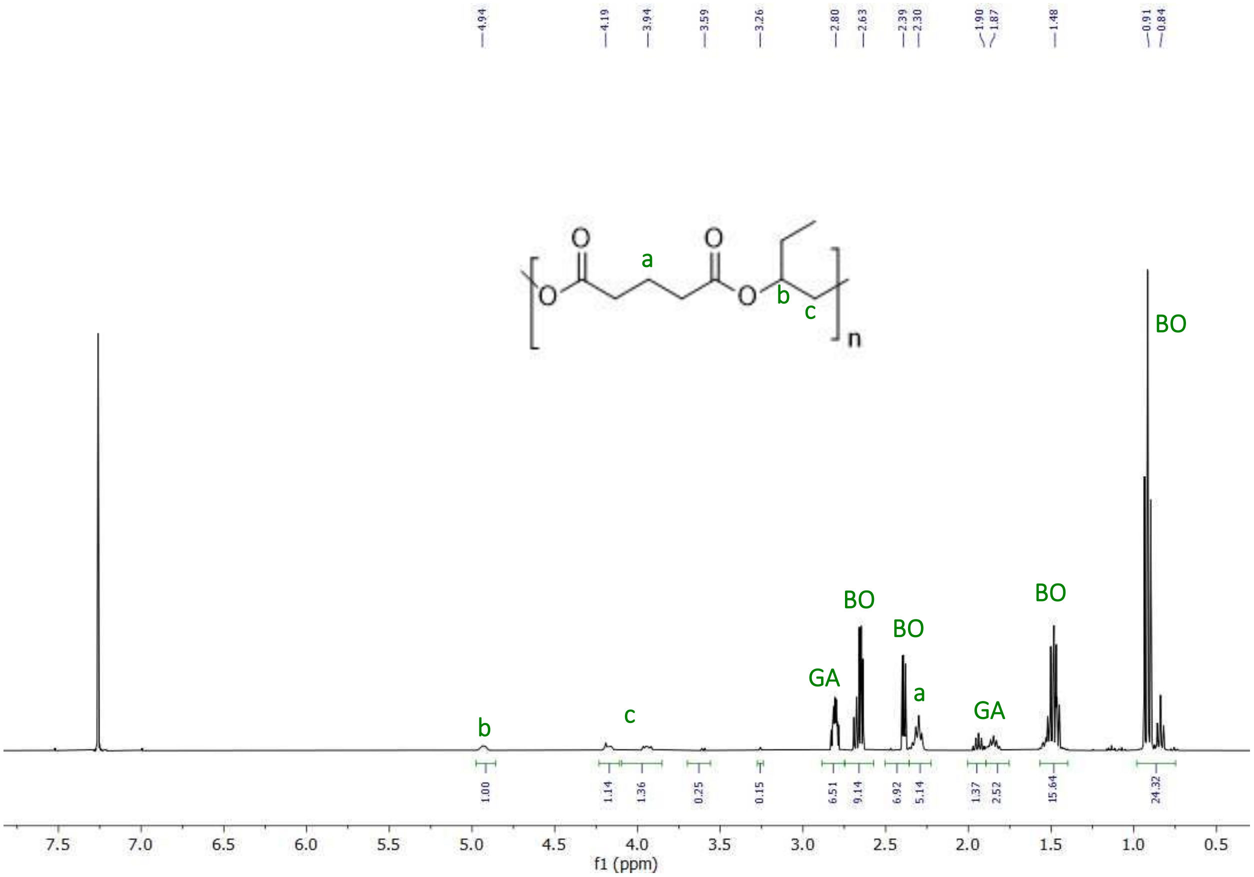

Figure S22. ^{1}H NMR spectrum of *in-situ* BO-*alt*-GA in $CDCl_3$ (**Table S7**, entry 3c).

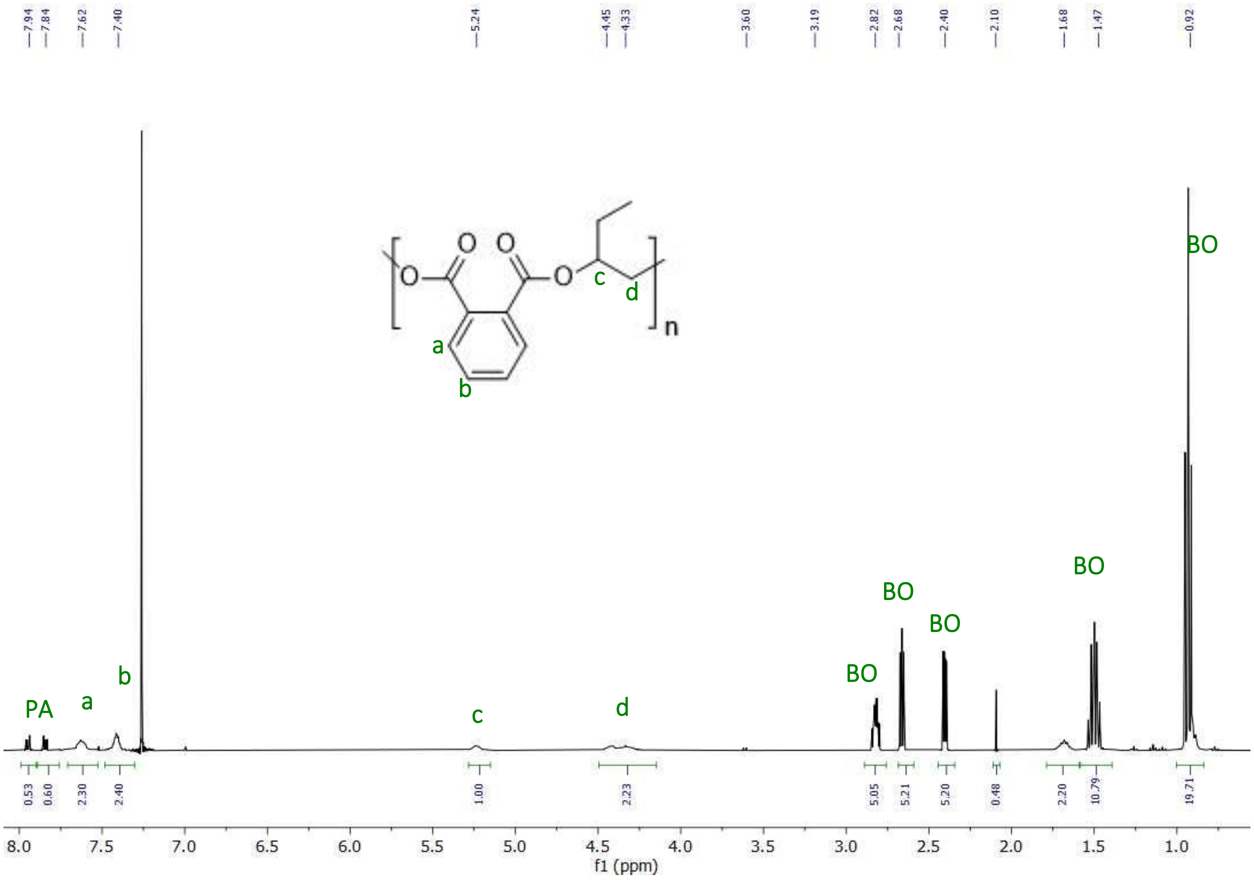

Figure S23. ^{1}H NMR spectrum of *in-situ* BO-*alt*-PA in $CDCl_3$ (**Table S8**, entry 2b).

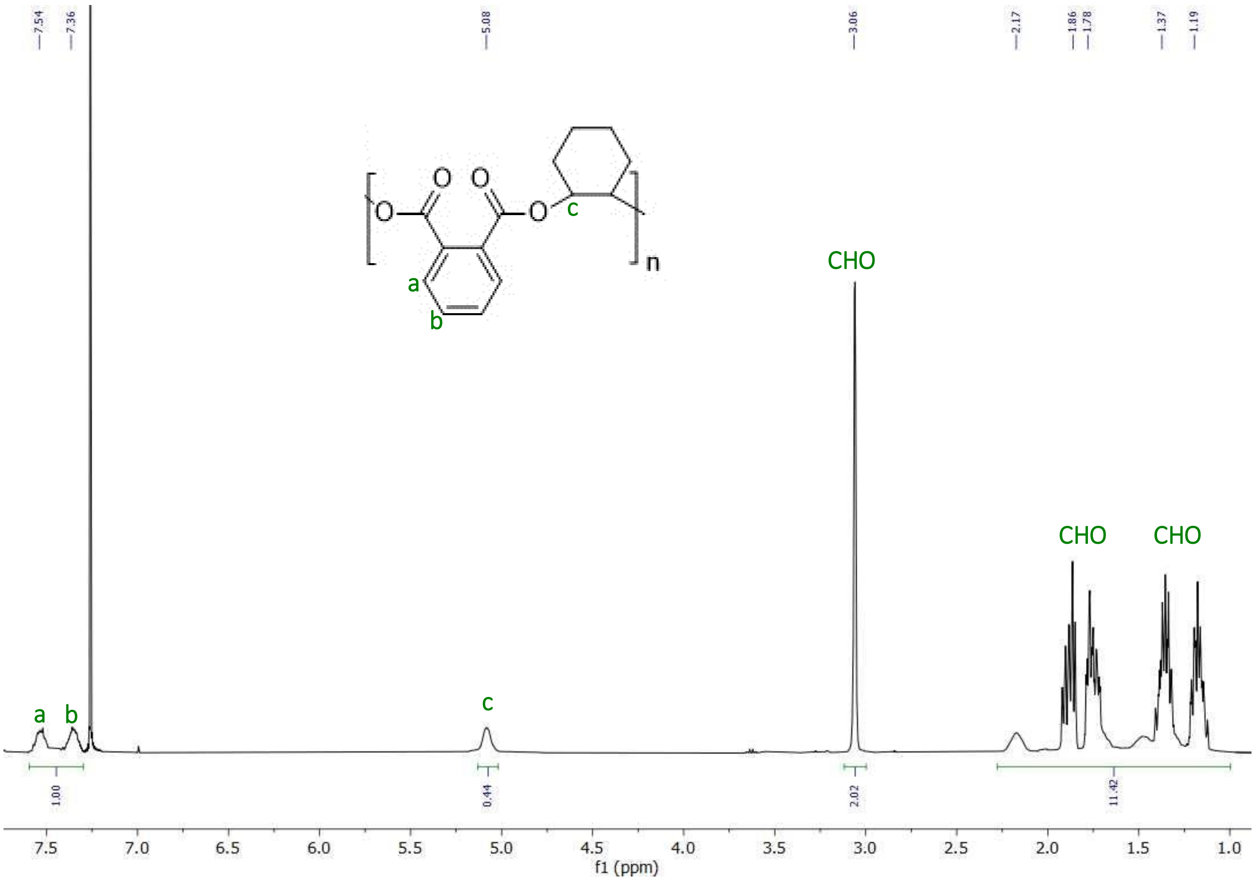

Figure S24. ^{1}H NMR spectrum of *in-situ* CHO-*alt*-PA in $CDCl_3$ (**Table S8**, entry 5a).

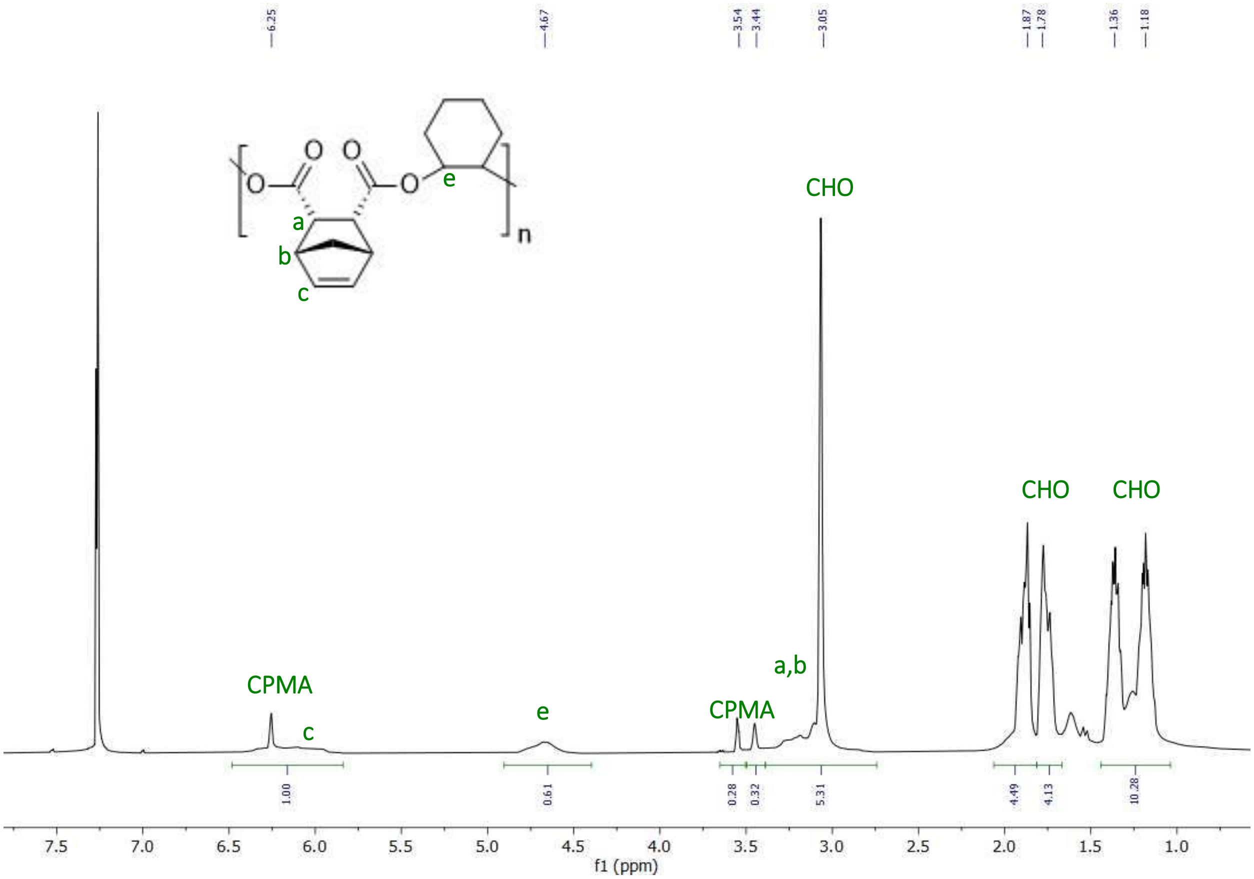

Figure S25. ^{1}H NMR spectrum of *in-situ* CHO-*alt*-CPMA in $CDCl_3$ (**Table S9**, entry 4a).

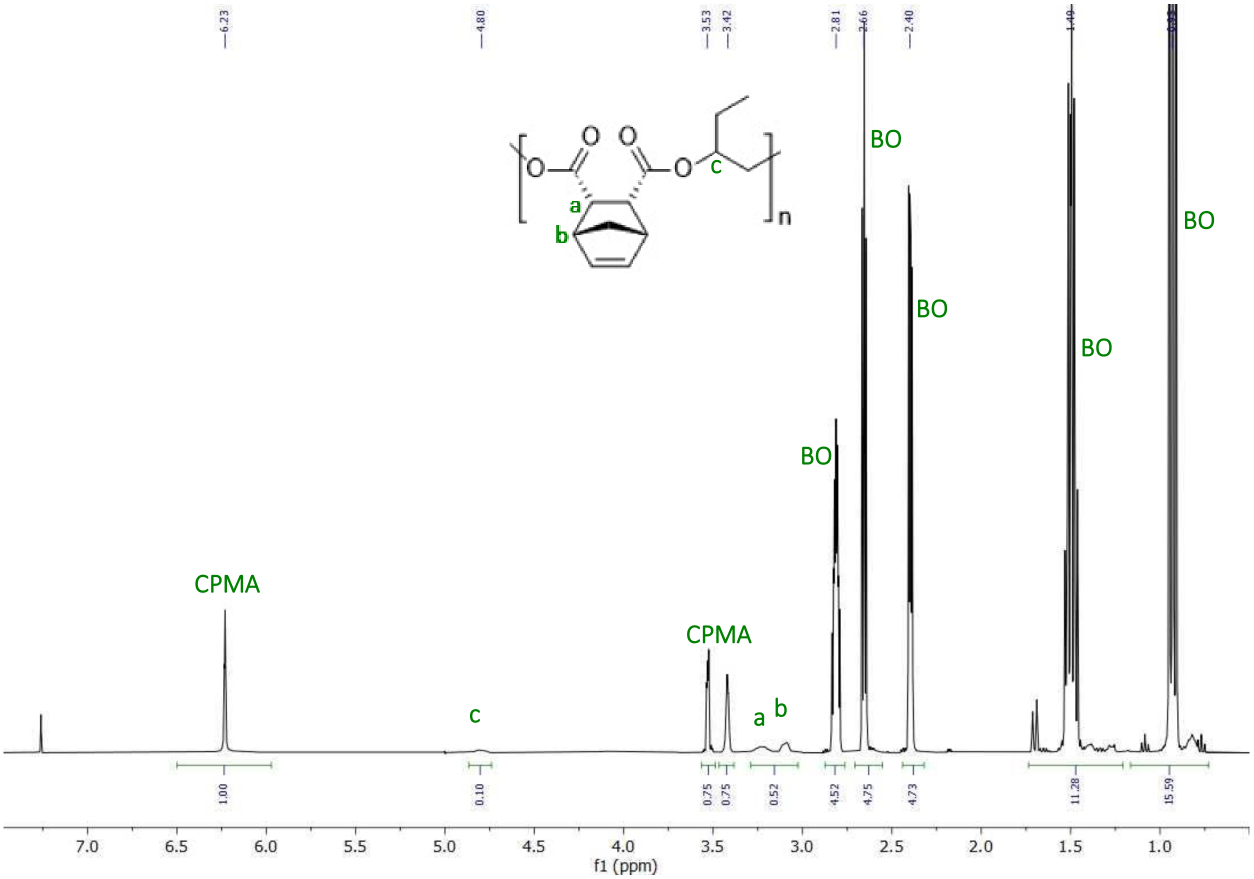

Figure S26. ^{1}H NMR spectrum of *in-situ* BO-*alt*-CPMA in $CDCl_3$ (**Table S10**, entry 1a).

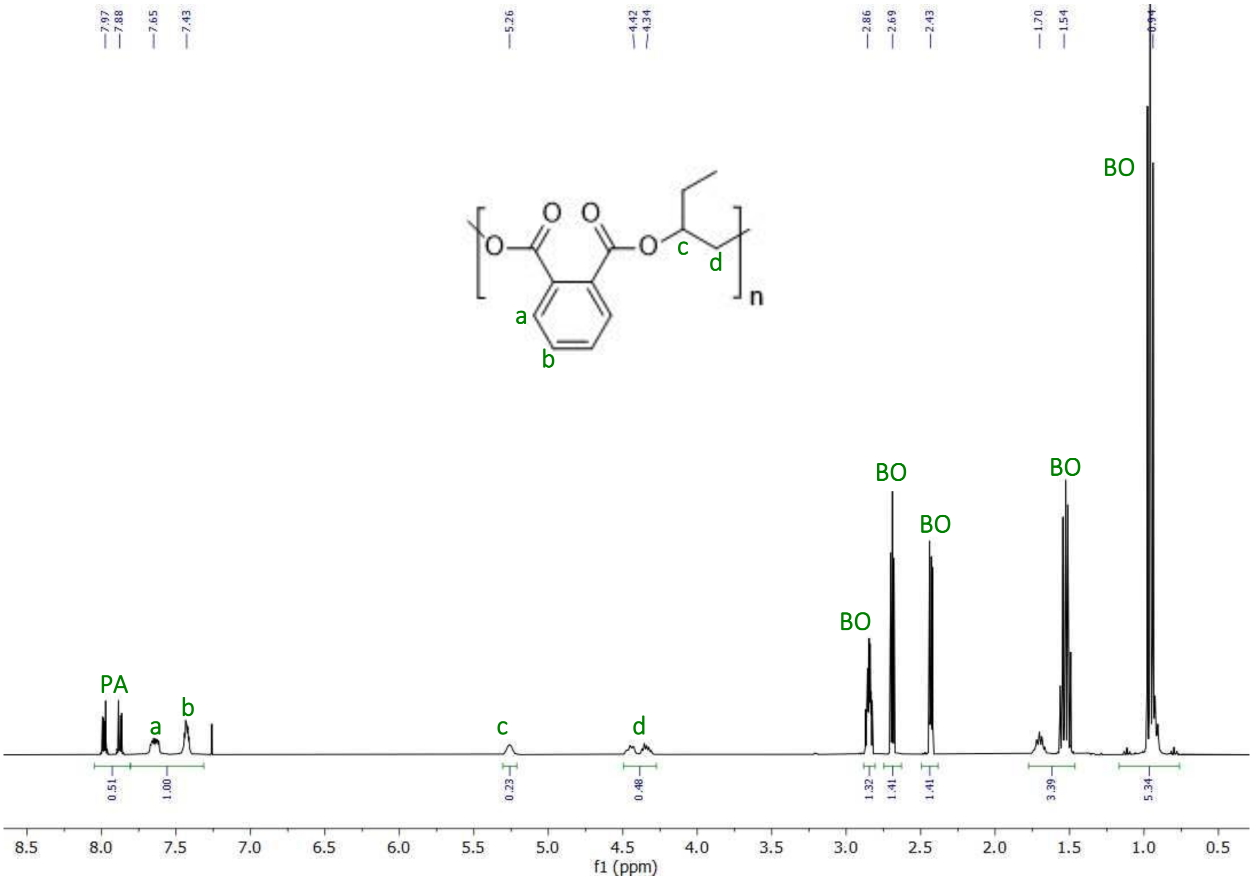

Figure S27. ^{1}H NMR spectrum of *in-situ* BO-*alt*-PA in $CDCl_3$ (**Table S10**, entry 2b).

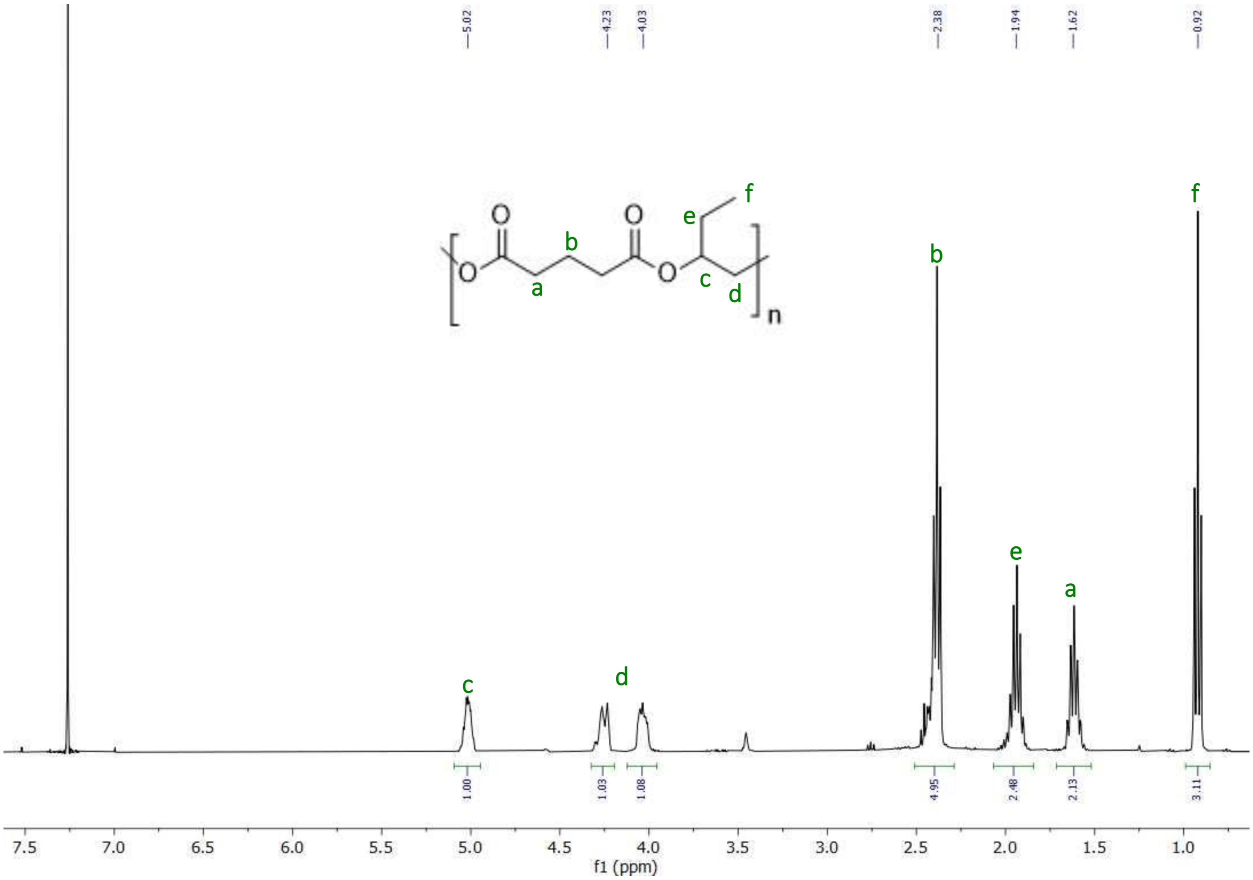

Figure S28. ^{1}H NMR spectrum of isolated BO-*alt*-GA in $CDCl_3$ (**Table S10**, entry 3b).

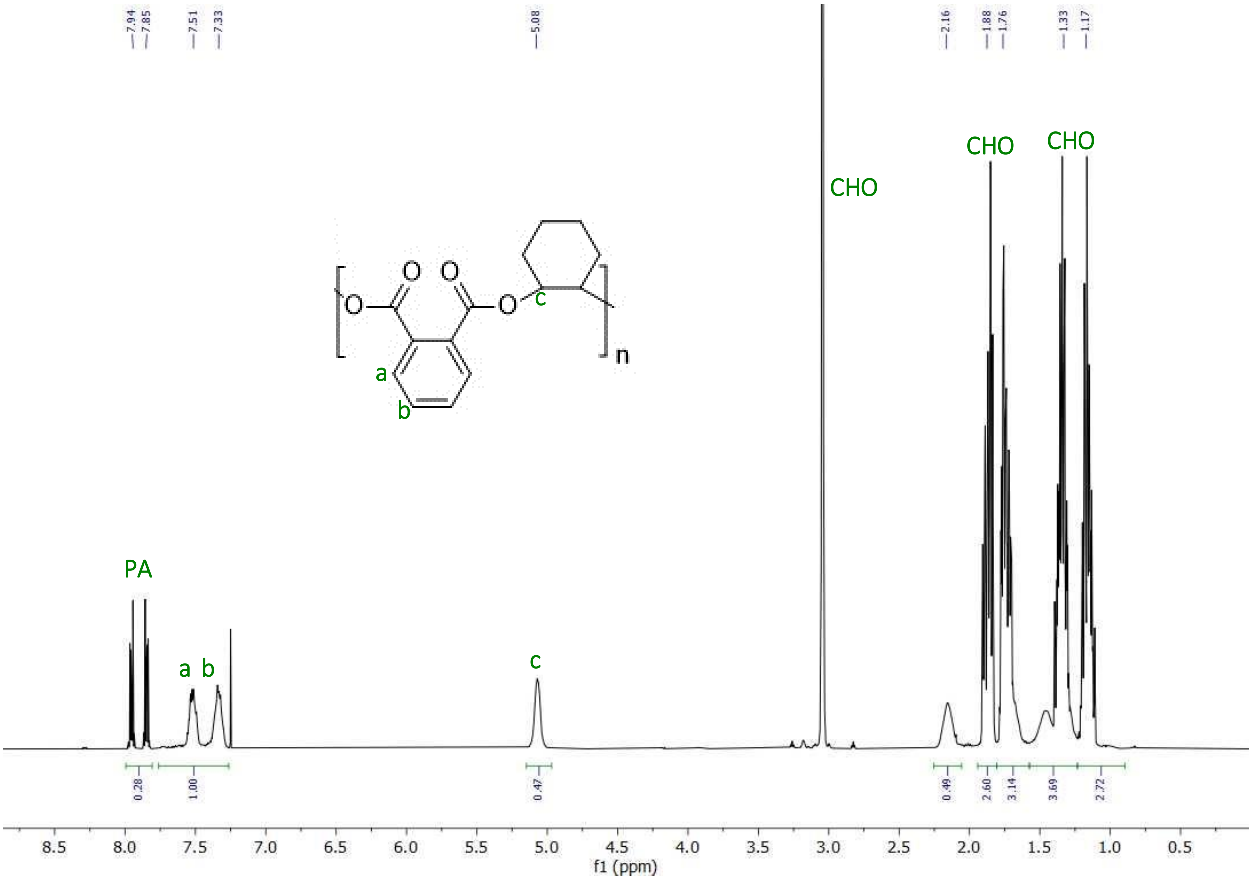

Figure S29. ^{1}H NMR spectrum of *in-situ* CHO-*alt*-PA in $CDCl_3$ (**Table S10**, entry 5b).

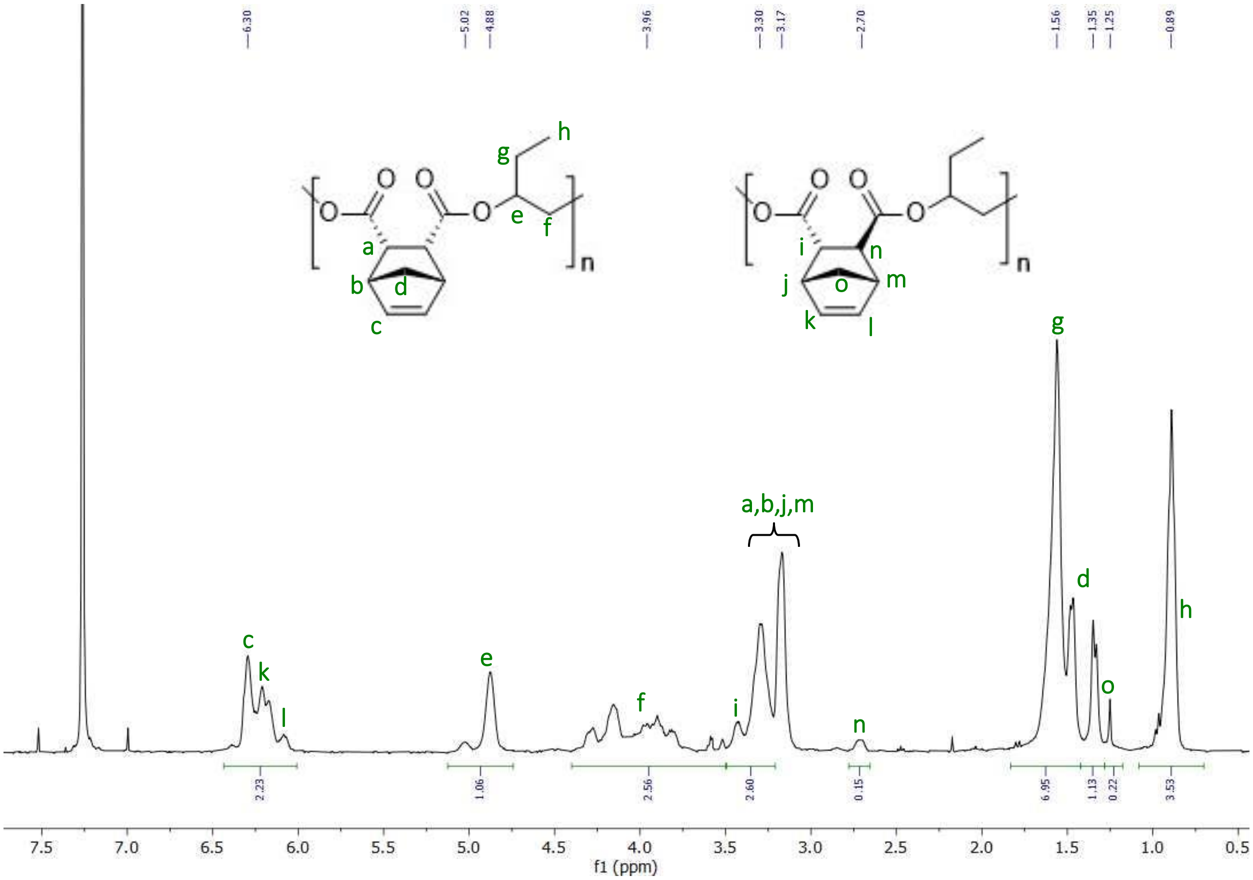

Figure S30. ^{1}H NMR spectrum of isolated BO-*alt*-CPMA in $CDCl_3$ (**Table S11**, entry 1a).

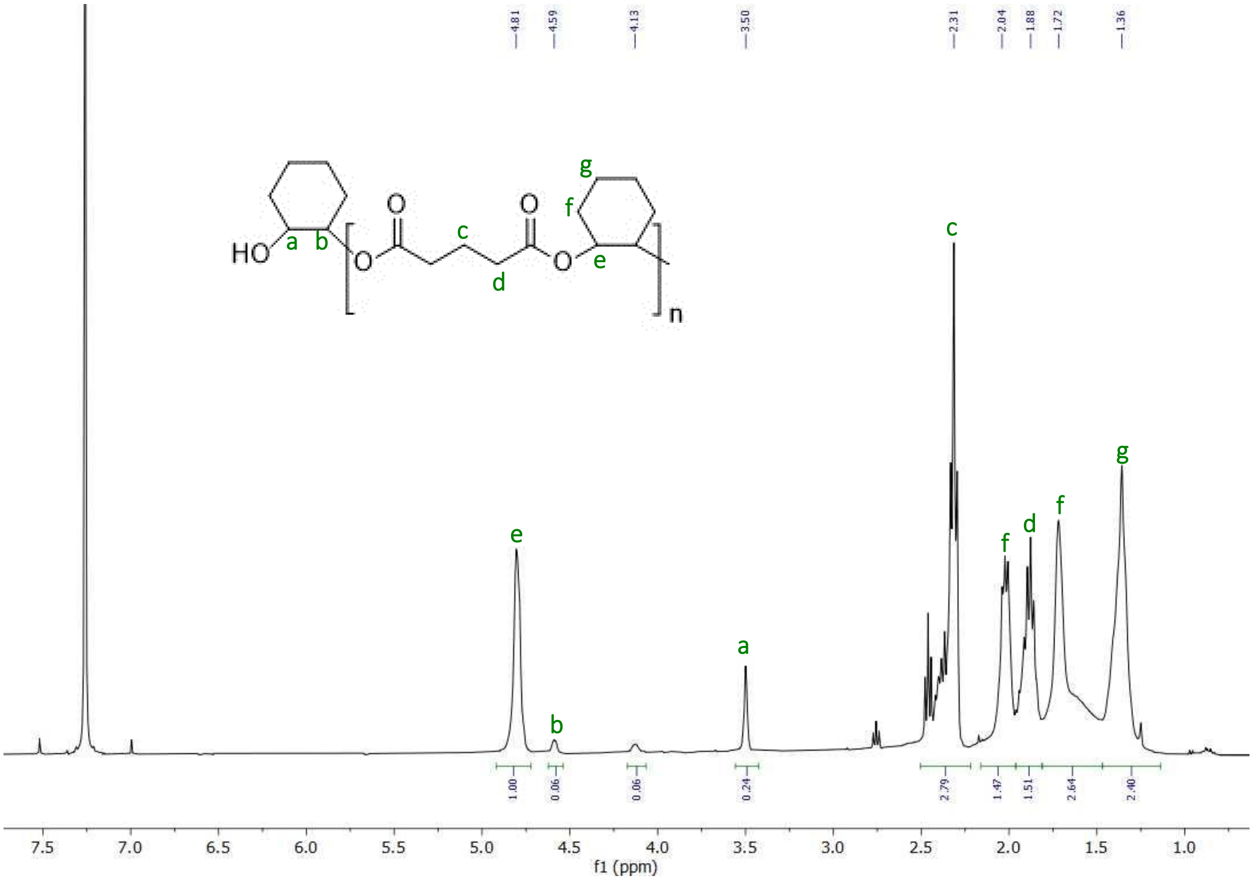

Figure S31. ^{1}H NMR spectrum of isolated CHO-*alt*-GA in $CDCl_3$ (**Table S11**, entry 6c).

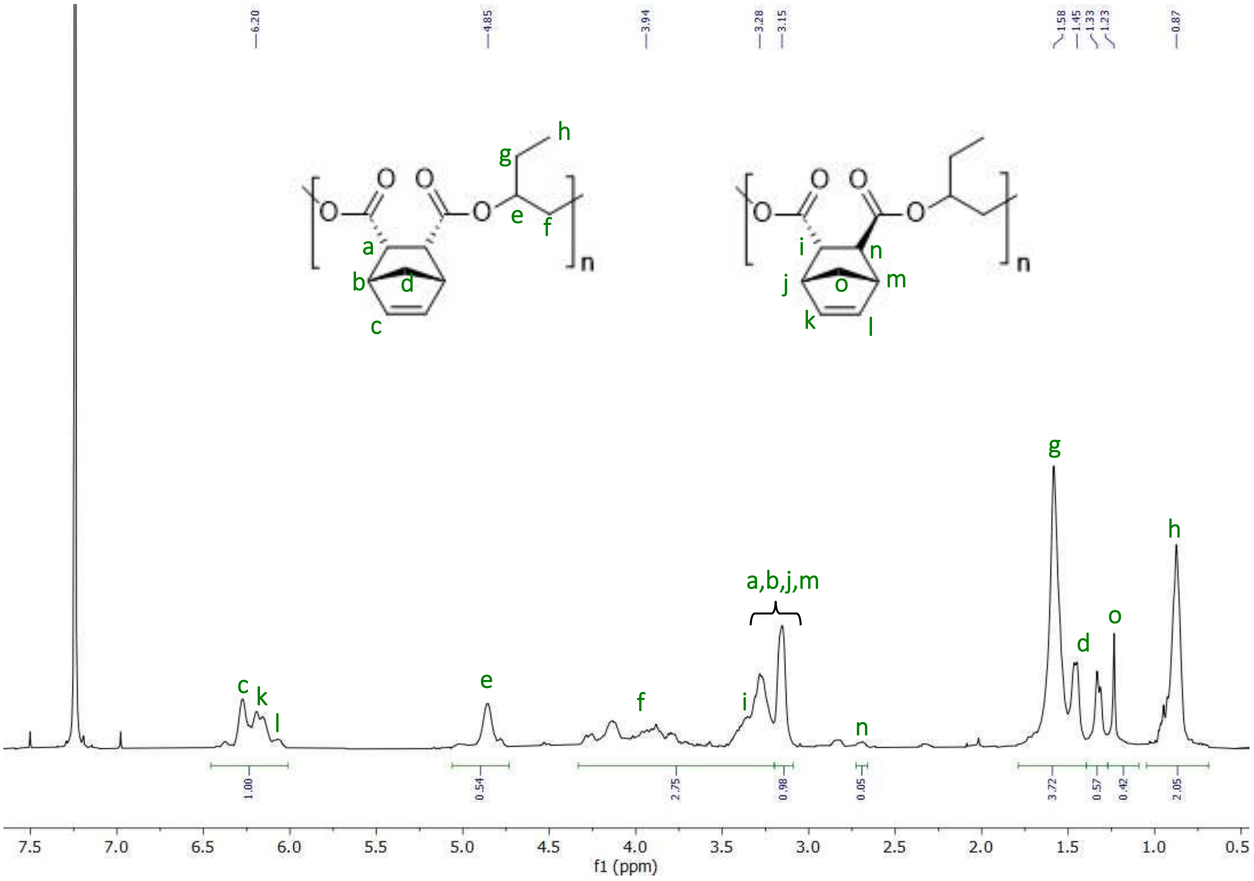

Figure S32. ^{1}H NMR spectrum of isolated BO-*alt*-CPMA in $CDCl_3$ (**Table S12**, entry 1a).

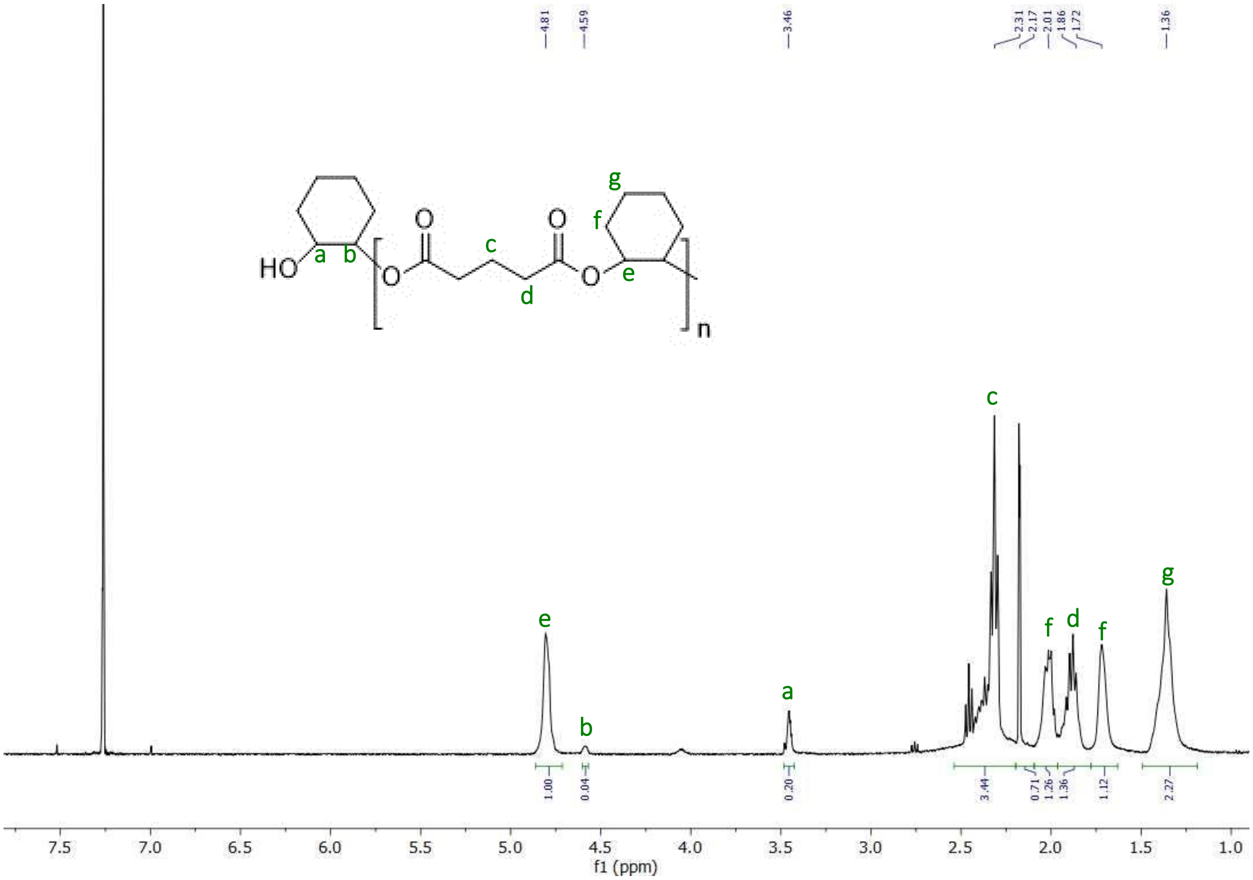

Figure S33. ^{1}H NMR spectrum of isolated CHO-*alt*-GA in $CDCl_3$ (**Table S12**, entry 6a).

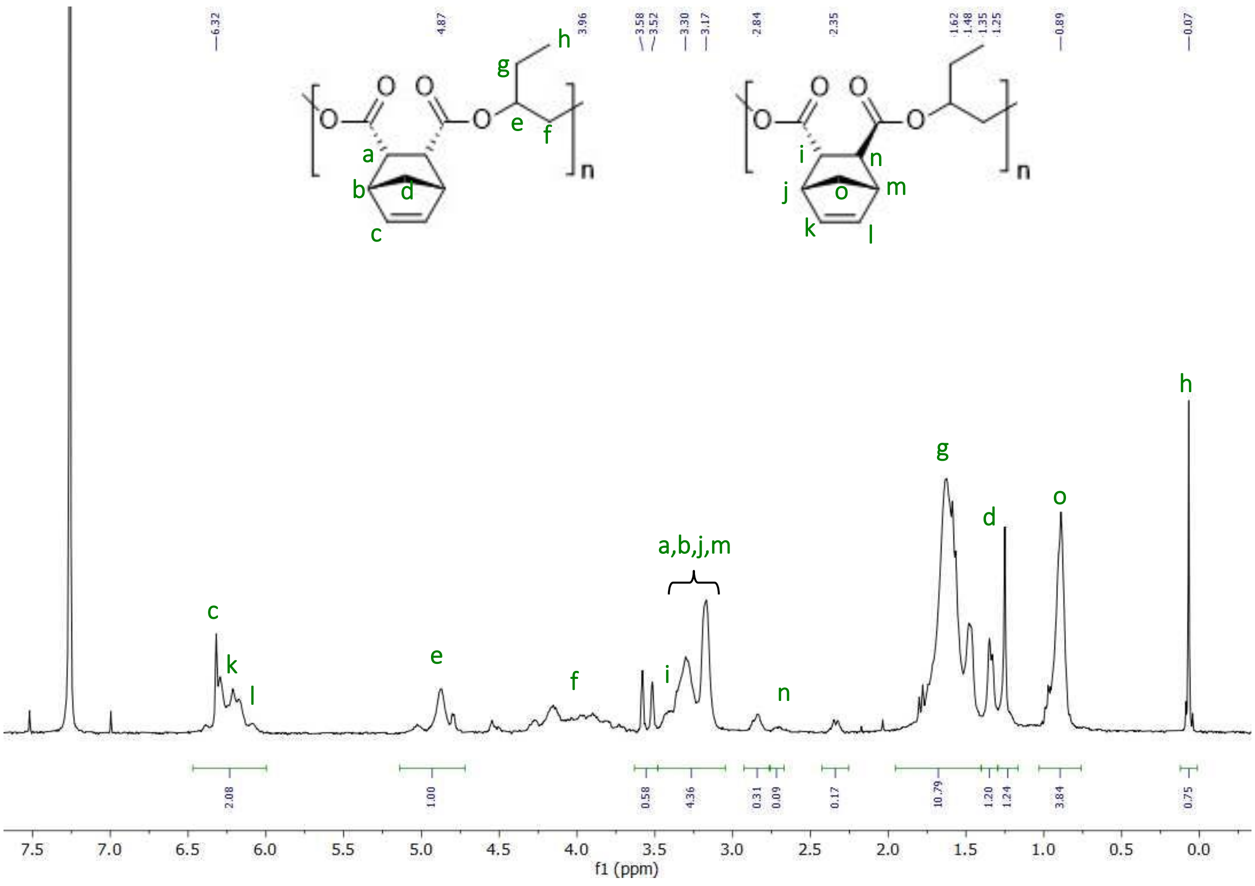

Figure S34. ^{1}H NMR spectrum of isolated BO-*alt*-CPMA in $CDCl_3$ (**Table S13**, entry 1b).

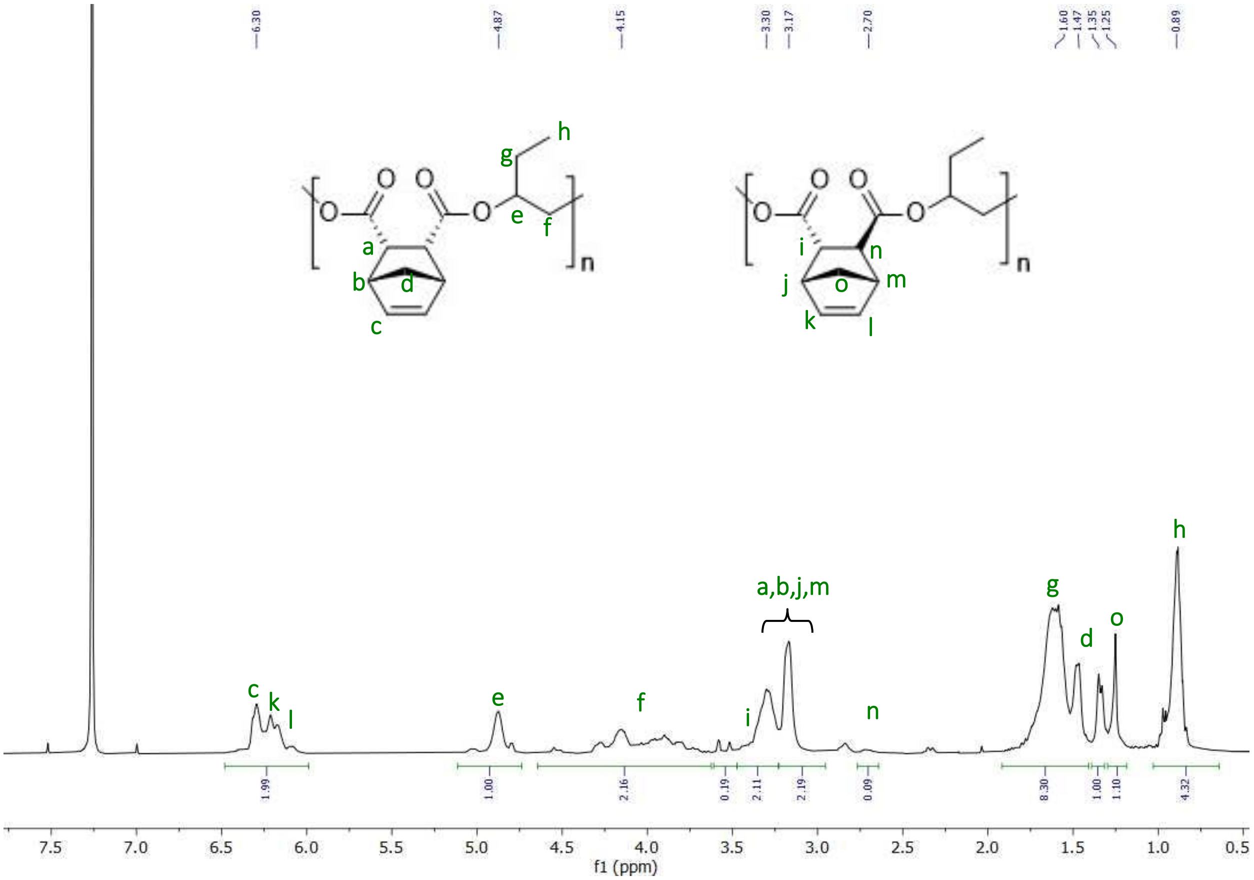

Figure S35. ^{1}H NMR spectrum of isolated BO-*alt*-CPMA in $CDCl_3$ (**Table S13**, entry 1e).

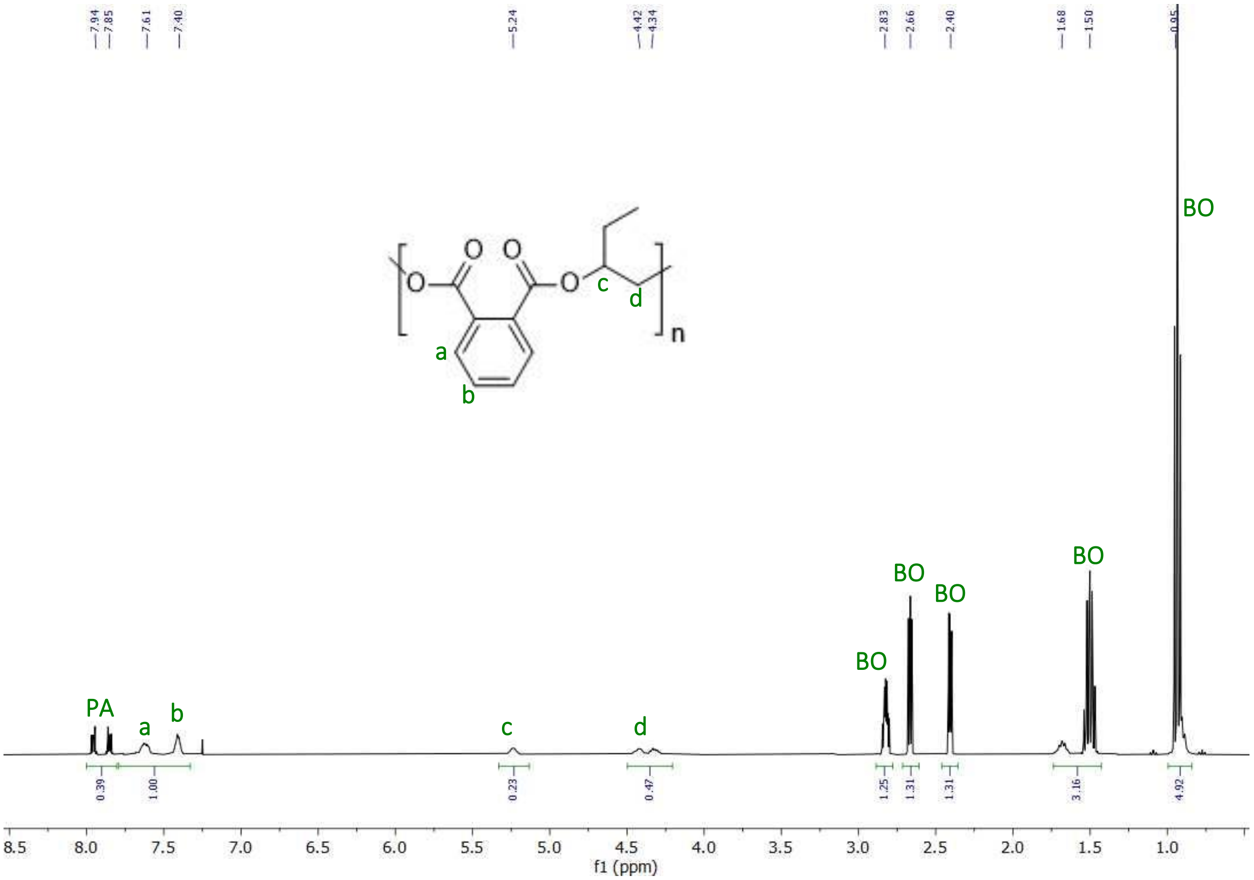

Figure S36. ^{1}H NMR spectrum of *in-situ* BO-*alt*-PA in $CDCl_3$ (**Table S13**, entry 2b).

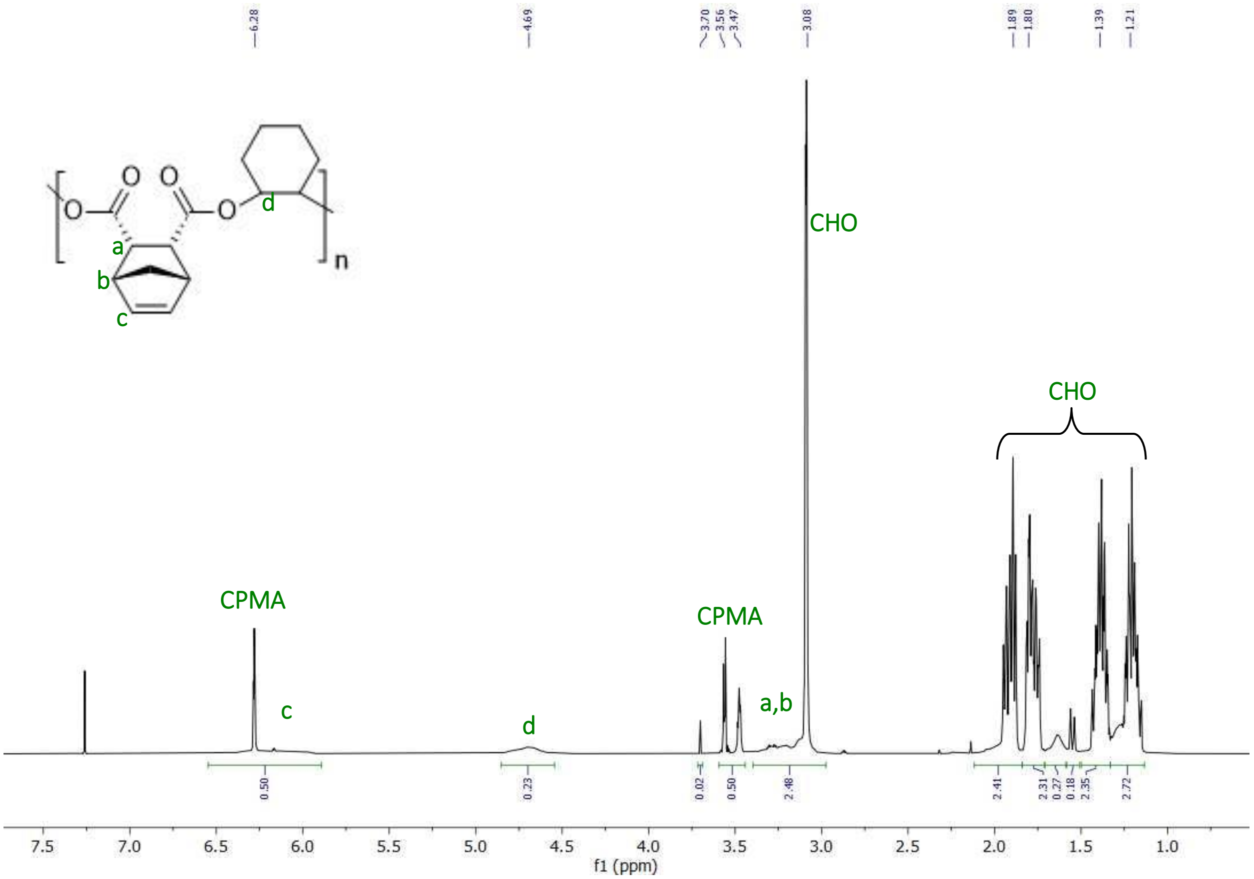

Figure S37. ^{1}H NMR spectrum of *in-situ* CHO-*alt*-CPMA in $CDCl_3$ (**Table S13**, entry 4a).

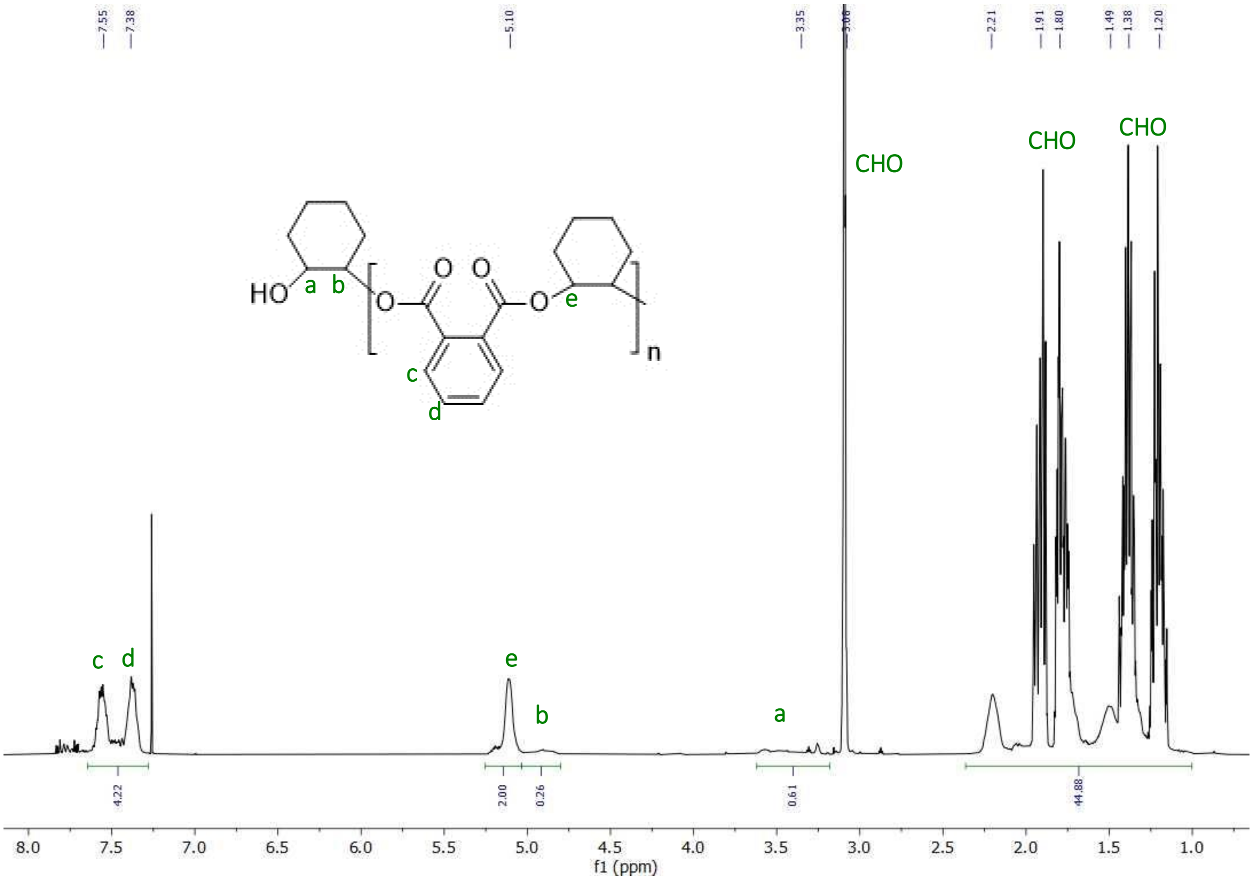

Figure S38. ^{1}H NMR spectrum of *in-situ* CHO-*alt*-PA in $CDCl_3$ (**Table S13**, entry 5b).

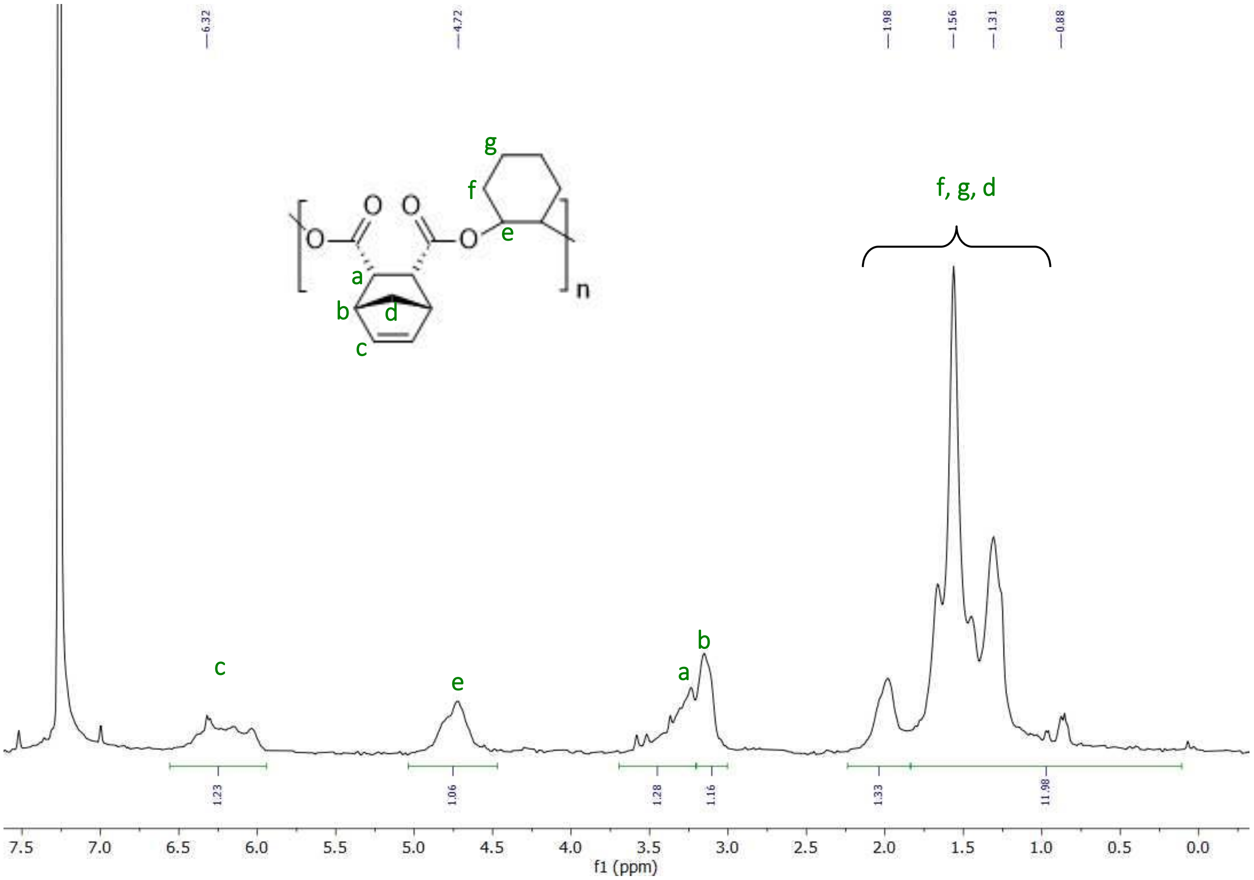

Figure S39. ^{1}H NMR spectrum of isolated CHO-*alt*-CPMA in $CDCl_3$ (**Table S14**, entry 4a).

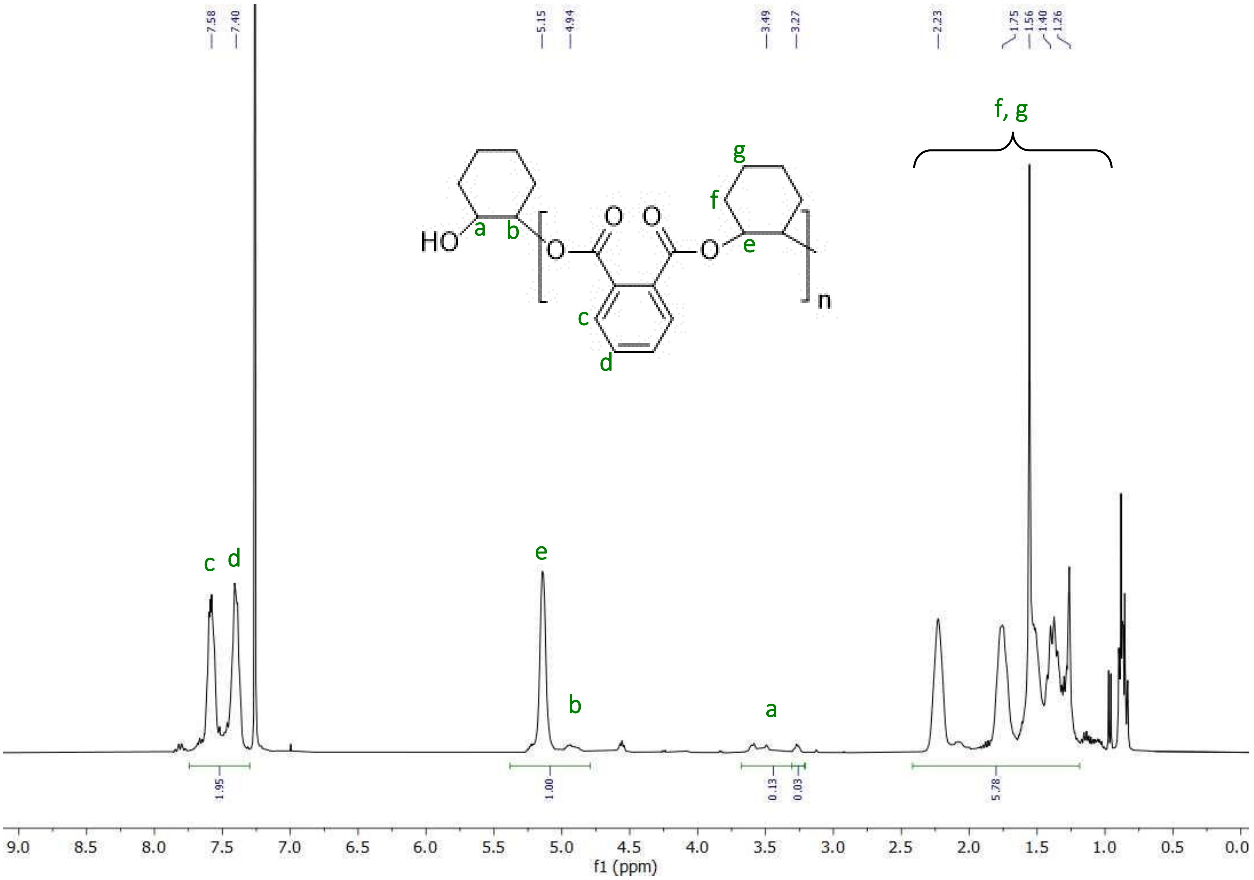

Figure S40. ^{1}H NMR spectrum of isolated CHO-*alt*-PA in $CDCl_3$ (**Table S14**, entry 5c).

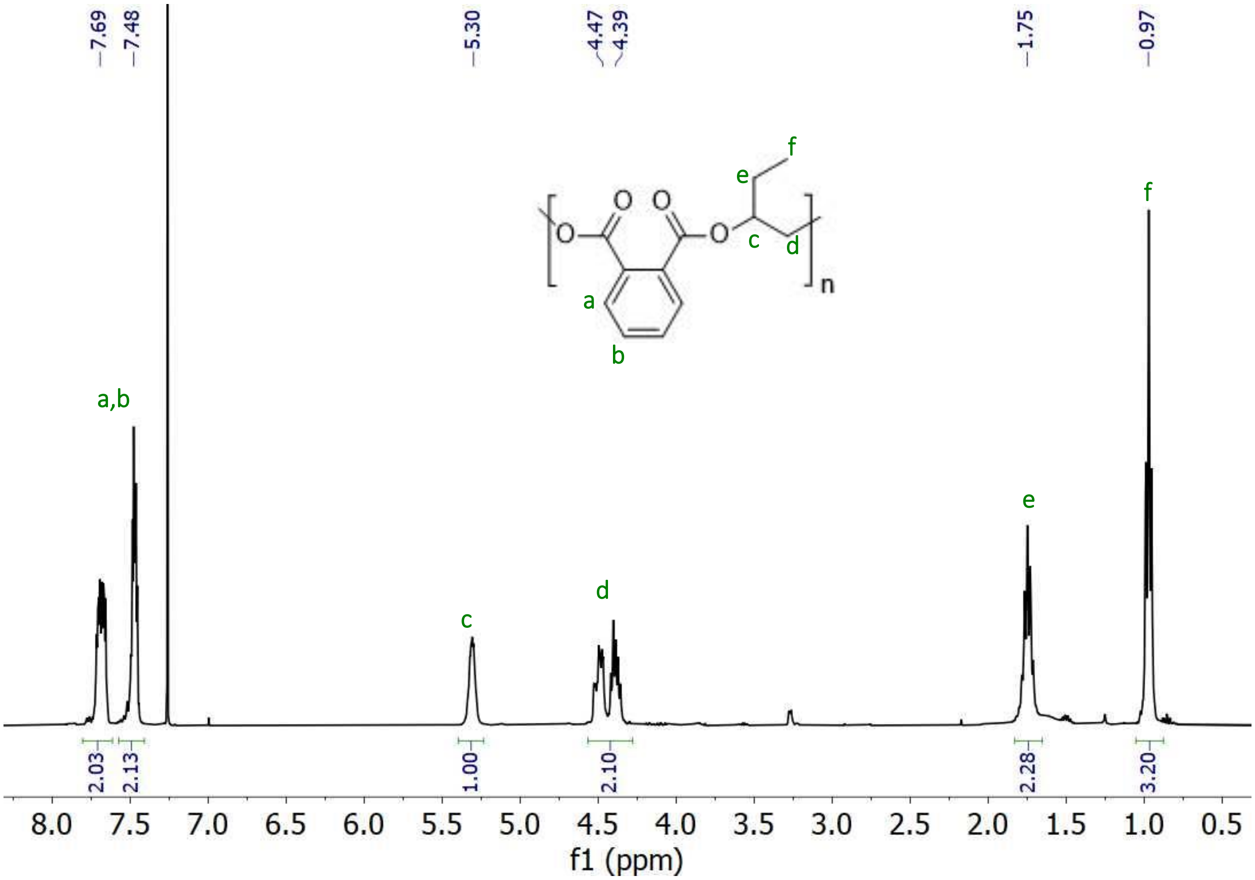

Figure S41. ^{1}H NMR spectrum of isolated BO-*alt*-PA in $CDCl_3$ (**Table S16**, entry 2c).

5. GPC spectra

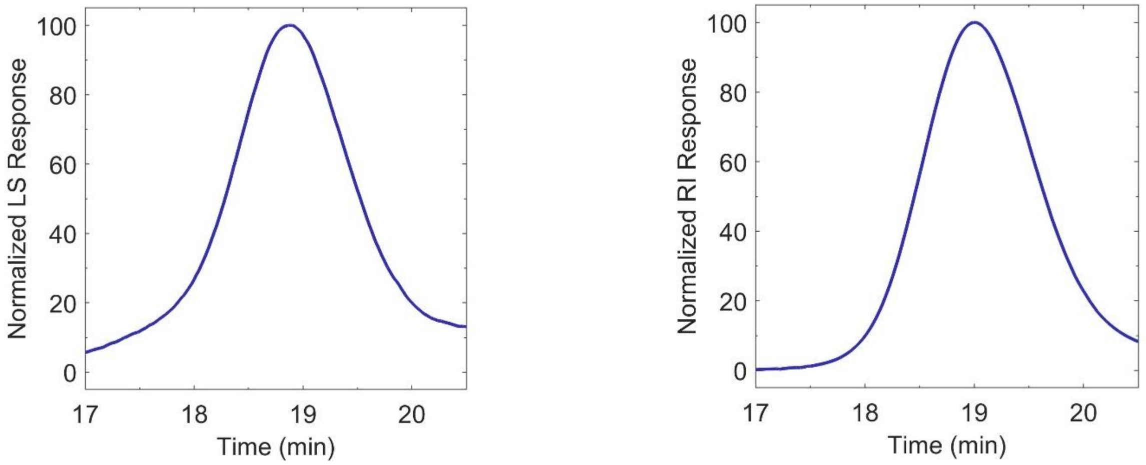

Figure S42. GPC trace of **Table S1**, entry 1b and **Table 2**, entry 1 (LS on the left, RI on the right). BO-*alt*-CPMA from ChCl exposed to air.

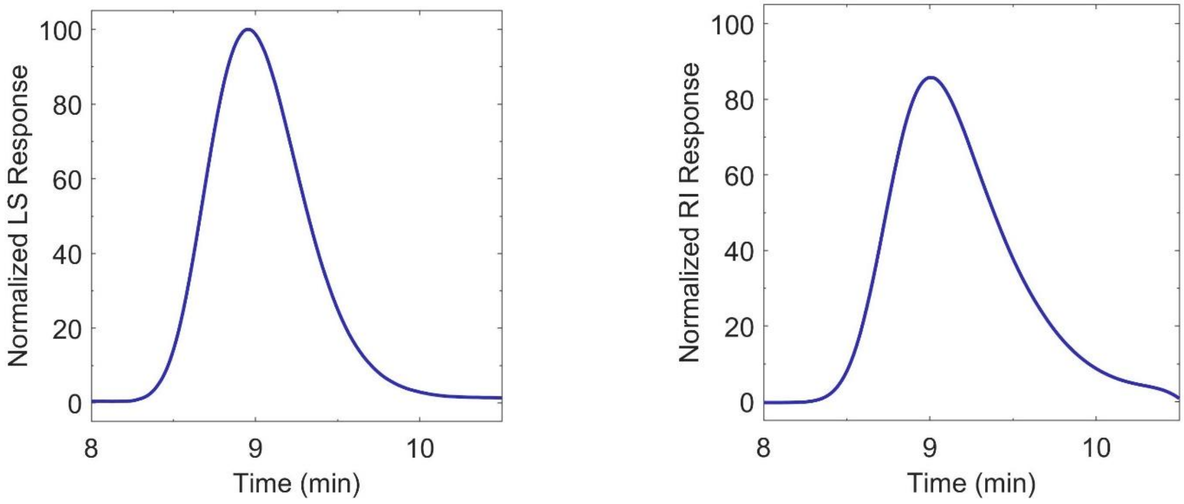

Figure S43. GPC trace of **Table S1**, entry 2a (LS on the left, RI on the right). BO-*alt*-PA from ChCl exposed to air. This sample was processed using one Agilent PolyPore column instead of the standard two in series.

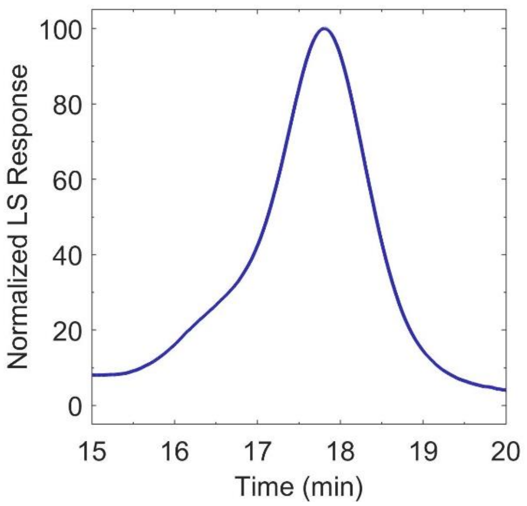

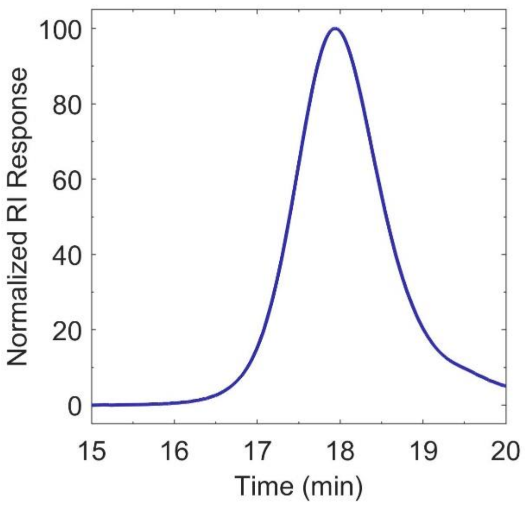

Figure S44. GPC trace of **Table S2**, entry 1a (LS on the left, RI on the right). BO-*alt*-CPMA from ChBr exposed to air.

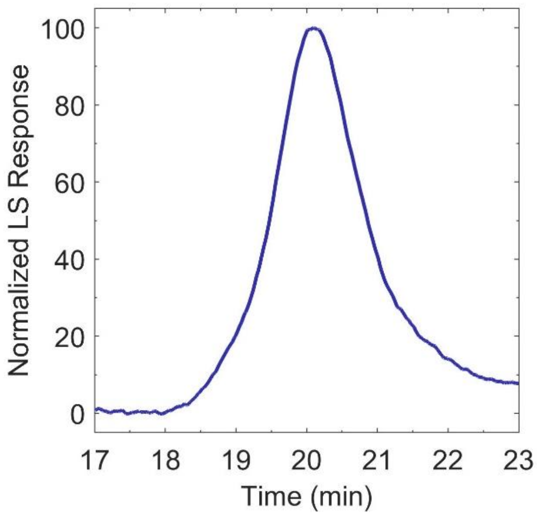

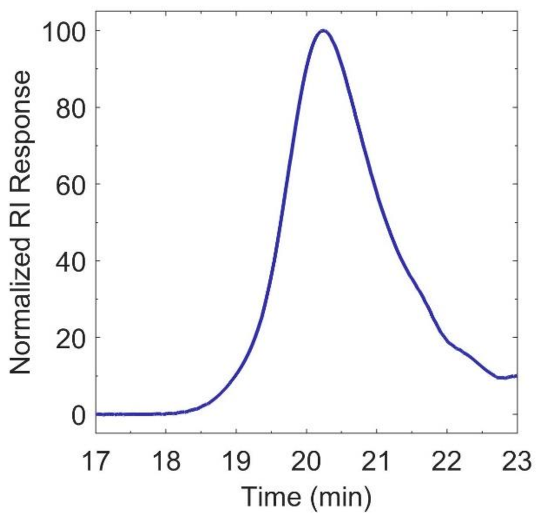

Figure S45. GPC trace of **Table S2**, entry 3a (LS on the left, RI on the right). BO-*alt*-GA from ChBr exposed to air.

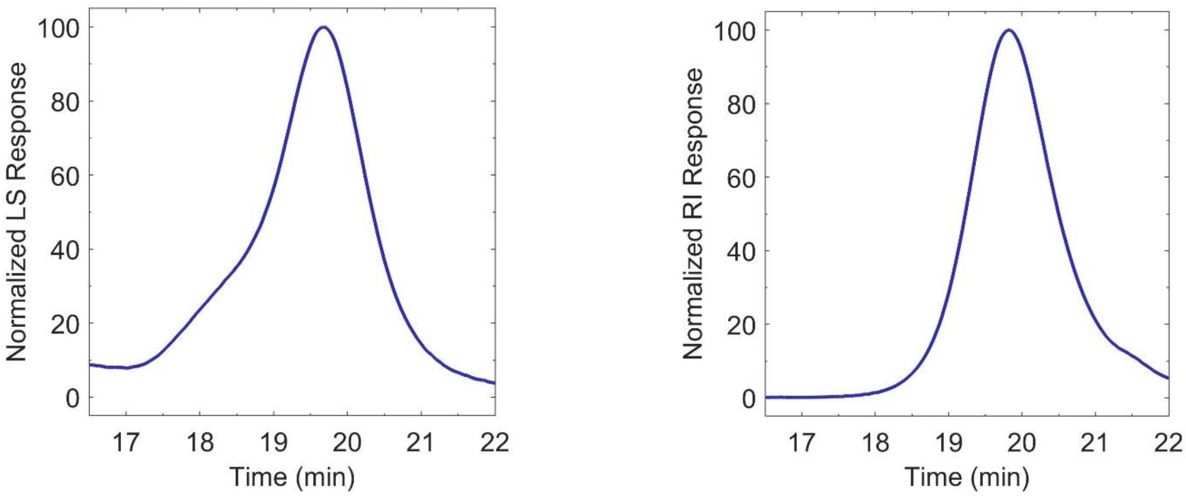

Figure S46. GPC trace of **Table S3**, entry 1a (LS on the left, RI on the right). BO-*alt*-CPMA from ChI exposed to air.

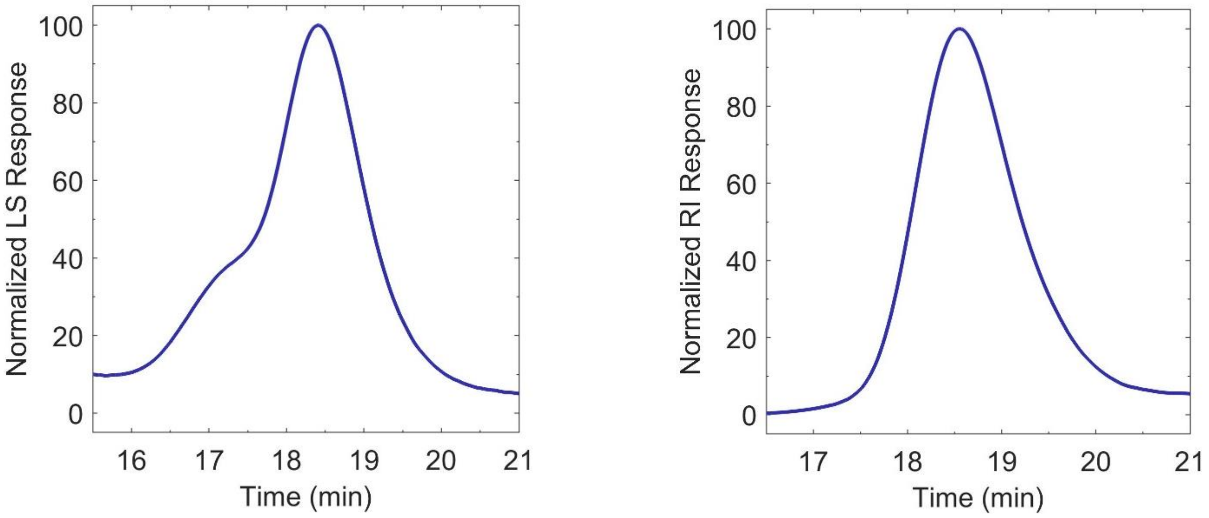

Figure S47. GPC trace of **Table S3**, entry 4a (LS on the left, RI on the right). CHO-*alt*-CPMA from ChI exposed to air.

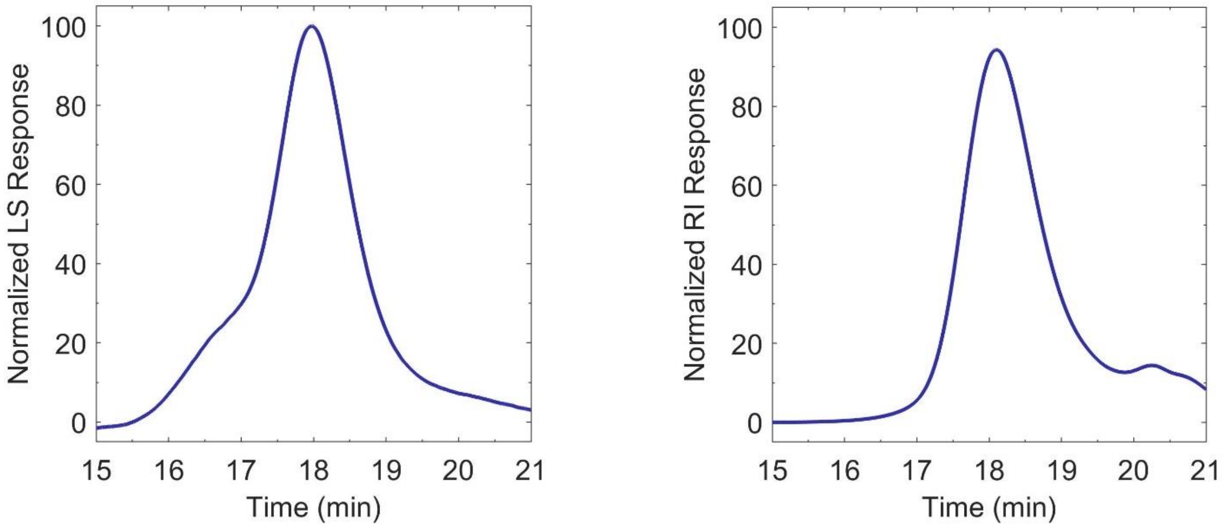

Figure S48. GPC trace of **Table S4**, entry 1c and **Table 2**, entry 2 (LS on the left, RI on the right). BO-*alt*-CPMA from ChCl/urea exposed to air.

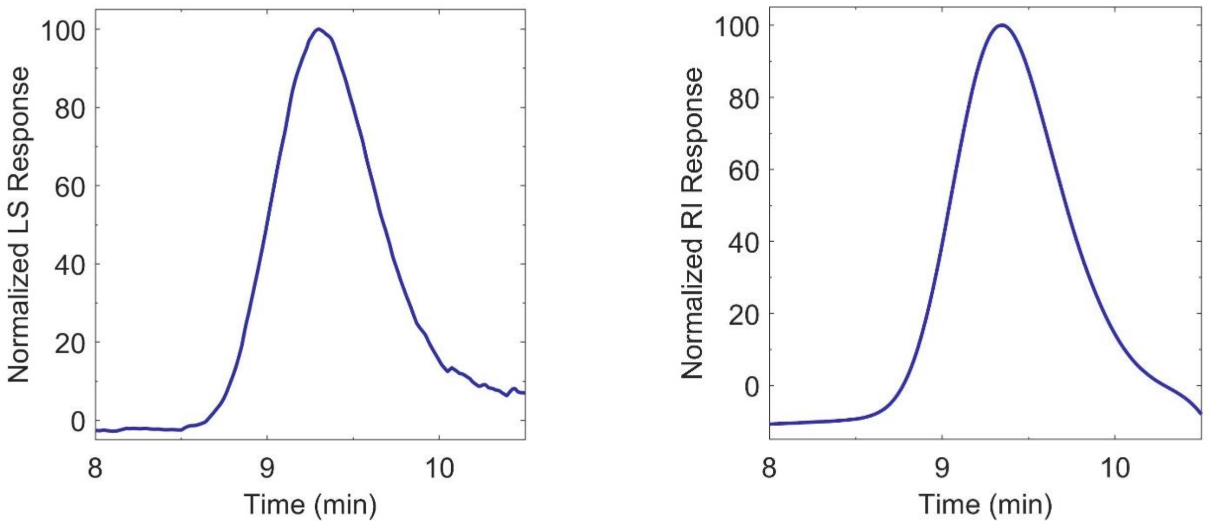

Figure S49. GPC trace of **Table S5**, entry 1a and **Table 2**, entry 3 (LS on the left, RI on the right). BO-*alt*-CPMA from ChCl/EG exposed to air. This sample was processed using one Agilent PolyPore column instead of the standard two in series.

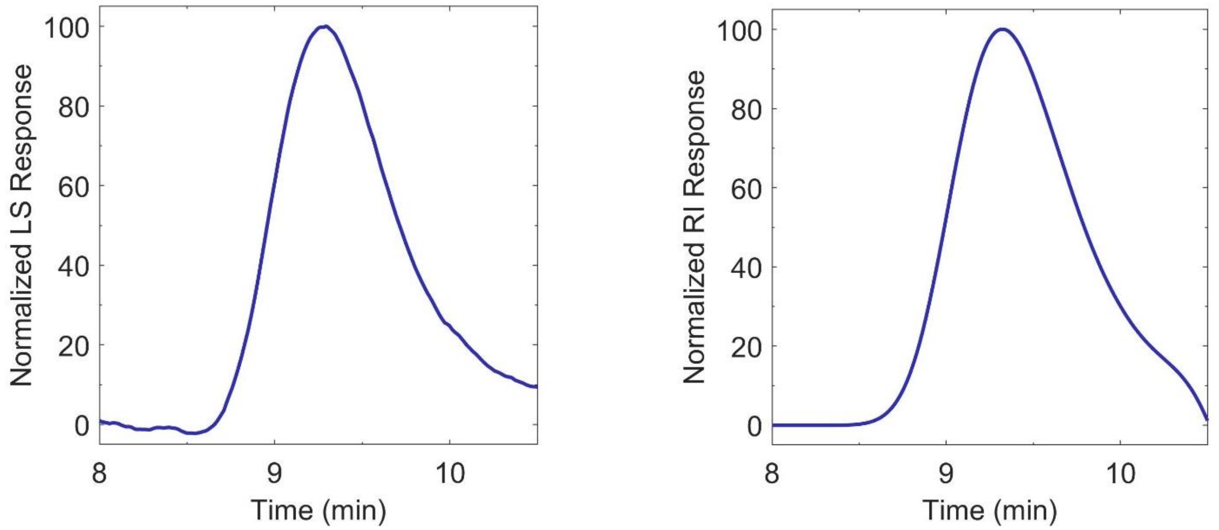

Figure S50. GPC trace of **Table S5**, entry 2a (LS on the left, RI on the right). BO-*alt*-PA from ChCl/EG exposed to air. This sample was processed using one Agilent PolyPore column instead of the standard two in series.

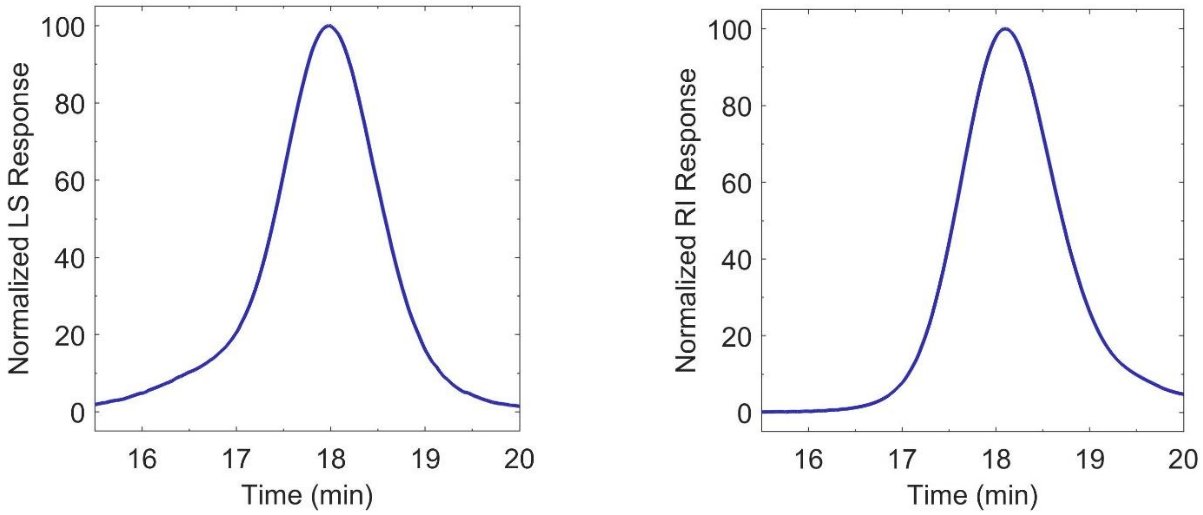

Figure S51. GPC trace of **Table S7**, entry 1a (LS on the left, RI on the right). BO-*alt*-CPMA from ChBr/EG exposed to air.

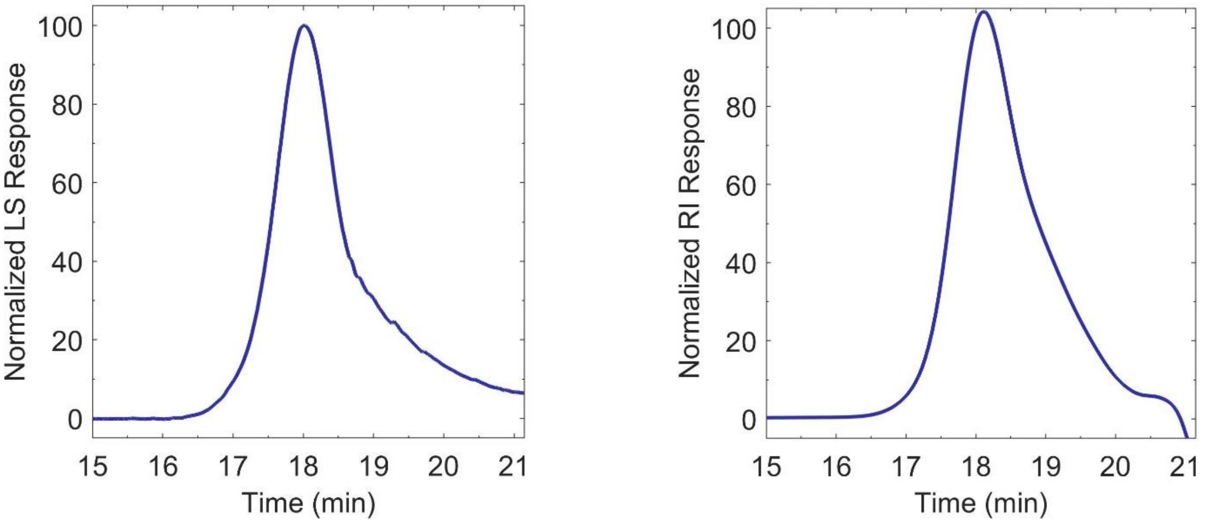

Figure S52. GPC trace of **Table S8**, entry 5a (LS on the left, RI on the right). CHO-*alt*-PA from ChI/urea exposed to air.

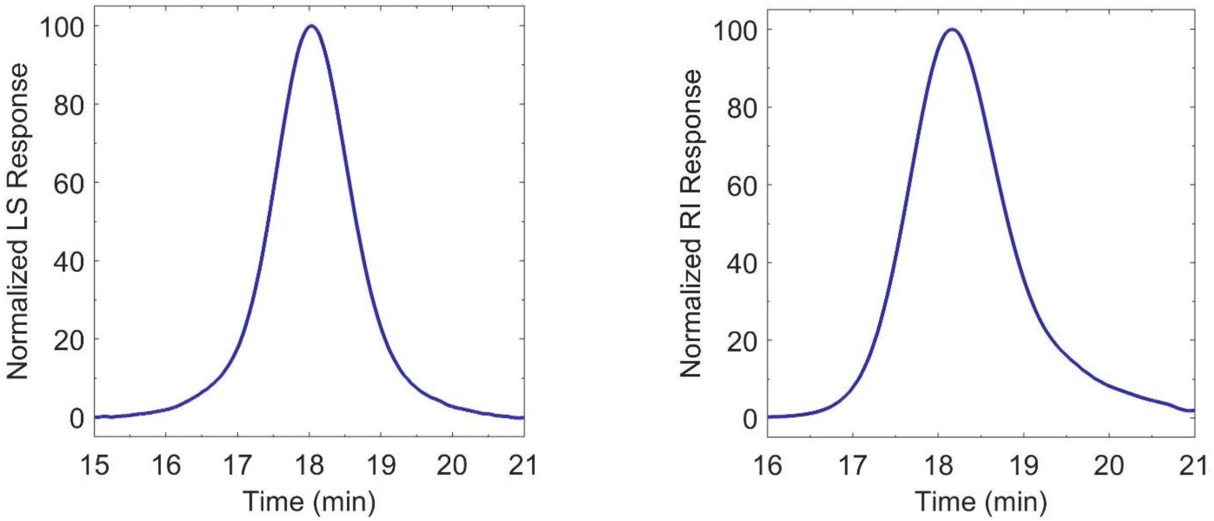

Figure S53. GPC trace of **Table S9**, entry 1a (LS on the left, RI on the right). BO-*alt*-CPMA from ChI/EG exposed to air.

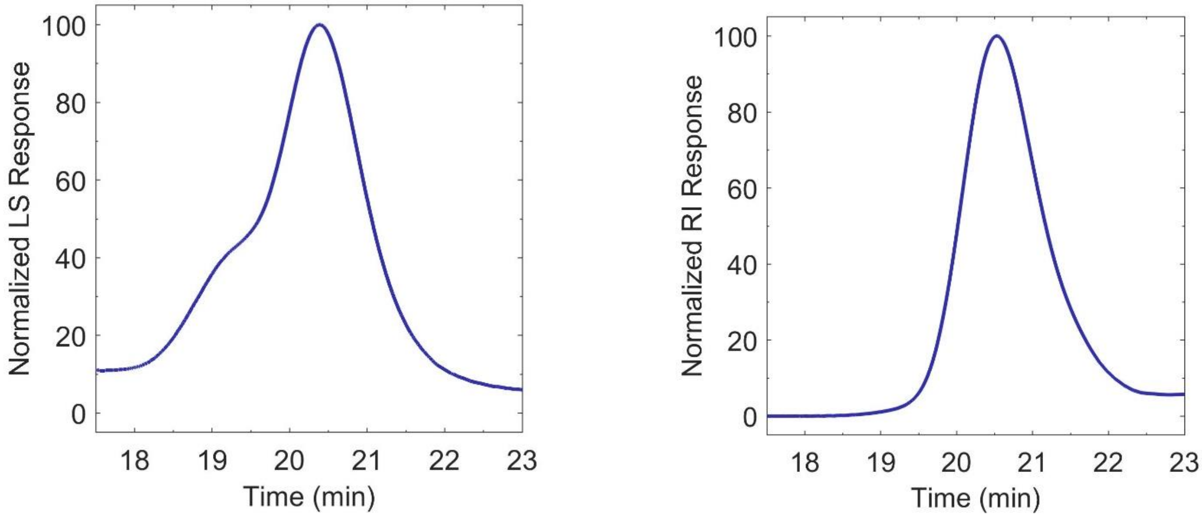

Figure S54. GPC trace of **Table S9**, entry 4a (LS on the left, RI on the right). CHO-*alt*-CPMA from ChI/EG exposed to air.

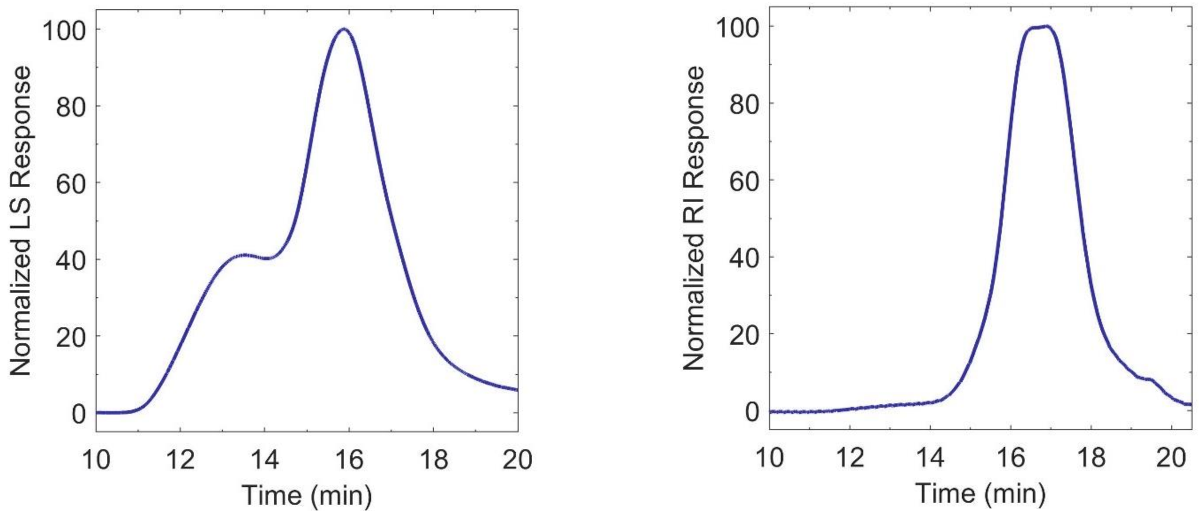

Figure S55. GPC trace of **Table S10**, entry 1d and **Table 2**, entry 4 (LS on the left, RI on the right). BO-*alt*-CPMA from ChCl air-free.

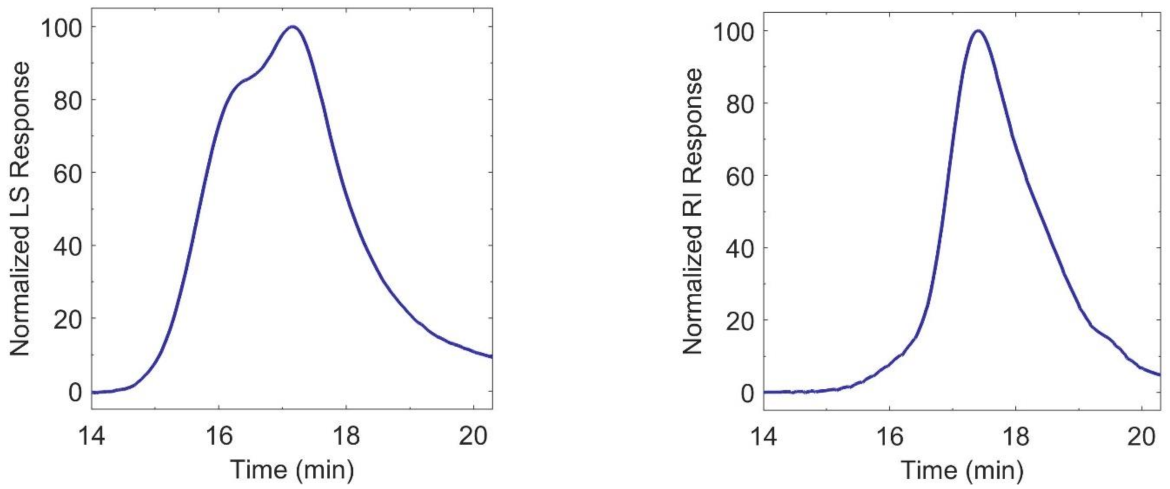

Figure S56. GPC trace of **Table S13**, entry 1b and **Table 2**, entry 5 (LS on the left, RI on the right). BO-*alt*-CPMA from ChCl/urea air-free.

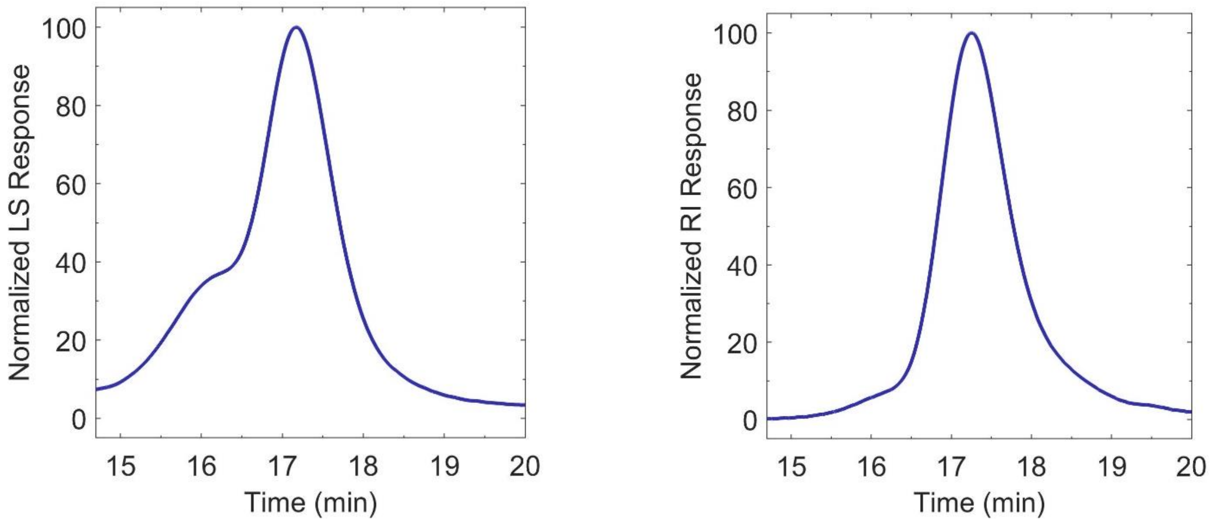

Figure S57. GPC trace of **Table S14**, entry 1b and **Table 2**, entry 6 (LS on the left, RI on the right). BO-*alt*-CPMA from ChCl/EG air-free.

6. TGA data

6.1 TGA spectra for the degradation of polymers

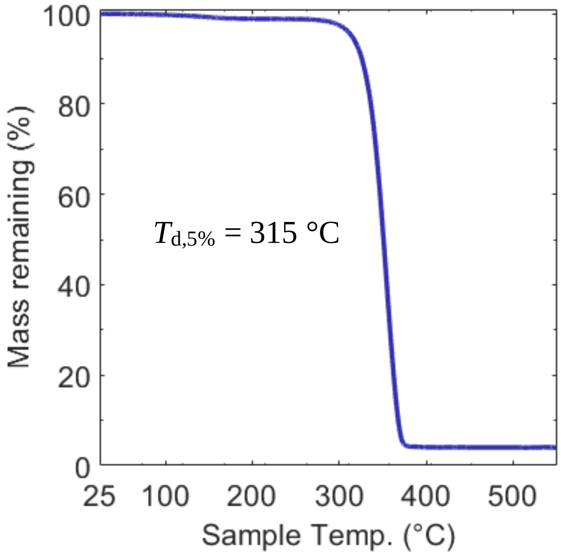

Figure S58. TGA spectrum of BO-*alt*-PA (**Table S10**, entry 2f) using air-free ChCl heated from 25 °C – 550 °C.

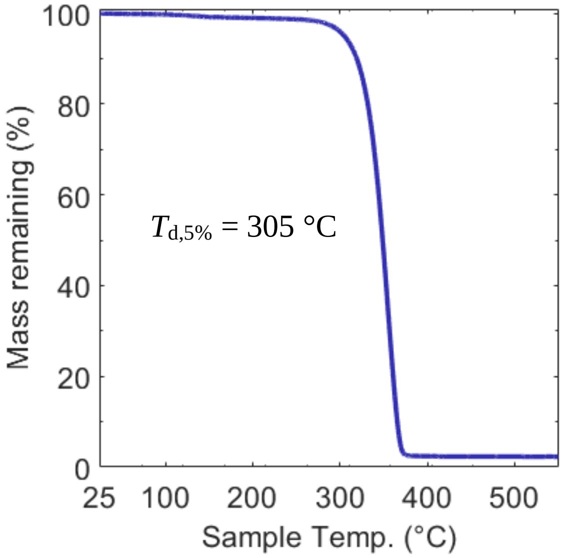

Figure S59. TGA spectrum of BO-*alt*-PA (**Table S13**, entry 2d) using air-free ChCl/urea heated from 25 °C – 550 °C.

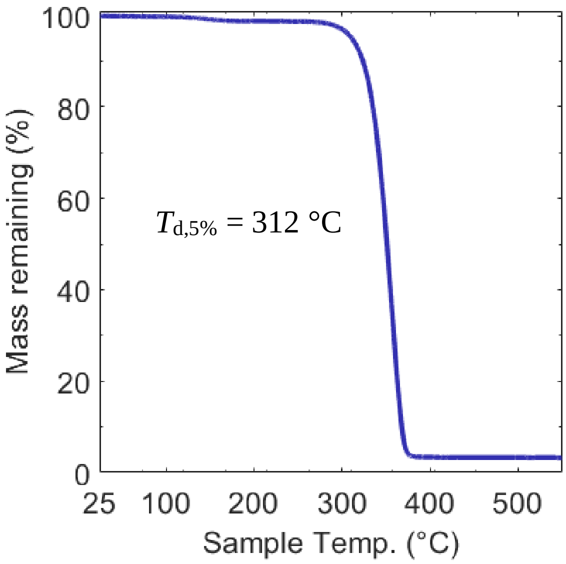

Figure S60. TGA spectrum of BO-*alt*-PA (**Table S14**, entry 2d) using air-free ChCl/EG heated from 25 °C – 550 °C.

6.2 TGA spectra of catalysts to determine its water content

TGA studies were used to identify the amount of water remaining in the choline salts and deep eutectic solvents. The sample was heated from room temperature to 100 °C and was left at 100 °C for 8 h to ensure that no water remained in the catalyst before continuing to ramp up the temperature. The mass loss seen at 100 °C was used to calculate the amount of water remaining in the catalyst.

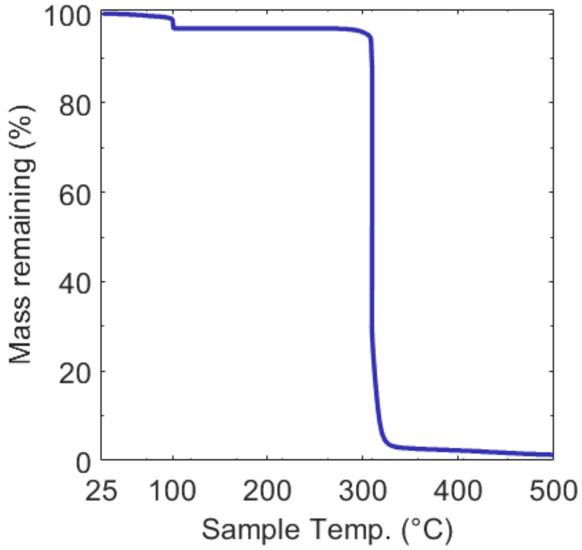

Figure S61. TGA spectrum of air-exposed ChCl heated from 25 °C – 500 °C. The sample was heated and held at 100 °C for 8h, then heated to the final temperature of 500 °C. The mass loss at 100 °C is accredited to loss of water from air-exposed choline chloride and was calculated to have approximately 0.43 equivalents of water for every choline cation.

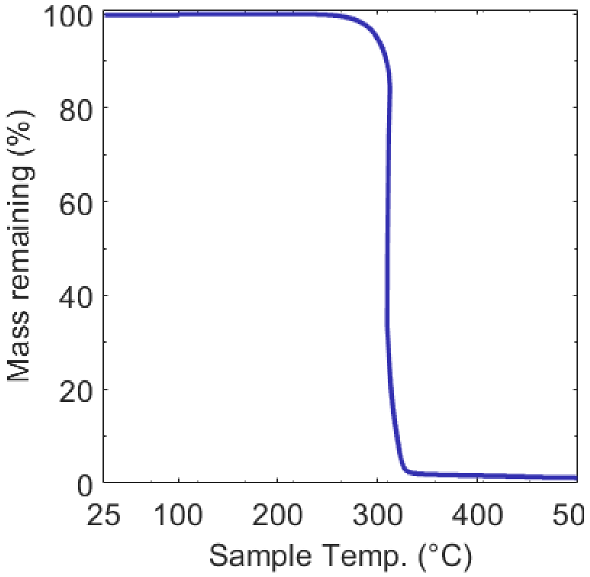

Figure S62. TGA spectrum of air-free ChCl heated from 25 °C – 500 °C. The sample was heated and held at 100 °C for 8h, then heated to the final temperature of 500 °C. The mass loss at 100 °C is accredited to loss of water from air-free choline chloride and was calculated to have no observable water for every choline cation.

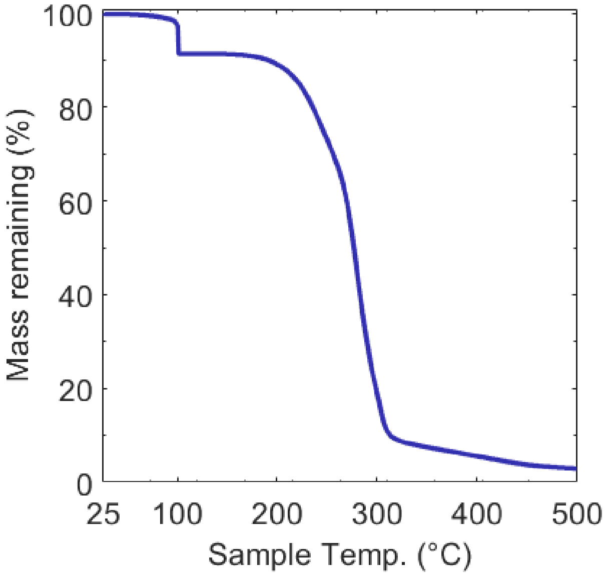

Figure S63. TGA spectrum of air-exposed ChCl/urea heated from 25 °C – 500 °C. The sample was heated and held at 100 °C for 8h, then heated to the final temperature of 500 °C. The mass loss at 100 °C is accredited to loss of water from air-exposed ChCl/urea and was calculated to have 1.17 equivalents of water for every choline cation.

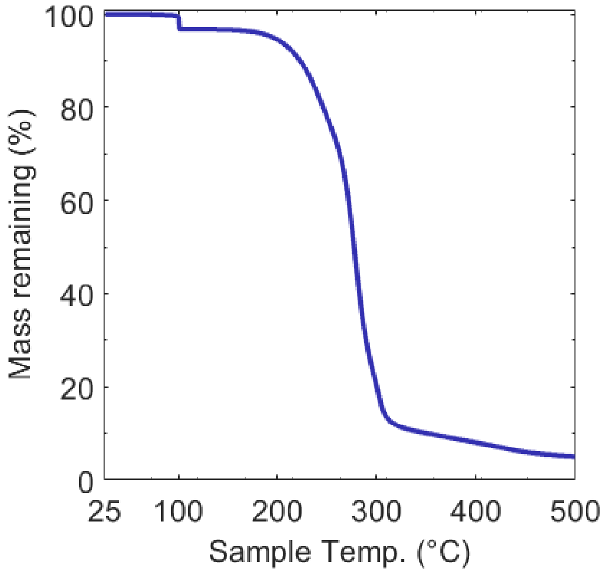

Figure S64. TGA spectrum of air-free ChCl/urea heated from 25 °C – 500 °C. The sample was heated and held at 100 °C for 8h, then heated to the final temperature of 500 °C. The mass loss at 100 °C is accredited to loss of water from air-free ChCl/urea and was calculated to have 0.41 equivalents of water for every choline cation.

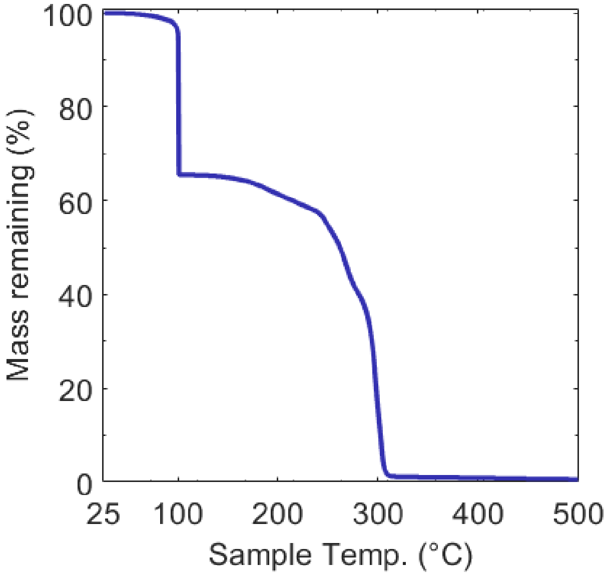

Figure S65. TGA spectrum of air-exposed ChCl/EG heated from 25 °C – 500 °C. The sample was heated and held at 100 °C for 8h, then heated to the final temperature of 500 °C. The mass loss at 100 °C is accredited to loss of water and some ethylene glycol (as seen on **Figure S67**), and thus cannot approximate the water content of air-exposed ChCl/EG.

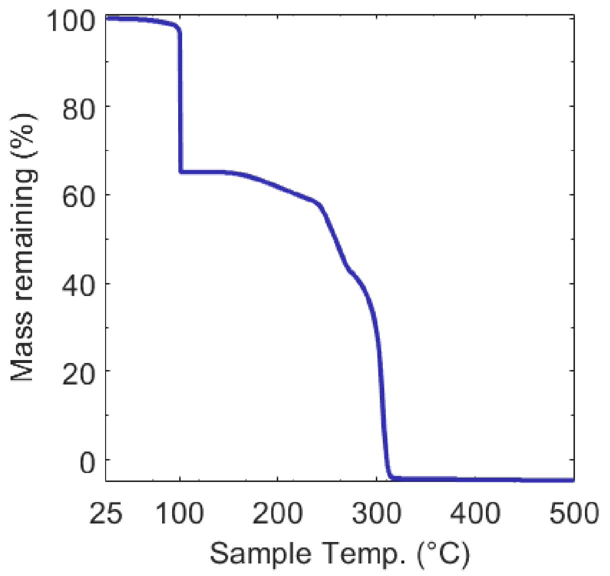

Figure S66. TGA spectrum of air-free ChCl/EG heated from 25 °C – 500 °C. The sample was heated and held at 100 °C for 8h, then heated to the final temperature of 500 °C. The mass loss at 100 °C is accredited to loss of water and some ethylene glycol (as seen on **Figure S67**), and thus cannot approximate the water content of air-free ChCl/EG.

6.3 TGA spectra of EG

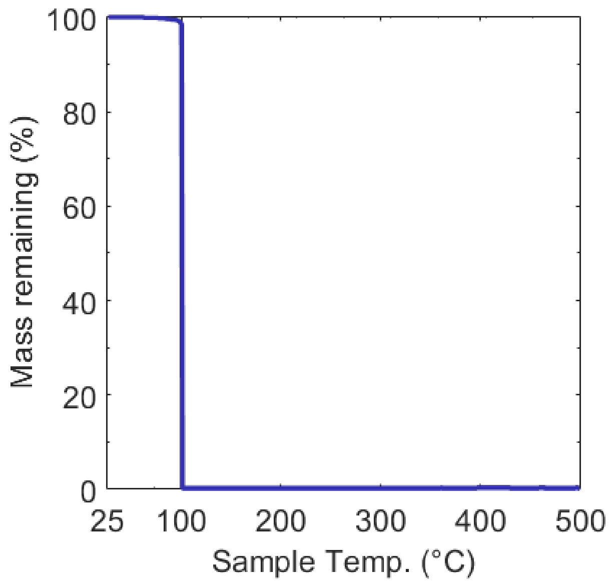

Figure S67. TGA spectrum of air-exposed EG heated from 25 °C – 500 °C. The sample was heated and held at 100 °C for 8h, then heated to the final temperature of 500 °C.

7. MALDI-TOF-MS data

For the MALDI-TOF-MS tables below, two matrices were used for the same polymer samples. Table S19 uses a trans-2-[3-(4-tert-butylphenyl)-2-methyl-2-propenylidene]malononitrile (DCTB) matrix and Table S20 uses a 2,5-dihydroxybenzoic acid (DHB) matrix. There was not enough sample left to perform MALDI-TOF analyses of air-free ChCl/urea and ChCl/EG with the DHB matrix.

Table S19. Repeat Unit MW (left) and End-Group MW (right) Averages via MALDI-TOF-MS Analysis using DCTB as the Matrix.[a]

DCTB Matrix						
	A		B		C	
**Air-Exposed**						
	repeat MW[b] (g/mol)	end group MW[c] (g/mol)	repeat MW[b] (g/mol)	end group MW[c] (g/mol)	repeat MW[b] (g/mol)	end group MW[c] (g/mol)
ChCl	236.0 (0.5)	103.0 (0.7)	236.4 (0.7)	30.9 (0.5)	235.0 (3.6)	204.5 (4.2)
ChCl/urea	236.0 (0.8)	102.1 (1.0)	235.0 (2.5)	31.3 (3.6)	236.0[d]	201.2[d]
ChCl/EG	236.1 (0.4)	102.3 (0.4)	235.0 (3)	32.2 (3)	238.0[e]	205.2[e]
**Air-Free**						
ChCl – main fragment	235.6 (3.6)	101.8 (1.2)	235.6 (5.2)	42.1 (3.9)	235.7 (2.6)	220.0 (3.0)
ChCl – minor fragment	232.6 (1.7)	81.3 (13.3)	-[f]	-[f]	233.1 (2.6)	203.8 (10.4)
ChCl/urea	235.5 (1.0)	99.3 (3.1)	234.6 (3.7)	34.1 (9.8)	234.6 (9.8)	205.5 (10.1)
ChCl/EG	235.6 (0.9)	99.5 (3.1)	233.5 (4.5)	30.6 (9.8)	235.0 (2.0)	197.3 (3.7)

[a]Analysis of three major peaks/species are presented. [b]Determined by taking the m/z difference between the peaks. [c]Determined by subtracting the maximum amount of repeat unit MW from the peak m/z. [d]Only two peaks were determined. [e]Only three peaks were determined. [f]The peaks were inconsistent, and thus, not analyzed.

Table S20. Repeat Unit MW (left) and End-Group MW (right) Averages via MALDI-TOF-MS Analysis using DHB as the Matrix.[a]

DHB Matrix						
	A		B		C	
**Air-Exposed**						
	repeat MW[b] (g/mol)	end group MW[c] (g/mol)	repeat MW[b] (g/mol)	end group MW[c] (g/mol)	repeat MW[b] (g/mol)	end group MW[c] (g/mol)
ChCl	236.1 (0.1)	103.2 (0.4)	236.1 (0.5)	31.1 (0.4)	236.0 (0.5)	210.9 (0.7)
ChCl/urea	236.1 (0.4)	103.0 (0.4)	236.1 (0.7)	32.4 (0.9)	235.9 (4)	226.4 (4)
ChCl/EG	236.1 (0.1)	103.1 (0.4)	236.1 (0.5)	45.7 (0.8)	236.0 (4.3)	227.2 (2.5)
**Air-Free**						
ChCl – main fragment	236.8 (0.3)	101.5 (2.1)	236.8 (0.3)	31.8 (2.2)	236.6 (1.1)	201.1 (1.4)
ChCl – minor fragment	236.6 (1.4)	105.7 (1.2)	236.4 (0.8)	30.0 (0.6)	-[d]	-[d]
ChCl - reflector mode	236.1 (0.03)	103.0 (0.5)	236.1 (0.4)	30.1 (0.7)	236.2 (0.7)	211.1 (0.5)

[a]Analysis of three major peaks/species are presented. [b]Determined by taking the m/z difference between the peaks. [c]Determined by subtracting the maximum amount of repeat unit MW from the peak m/z. [d]No third major peak was determined.

7.1 DCTB matrix

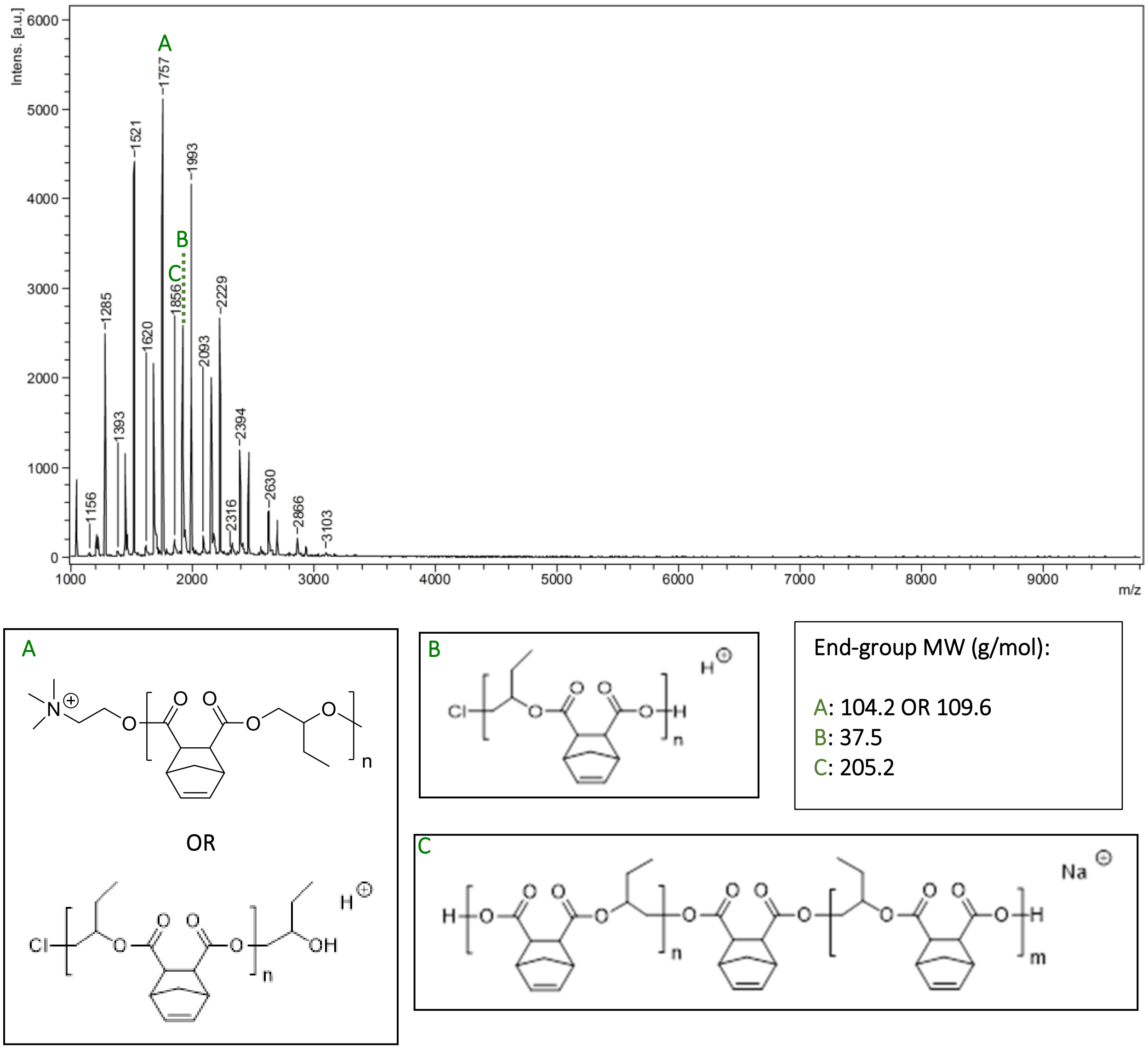

Figure S68. MALDI-TOF mass spectrum of BO-*alt*-CPMA synthesized using air-exposed ChCl (**Table S1**, entry 1c and **Table 2**, entry 1). Proposed end groups are shown.

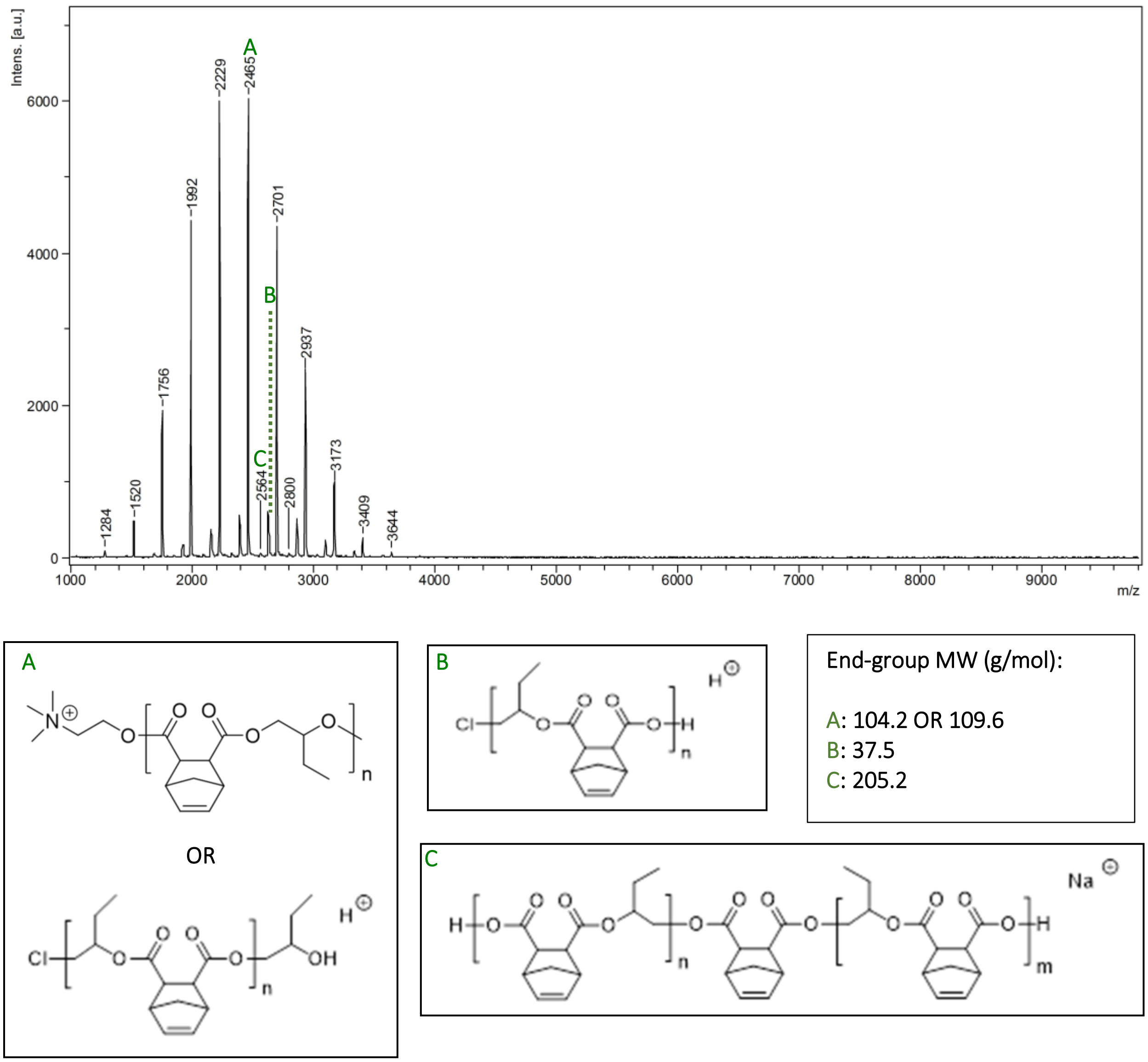

Figure S69. MALDI-TOF mass spectrum of BO-*alt*-CPMA synthesized using air-exposed ChCl/urea (**Table S4**, entry 1c and Table **2**, entry 2). Proposed end groups are shown.

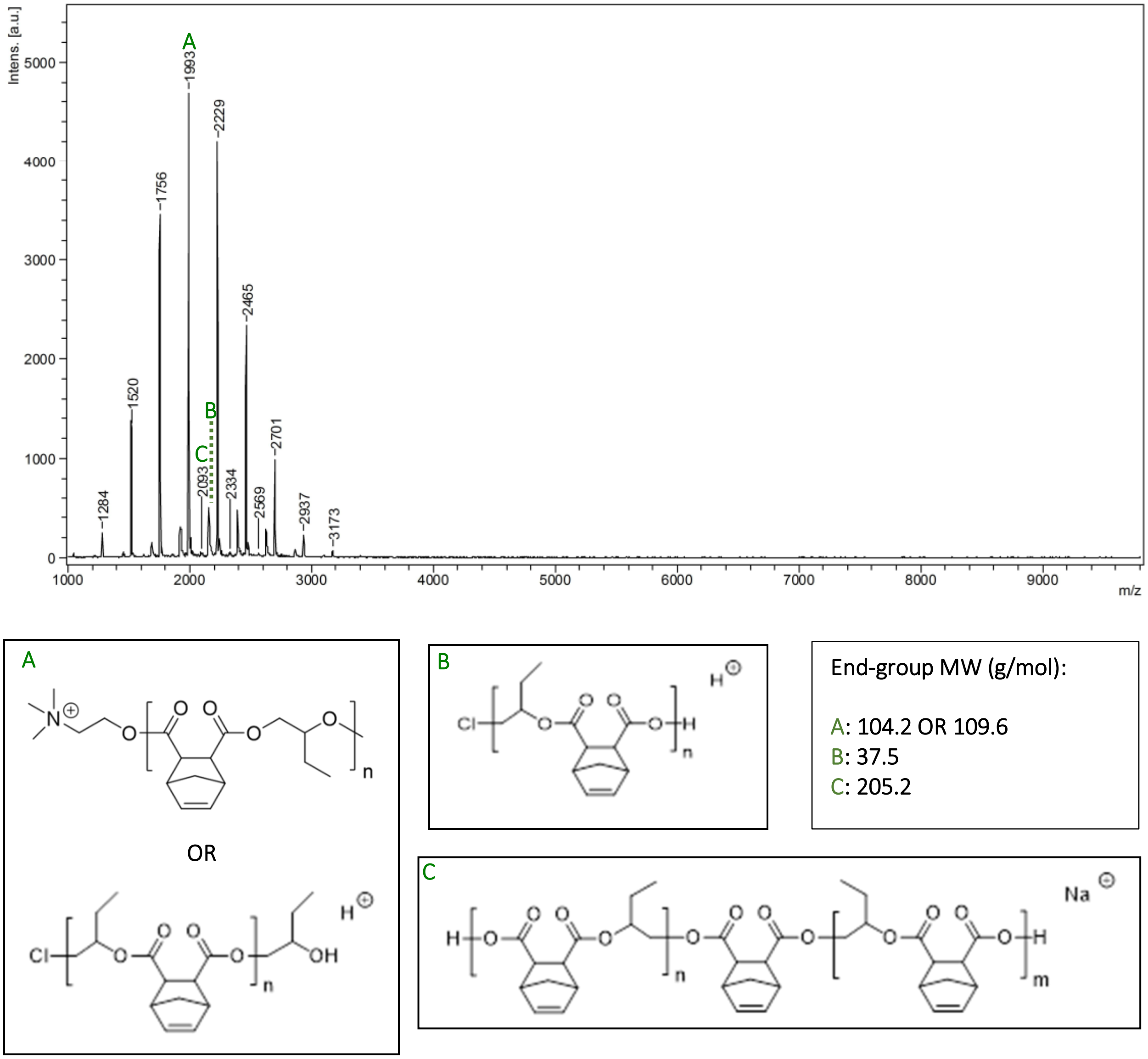

Figure S70. MALDI-TOF mass spectrum of BO-*alt*-CPMA synthesized using air-exposed ChCl/EG (**Table S5**, entry 1a and **Table 2**, entry 3). Proposed end groups are shown.

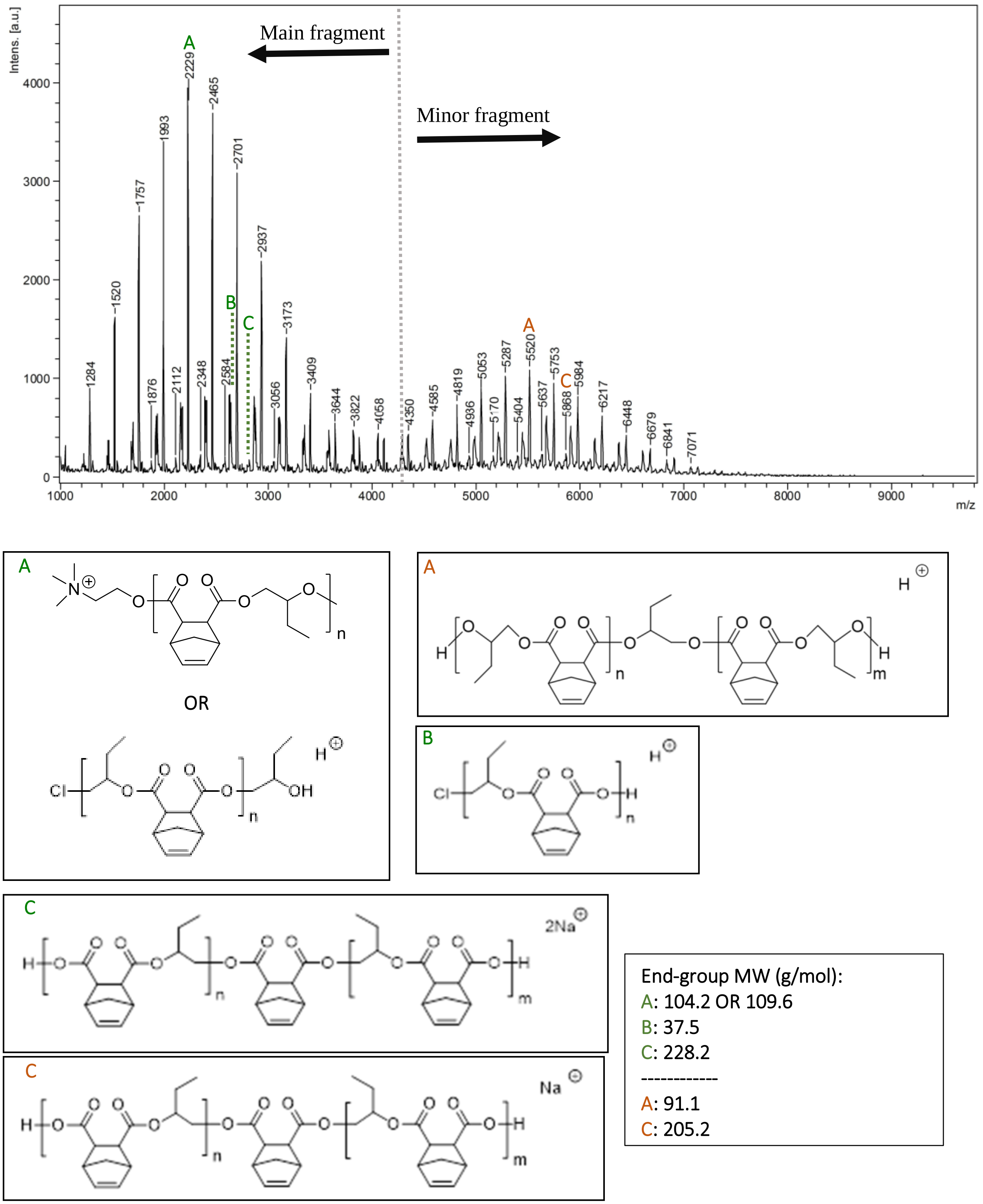

Figure S71. MALDI-TOF mass spectrum of BO-*alt*-CPMA synthesized using air-free ChCl (**Table** S10, entry 1d and **Table 2**, entry 4). Green peak labels are shown for the main fragment and the brown peak labels are shown for the minor fragment. Proposed end groups are shown.

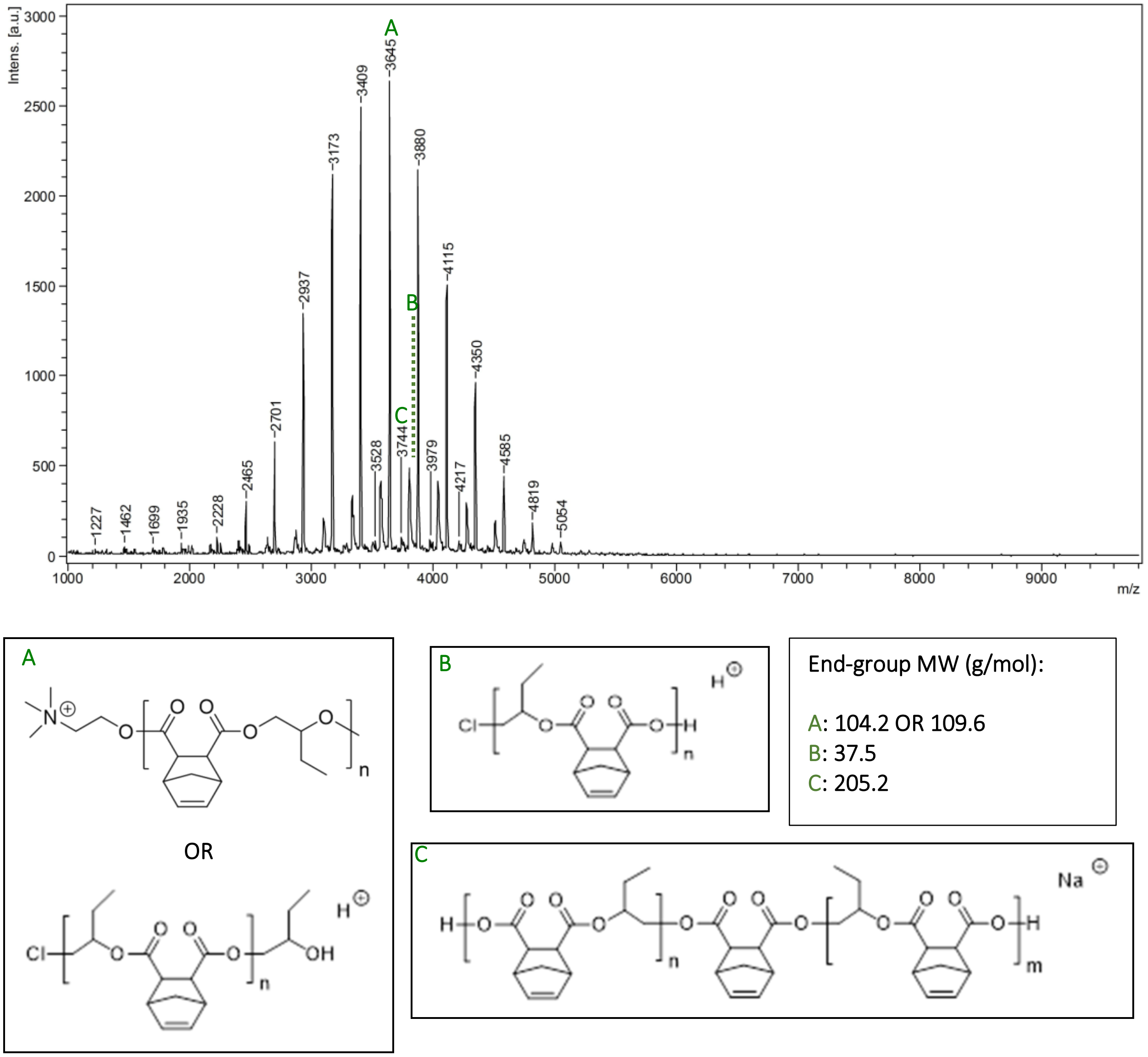

Figure S72. MALDI-TOF mass spectrum of BO-*alt*-CPMA synthesized using air-free ChCl/urea (**Table** S14, entry 1b and **Table 2**, entry 5). Proposed end groups are shown.

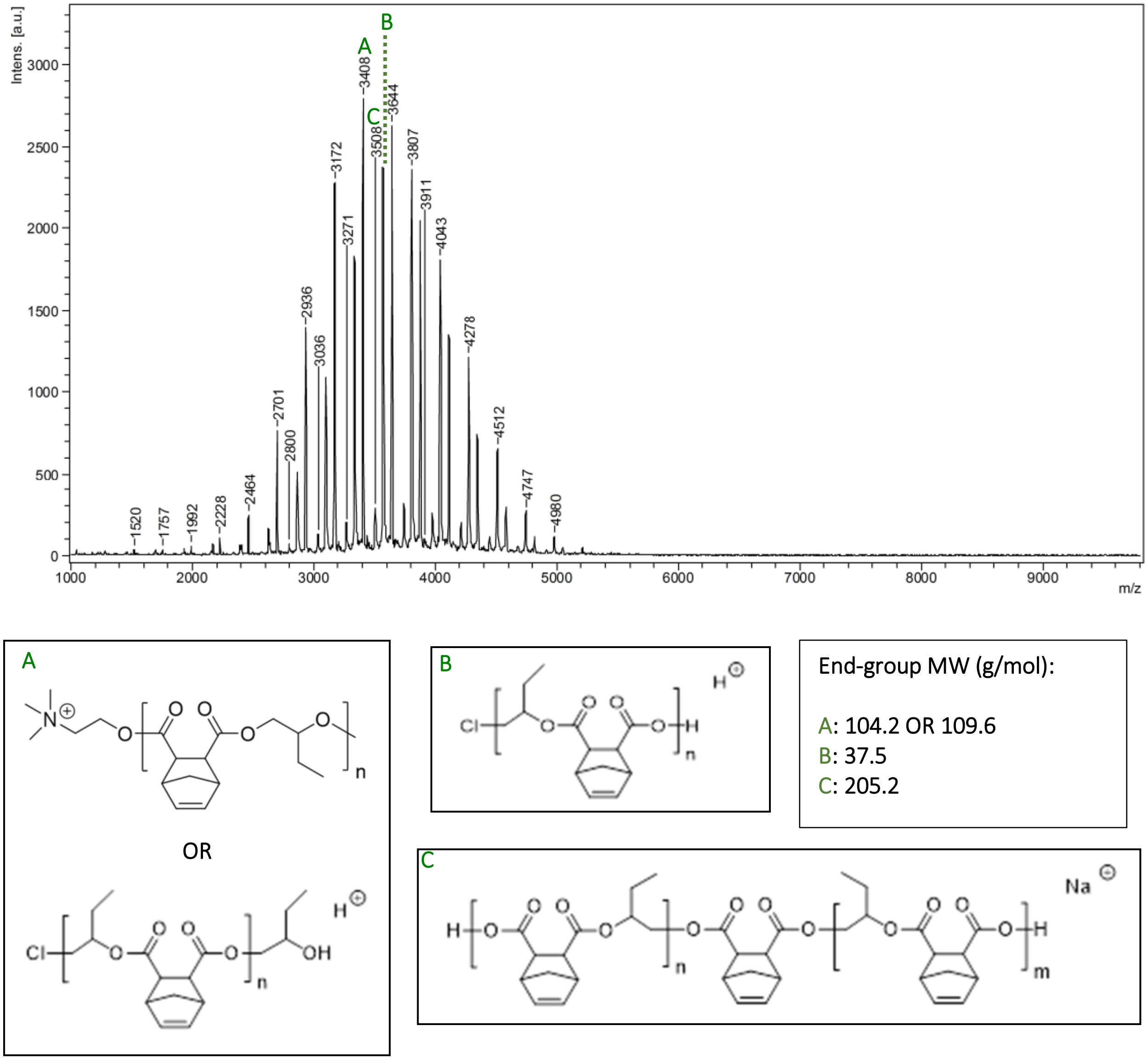

Figure S73. MALDI-TOF mass spectrum of BO-*alt*-CPMA synthesized using air-free ChCl/EG (**Table S15**, entry 1b and **Table 2**, entry 6). Proposed end groups are shown.

7.2 DHB matrix

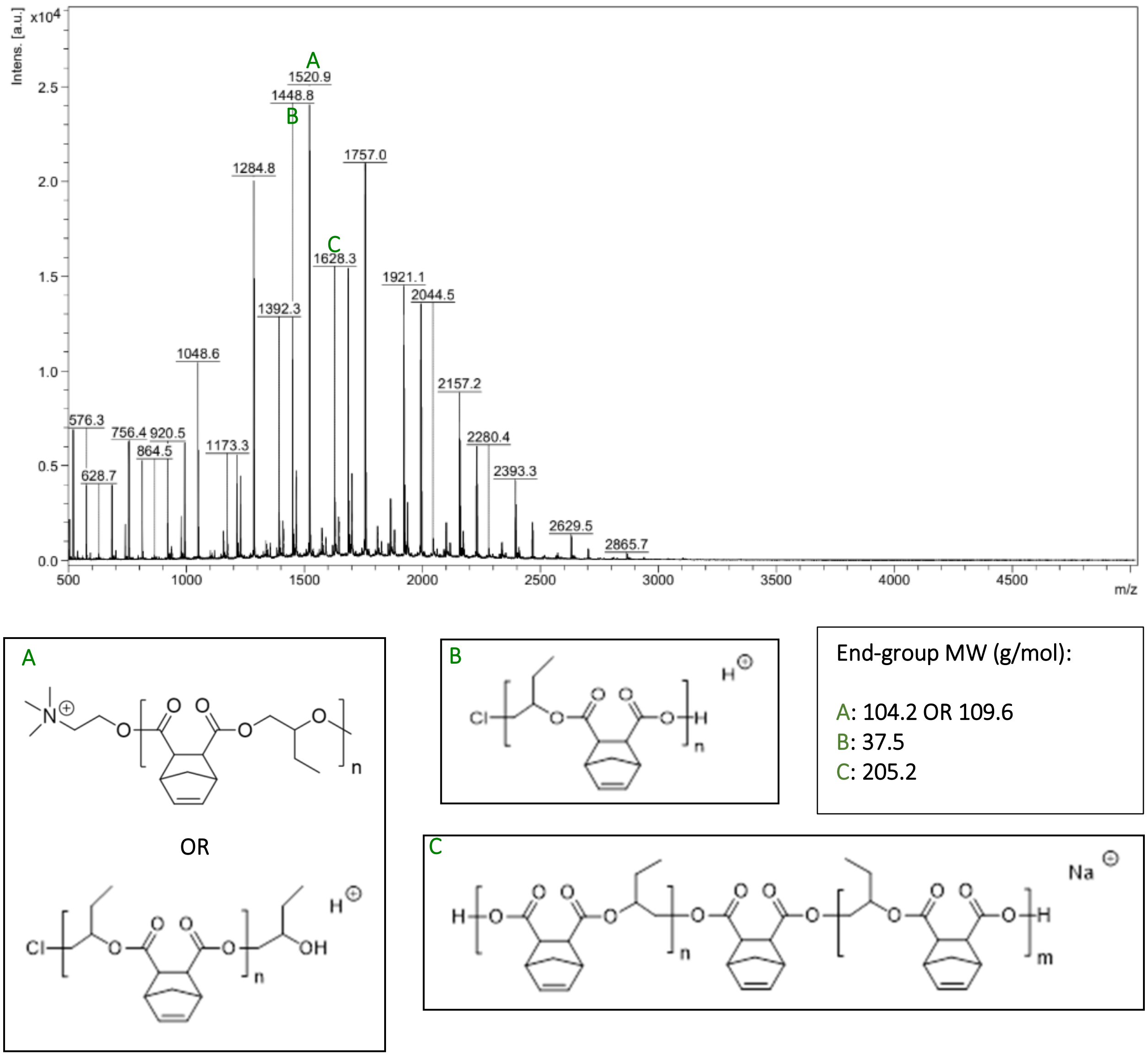

Figure S74. MALDI-TOF mass spectrum of BO-*alt*-CPMA synthesized using air-exposed ChCl (**Table S1**, entry 1c and **Table 2**, entry 1). Proposed end groups are shown.

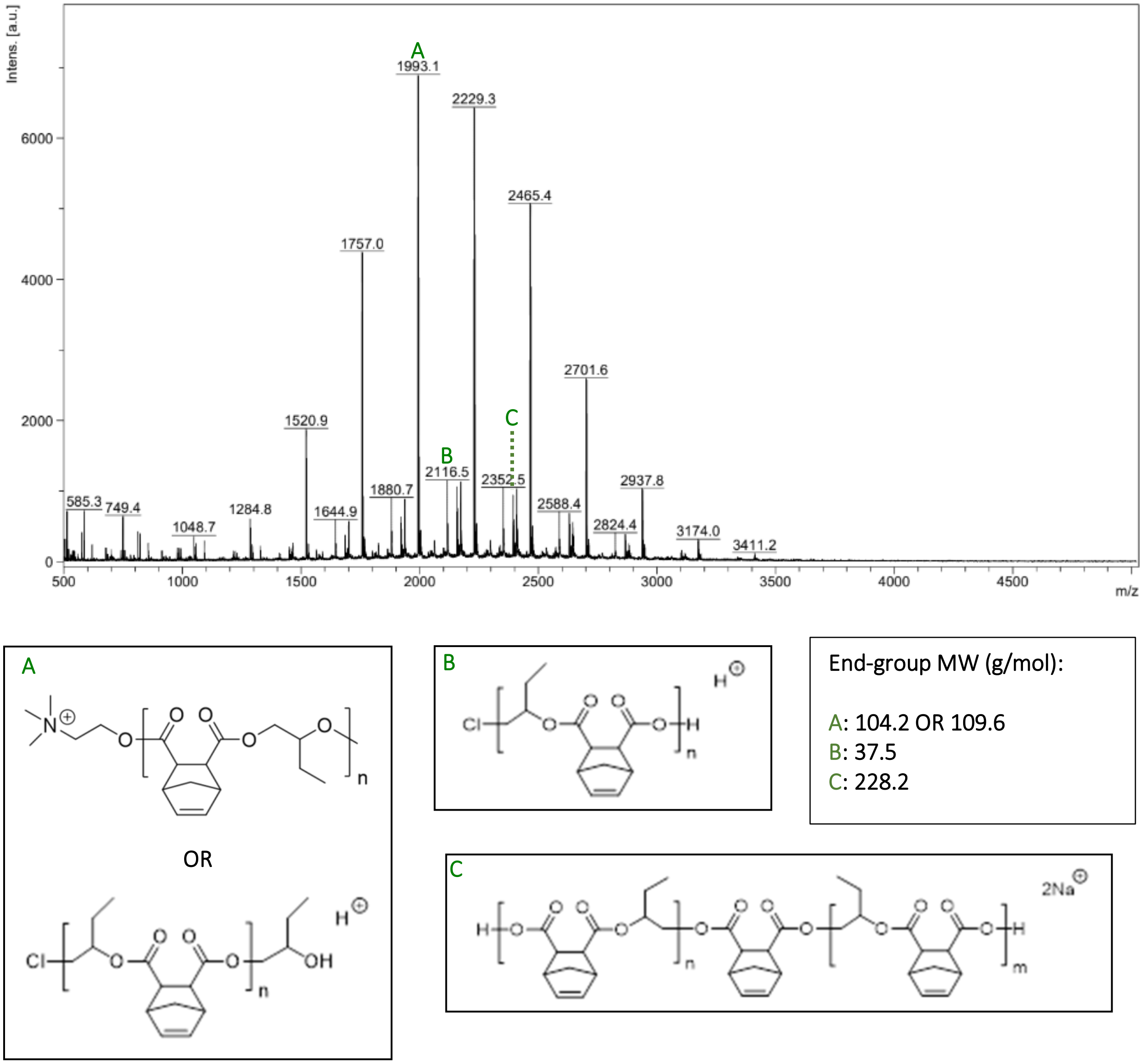

Figure S75. MALDI-TOF mass spectrum of BO-*alt*-CPMA synthesized using air-exposed ChCl/urea (**Table S4**, entry 1c and **Table 2**, entry 2). Proposed end groups are shown.

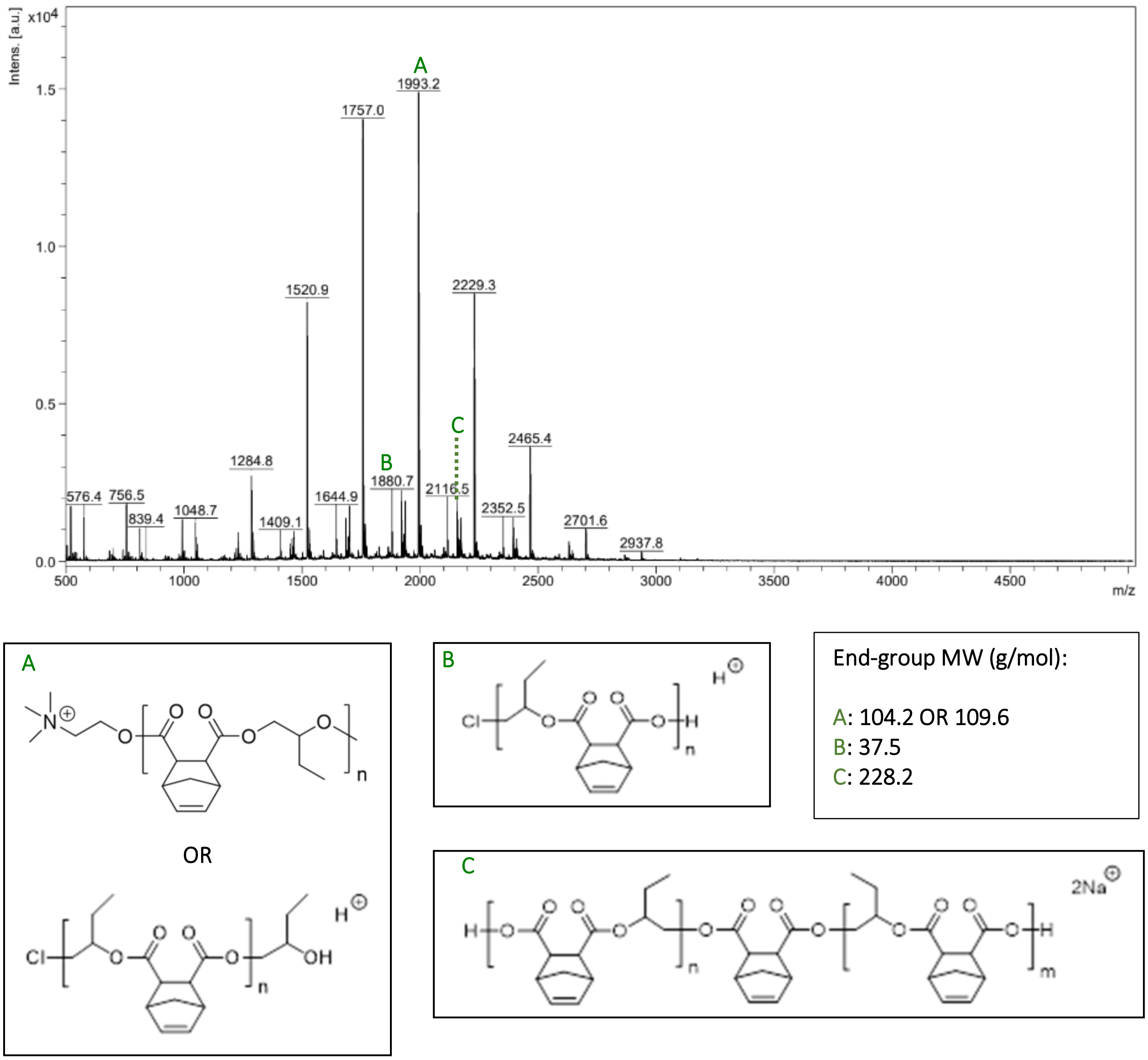

Figure S76. MALDI-TOF mass spectrum of BO-*alt*-CPMA synthesized using air-exposed ChCl/EG (**Table S5**, entry 1a and **Table 2**, entry 3). Proposed end groups are shown.

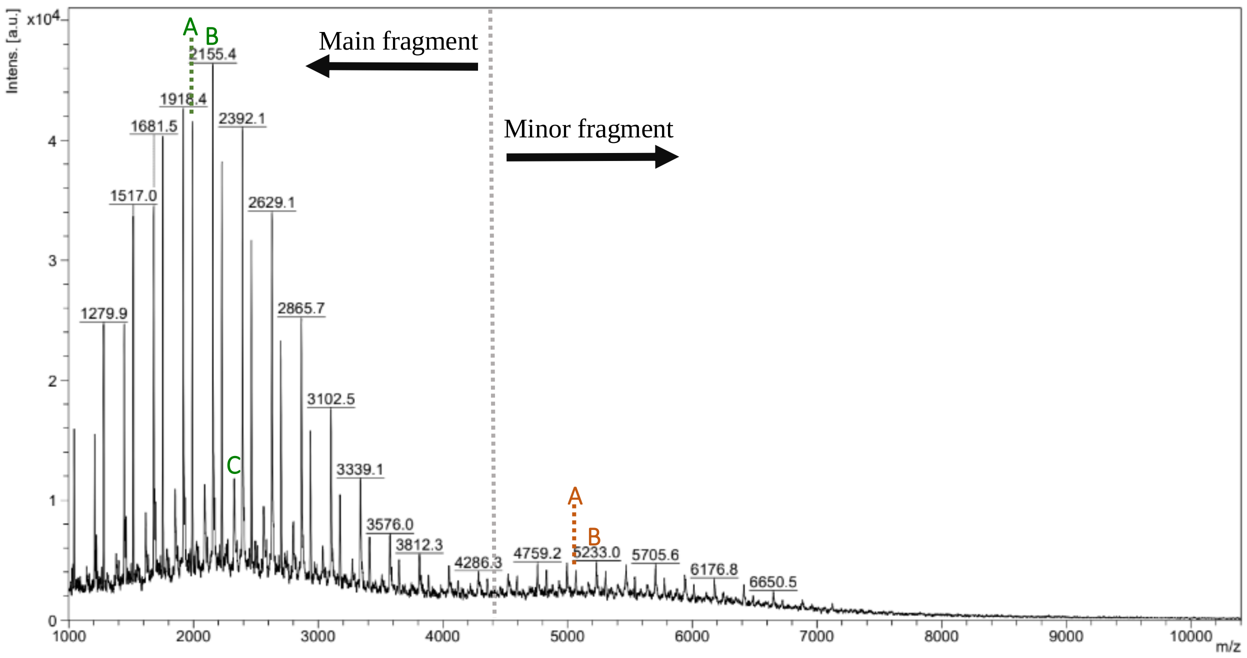

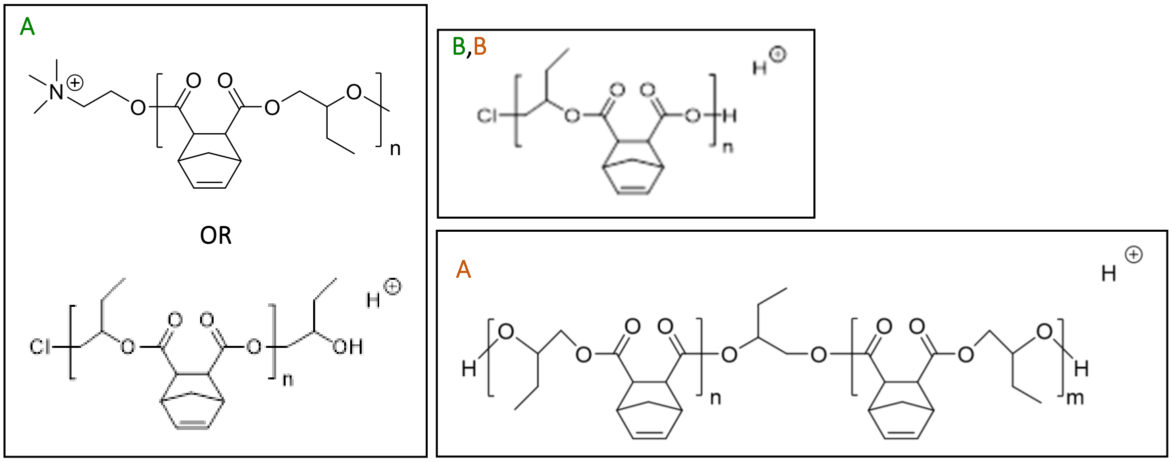

End-group MW (g/mol):
A: 104.2 OR 109.6
B: 37.5
C: 205.2

A: 91.1
B: 37.5

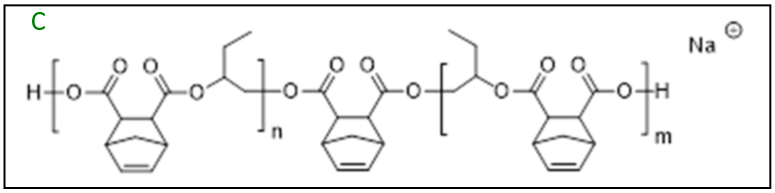

Figure S77. MALDI-TOF mass spectrum of BO-*alt*-CPMA synthesized using air-free ChCl (**Table S10**, entry 1d and **Table 2**, entry 4). Green peak labels are shown for the main fragment and the brown peak labels are shown for the minor fragment. Proposed end groups are shown.

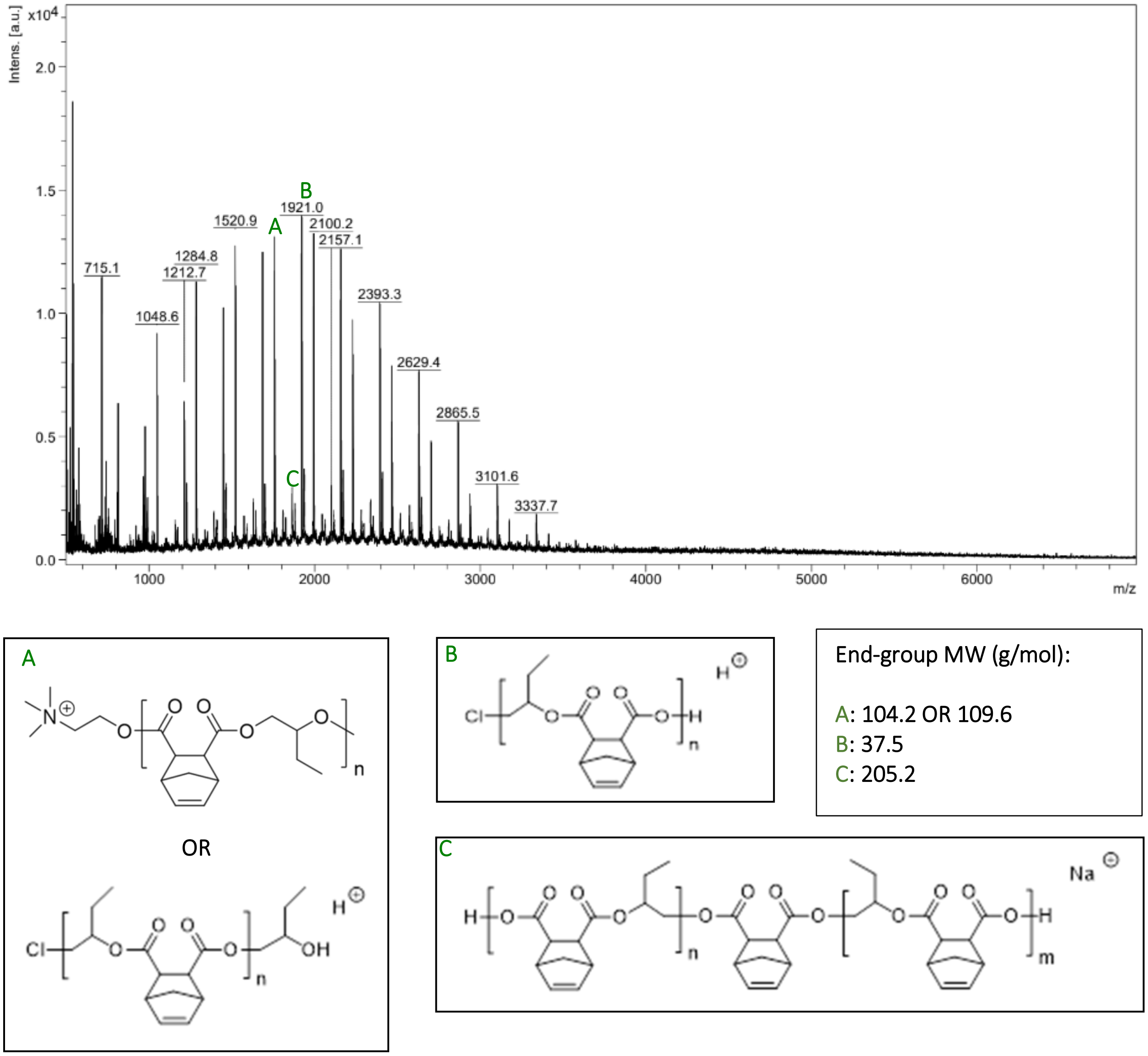

Figure S78. MALDI-TOF mass spectrum of BO-*alt*-CPMA synthesized using air-free ChCl in reflector mode (**Table** S10, entry 1d and **Table 2**, entry 4). Proposed end groups are shown.

References

[1] Wyatt Technology, https://wyatttechnology.zendesk.com/hc/en-us/articles/11522772454163-How-do-I-determine-what-dn-dc-I-should-use-for-my-sample-, (accessed September 2023).
